# Glycolytic heterogeneity drives metabolic-targeted therapy in pancreatic ductal adenocarcinoma

**DOI:** 10.1038/s41392-025-02546-8

**Published:** 2026-01-20

**Authors:** Ugo Chianese, Chiara Papulino, Gerardo Saggese, Ahmad Ali, Marianna Ciotola, Enza Lonardo, Mirko Cortese, Gregorio Favale, Annabella Di Mauro, Danila La Gioia, Valentina Golino, Eduardo Sommella, Pietro Campiglia, Renato Franco, Fortunato Ciardiello, Ferdinando De Vita, Vincenzo Carafa, Lucia Altucci, Rosaria Benedetti

**Affiliations:** 1 Department of Precision Medicine, University of Campania “Luigi Vanvitelli”, Naples, Italy; 2 Institute of Genetics and Biophysics ‘Adriano Buzzati-Traverso’ (IGB), CNR, Naples, Italy; 3 Department of Environmental, Biological and Pharmaceutical Sciences and Technologies, University of Campania “Luigi Vanvitelli”, Caserta, Italy; 4 Pathology Unit, National Cancer Institute – IRCCS G. Pascale Foundation, Naples, Italy; 5 Department of Pharmacy (DIFARMA), University of Salerno, Salerno, Italy; 6 Department of Mental and Physical Health and Preventive Medicine, University of Campania “Luigi Vanvitelli”, Naples, Italy; 7 Biogem Institute of Molecular and Genetic Biology, Ariano Irpino, Italy; 8 Program of Medical Epigenetics, Vanvitelli Hospital, Naples, Italy

**Keywords:** Cancer metabolism, Cancer therapy, Molecular biology

## Abstract

Pancreatic ductal adenocarcinoma is traditionally characterized as a glycolytic tumor. However, the extent and clinical relevance of its metabolic heterogeneity remain poorly understood. In this study, we investigated whether glycolytic activity follows a consistent expression pattern across pancreatic ductal adenocarcinoma patients and explored how metabolic diversity influences therapeutic responses. Using spatial transcriptomics of ex vivo primary human pancreatic ductal adenocarcinoma specimens, along with single-cell and bulk RNA sequencing, we mapped glycolytic heterogeneity within the tumor microenvironment. Patient-derived cell models representing distinct glycolytic phenotypes were employed to assess metabolic profiles and responses to glycolytic pathway inhibition. A multiomics approach—including metabolomics, proteomics, and lipidomics—was integrated through a robust bioinformatics pipeline to identify pathway-specific variations. Our findings revealed pronounced glycolytic heterogeneity across pancreatic ductal adenocarcinoma tumors, with distinct transcriptional profiles that maintained cellular identity and spatial architecture. These glycolytic patterns are associated with clinical outcomes, suggesting their potential as prognostic indicators. Functional studies confirmed differential sensitivity to metabolic inhibitors in organoids and demonstrated their safety across models, supporting the therapeutic relevance of glycolytic stratification. Overall, this study reveals clinically significant metabolic heterogeneity in pancreatic ductal adenocarcinoma and proposes a glycolysis-based framework for patient stratification, which could guide personalized metabolic therapies and advance precision oncology in pancreatic cancer.

## Introduction

Pancreatic ductal adenocarcinoma (PDAC) is the most prevalent and aggressive form of pancreatic cancer and accounts for most pancreatic malignancies diagnosed worldwide. Despite continuous advances in diagnostic imaging, surgical techniques, and systemic treatments, PDAC remains characterized by a dismal prognosis and extremely low long-term survival rates.^1^ One of the main reasons for this poor clinical outcome lies in the intrinsically silent nature of the disease during its early stages: patients often exhibit vague or nonspecific symptoms, which delays clinical suspicion and results in most diagnoses occurring only once the tumor has already reached an advanced or metastatic stage.^2^ In addition, PDAC shows profound intrinsic and acquired resistance to standard therapies, further contributing to its high lethality and making it one of the most challenging solid tumors to treat. This therapeutic resistance is compounded by the rapid evolutionary dynamics of PDAC cells, which can swiftly adapt to therapeutic pressures, and by the limited availability of effective biomarkers capable of predicting treatment response or identifying patients who may benefit from targeted/immunological interventions.

A key contributor to the complexity and resilience of PDAC is its uniquely dense and desmoplastic tumor microenvironment (TME). This microenvironment, which is markedly more prominent than in many other solid tumors, consists of a highly heterogeneous mixture of stromal, immune, and endothelial cells embedded within an abundant extracellular matrix. Together, these components form a dynamic ecosystem that not only supports tumor growth, invasion, and metastasis but also acts as a physical and biochemical barrier that hinders the efficacy of therapeutic agents. Cancer-associated fibroblasts, immunosuppressive myeloid cells, and a variety of T-cell subsets collectively participate in shaping this environment, fostering immune evasion and metabolic competition. The high interstitial pressure and poor vascular perfusion typical of PDAC further exacerbate drug delivery limitations, while chronic inflammation within the TME promotes continuous remodeling and reinforces pro-tumorigenic signaling pathways.

Improving PDAC outcomes thus requires innovative, multidisciplinary approaches capable of dissecting the complex interplay between malignant cells and their surrounding stroma to uncover novel therapeutic targets and cell-type-specific vulnerabilities. Despite advances in genomic and transcriptomic profiling, a critical yet underexplored area remains the investigation of cell-specific metabolic programs within the TME. It is now well established that cancer cells undergo profound metabolic reprogramming to support rapid proliferation, survival under stress, and immune evasion. However, these metabolic adaptations are not uniform across all tumor cells, nor are they homogeneous across stromal and immune populations. The heterogeneity of metabolic phenotypes is shaped by factors such as genetic mutations, cellular differentiation states, and microenvironmental conditions such as hypoxia and nutrient availability. Such heterogeneity has important therapeutic implications: if different subpopulations within a tumor rely on distinct metabolic pathways, then inhibiting a single metabolic process may only affect a subset of cells, allowing others to survive and drive recurrence. For this reason, dissecting metabolic interactions at single-cell resolution has become increasingly recognized as a crucial step toward understanding how tumors coordinate nutrient acquisition, redistribute metabolic resources, and exploit stromal components to sustain growth even under extreme environmental constraints.

This study builds upon and complements recent publications that collectively emphasize the significant metabolic heterogeneity within PDAC, identifying distinct metabolic subtypes with differing glucose, lipid, and glutamine metabolism that correlate with molecular classification, prognosis, and drug sensitivity.^3–9^ These findings underscore the need to move beyond bulk tumor profiling toward more refined, cell-resolved analyses capable of capturing the spatial and functional heterogeneity that defines PDAC metabolism. Then, our study advances the field by integrating spatial transcriptomics with multiomics and functional validation to reveal how metabolic heterogeneity (especially glycolysis) is spatially organized in PDAC and how it correlates with microenvironmental factors such as hypoxia, vascular distribution, and stromal density. Through this strategy, we identify distinct cell-type-specific metabolic dependencies that may represent actionable therapeutic targets. This spatially resolved, cell-specific metabolic perspective is a key novelty compared with prior bulk or subtype classification approaches. Furthermore, a robust in vitro multiomics approach revealed differential sensitivity to the metabolic inhibitor of lactate dehydrogenase A (LDHA-i), supporting the presence of glycolytic vulnerability in PDAC. Moreover, we evaluate the anticancer efficacy and safety of LDHA inhibition across advanced preclinical PDAC models, including three-dimensional culture systems and in vivo settings. These experiments not only confirm the potential therapeutic value of targeting glycolysis but also highlight the importance of optimizing metabolic inhibitors to maximize antitumor effects while minimizing off-target toxicity. By demonstrating both the feasibility and therapeutic promise of LDHA-targeting strategies, our study lays essential groundwork for future drug development efforts aimed at exploiting metabolic vulnerabilities in PDAC. Together, these findings contribute to a more comprehensive and spatially informed understanding of the metabolic landscape of PDAC. By characterizing how metabolic programs vary across different tumor regions and cell types, and by linking these programs to functional drug responses, our study highlights the potential for developing personalized therapeutic strategies based on metabolic profiling. Ultimately, such approaches may help overcome the formidable resistance mechanisms that characterize PDAC and could pave the way for more effective, tailored treatments for patients afflicted with this devastating disease. In the near future, as spatially resolved and multiomics technologies continue to evolve, integrating metabolic, immunologic, and stromal signatures will likely become essential for refining patient stratification and guiding the design of next-generation therapeutic interventions, bringing us closer to precision medicine approaches capable of addressing the unique biological challenges posed by PDAC.

## Results

### PDAC cellular identity is associated with the metabolic profile and glycolytic heterogeneity

**Fig. 1:**
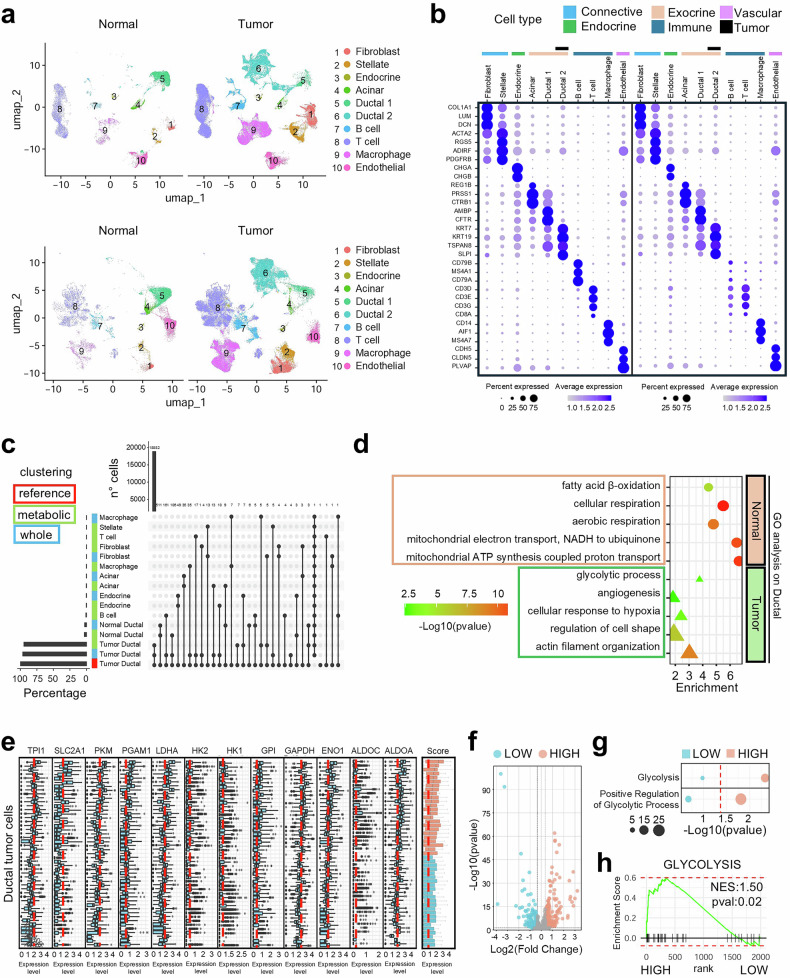
Metabolic Gene Expression Highlights Functional and Cellular Heterogeneity in PDAC. **a** UMAP plot showing PDAC and normal pancreatic cells clustered for whole gene expression (above) and metabolic genes (below). **b** Dot plot of representative marker genes for each cell cluster. Color intensity reflects the relative expression of the gene, while size is proportionate to the fraction of cells expressing a given gene. **c** Upset plot of intersection for cell type attribution, reference paper, metabolic genes, and whole gene expression. **d** Statistically significant results from GO analysis for biological processes in normal and tumor ductal cells. **e** Log_2_-normalized expression values of the glycolytic gene signature across tumor ductal cells. Patients were stratified into High (red) and Low (light blue) glycolysis groups based on the median glycolytic signature score. The red dashed line indicates the mean expression within the cohort. **f** Volcano plot showing genes differentially expressed between the High and Low glycolysis groups. Red and light-blue dots indicate genes significantly upregulated in the High and Low groups, respectively. The *x*- and *y*-axes represent log₂ fold change and −log_10_ *p* value, respectively. **g** Gene Ontology (GO) enrichment analysis of genes upregulated in the High and Low groups. Pathways associated with “glycolysis” and “positive regulation of glycolytic process” are highlighted. The *x*-axis represents transformed significance (−log_10_ *p* value), and dot size indicates the odds gene ratio. **h** GSEA enrichment plot for the Hallmark Glycolysis pathway comparing High and Low groups, showing significant enrichment of glycolytic genes in the High-glycolysis samples

To comprehensively explore the link between metabolism and cell type composition in PDAC, single-cell RNA sequencing (scRNA-seq) datasets from 47 tumor and 16 normal pancreas samples were analyzed. Cell types were inferred by clustering the data via two approaches: one based on the full transcriptome and the other restricted to metabolic genes (Fig. 1a, Supplementary Fig. 1a). Canonical markers were then applied to define the identities of the cells within each cluster. Both methods revealed consistent cellular components: the connective cluster (fibroblasts and stellate cells), the endocrine and exocrine clusters (including acinar and ductal cells), the immune cluster (B cells, T cells, and macrophages), and the vascular endothelial component (Fig. 1b). Importantly, the cell type proportions were highly concordant between the two clustering strategies (Supplementary Fig. 1b), supporting the robustness of the analysis. PDAC samples presented a greater abundance of immune components, an expanded connective population, and a reduced presence of endothelial and exocrine cells. PDAC tumor cells were consistently classified via both approaches (Fig. 1c), revealing a distinct metabolic expression profile linked to their cellular identity. Comparative analysis of key biological processes between normal and tumor ductal cells revealed two clearly defined metabolic phenotypes: normal ductal cells presented enrichment in mitochondrial pathways associated with aerobic respiration and fatty acid β-oxidation, whereas tumor ductal cells presented increased glycolytic activity (Fig. 1d). Additionally, the expression of glycolytic genes, including *ALDOA* and *LDHA*, was elevated in the cancer ductal population and colocalized with tumor markers (Supplementary Fig. 1c). As shown in Supplementary Fig. 1d, the UMAP projections of tumor ductal cells demonstrate that patient-specific centroids (large colored circles) remain clearly separated across the embedding space in both the whole-transcriptome and glycolysis-focused embeddings. The mean interpatient centroid distances were consistently large, and the distribution of these distances was centered well above zero, indicating that Harmony integration did not collapse patient-specific transcriptional structures. A permutation test based on 1000 random shufflings of patient labels further confirmed that the observed separation far exceeded that expected under a randomized null model (*p* < 0.001), demonstrating that the interpatient transcriptional heterogeneity is statistically significant and not attributable to stochastic variation. To determine whether this heterogeneity reflects biologically meaningful differences, we stratified patients on the basis of the mean log₂-normalized expression of the glycolytic gene signature in tumor ductal cells. Patients whose glycolytic scores exceeded the cohort median were assigned to the high-glycolysis group, whereas the remainder formed the low-glycolysis group (Fig. 1e). Differential expression analysis revealed a distinct transcriptional signature between these groups (Fig. 1f), with genes upregulated in the high-glycolysis group significantly enriched for pathways associated with glycolysis and positive regulation of glycolytic processes (Fig. 1g). In contrast, the genes in the low-glycolysis group were not enriched in these pathways. Consistently, gene set enrichment analysis (GSEA) of hallmark gene sets revealed significant enrichment of the glycolysis pathway in the high-glycolysis group (NES = 1.50, adjusted *p* < 0.02) (Fig. 1h). Together, these results reinforce the association between metabolic traits and cell identity, showing that tumor samples differ substantially in their glycolytic transcriptional programs across patients, which has emerged as a key feature of interpatient heterogeneity in PDAC.

### Preserved PDAC tissue architecture indicates glycolytic variability in patients

**Fig. 2:**
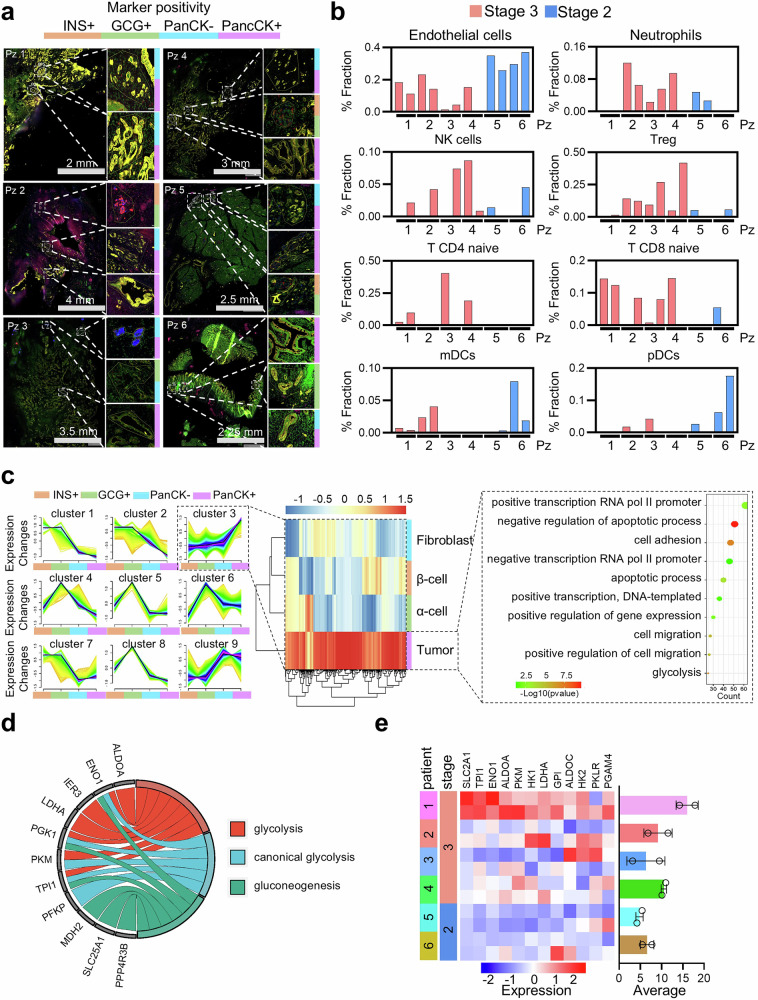
Spatial Profiling Uncovers Glycolytic Signatures and TME Heterogeneity in PDAC. **a** PDAC patient samples stained for INS^+^, GCG^+^, PanCK^−^ and PanCK^+^ markers and processed for spatial transcriptomics. White squares highlight areas of interest for gene expression acquisition. Colored bords refer to marker positivity INS^+^ (orange), GCG^+^ (green), PanCK^−^ (light blue), and PanCK^+^ (purple). **b** Histograms of deconvoluted spatial transcriptomics data displaying TME cell type percentage in PDAC patients, stratified for tumor stage. **c** Modules of gene expression across cell types highlight highly expressed genes in tumor cells, along with Gene Ontology (GO) analysis displaying statistically significant biological processes. Label below each cluster refers to marker positivity INS^+^ (orange), GCG^+^ (green), PanCK^−^ (light blue), and PanCK^+^ (purple). **d** Circos plot of glucose-related activity derived GO analysis. **e** Heatmap depicting glycolytic signature expression in PDAC patients, with two Areas of Interest (AOIs) per patient, resulting in two rows for each patient. Data are represented as mean ± SD

To elucidate how spatial organization and the cellular composition within the TME reflect and potentially drive PDAC progression, we performed an integrated spatial and transcriptional analysis across tumor stages and patient samples. Six PDAC primary samples were profiled (Fig. 2a), allowing the identification of tumor regions as well as fibroblastic and endocrine components (Supplementary Fig. 2a). By evaluating the geometric distance between cell types, we found that in three out of six patients (specimens 2, 5, and 6), the endocrine component was located further from tumor cells than was the connective tissue. In contrast, the fibroblast population was consistently closer to the tumor cell population, corroborating its established role in supporting tumor progression (Supplementary Fig. 2b). Additionally, spatial transcriptomics data were deconvoluted to estimate the relative abundance of cell types within the TME, and their distribution across tumor stages was examined (Fig. 2b). In stage T3, endothelial cell abundance was lower than that in stage T2, whereas neutrophils, NK cells, and Tregs were prevalent in T3. Stage T3 PDAC specimens also presented increased infiltration of naïve CD4^+^ and CD8^+^ T cells. However, conventional dendritic and plasmacytoid dendritic cells, which are key in activating naïve T cells, were globally reduced compared with those in T2 specimens. The composition of the infiltrate and the prevalence of glycolytic markers were also evaluated via an orthogonal approach through immunohistochemistry performed on the same tissues previously analyzed by spatial transcriptomics vs the nontumoral paired adjacent pancreatic tissues. Additionally, in this case, we found that all patients presented elevated numbers of CD4- and CD8-positive cells. In general, compared with adjacent tissue samples, all PDAC samples presented increased immune cell infiltration (Supplementary Fig. 2c). These observations suggest that PDAC progression is associated with a proinflammatory environment marked by immune cell infiltration but with impaired or ineffective immune activation. This finding is consistent with the reported poor response to immunotherapy^10^ and the progressive loss of endothelial components essential for proper pancreatic cell differentiation.^11^ To further investigate the molecular underpinnings of these spatial features, we analyzed the gene expression profiles of distinct cell types, identifying a shared set of highly expressed genes across PDAC populations. Gene Ontology (GO) analysis linked these genes to processes such as negative regulation of apoptosis and glycolytic activity (Fig. 2c). Key genes involved in glycolysis, such as *LDHA*, *ALDOA*, *ENO1*, *PKM*, and *PFKP*, were significantly expressed (Fig. 2d). Interestingly, the expression of this newly identified glycolytic signature varied among PDAC patients and was correlated with clinical characteristics (Fig. 2e). Specifically, the signature was directly proportional to tumor stage and associated with an immunosuppressive microenvironment. These findings support the molecular association between PDAC progression and hallmarks of glycolysis, strengthening its clinical stage-related heterogeneity among patients and highlighting its impact on spatial organization and the TME.

### The highest expression levels of glycolytic genes in PDAC are associated with the highest hypoxia score and the worst survival rate

**Fig. 3:**
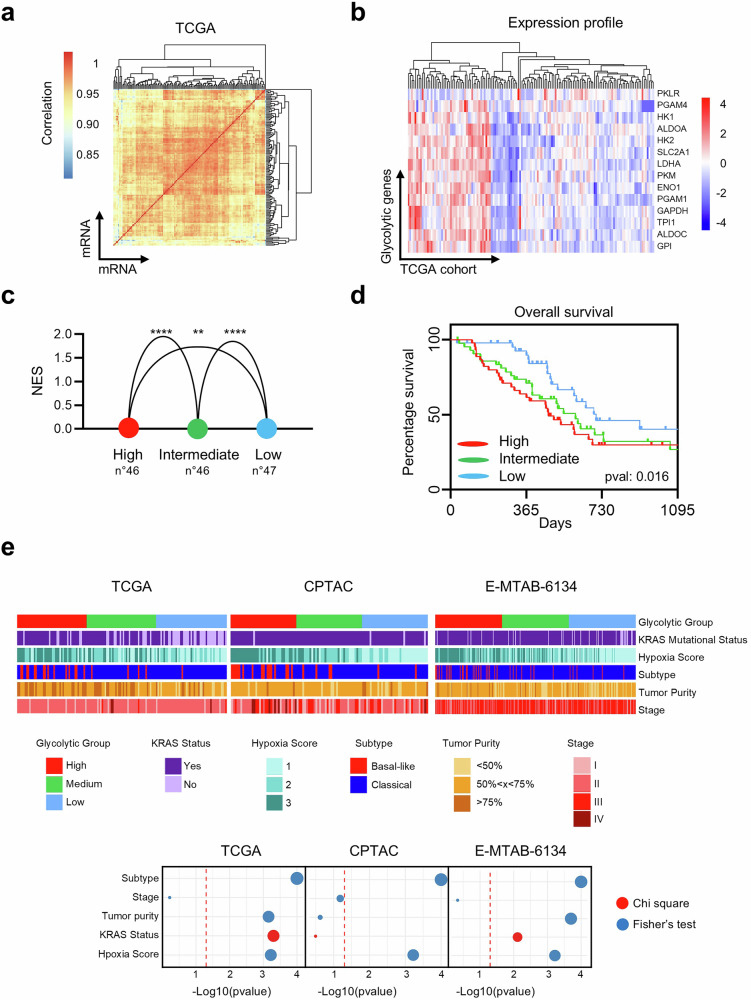
Glycolytic Signature Defines Prognostic Subgroups and Functional Associations in PDAC. **a** Pairwise correlation for gene expression in the TCGA cohort of PDAC patients. **b** Heatmap for unsupervised clustering analysis with glycolytic signature expression in the TCGA cohort of PDAC patients. **c** TCGA-PDAC cohort divided in tertile and grouped in High, Intermediate, and Low based on glycolytic signature expression (above). Glycolytic enrichment was compared between High vs Intermediate, and High vs Low group, and between Intermediate vs Lowest group. The *y*-axis represents the normalized enrichment score with statistical significance indicated by *p* value: <0.0001 ****, <0.001 ** (below). Data are represented as mean ± SD. **d** Kaplan–Meier plot showing 3-year overall survival across glycolytic groups in the TCGA cohort of PDAC patients. **e** Association and distribution of glycolytic groups with KRAS mutational status, hypoxia score, Basal-like/Classical molecular subtype, tumor purity, and tumor stage across the TCGA and independent validation cohorts of PDAC patients (top). Each dot represents the statistical significance of the association between the glycolytic group and the indicated clinical or molecular variable, evaluated using either Fisher’s exact test or the Chi-squared test (bottom). The red dashed line indicates the significance threshold (*p* = 0.05)

The newly identified clinical correlations of glycolytic gene expression in PDAC were further explored via transcriptomic data from The Cancer Genome Atlas (TCGA) (Fig. 3a). Unsupervised clustering based on the expression profiles of glycolytic genes revealed distinct expression groups (Fig. 3b). To investigate the functional and clinical relevance of this glycolytic heterogeneity, TCGA-PDAC patients were grouped into tertiles on the basis of their expression profiles (high, intermediate, and low), and GSEA was subsequently performed to compare glycolytic activity among the groups. Compared with the low group, the high-glycolytic group showed positive enrichment relative to the intermediate and low groups, and the intermediate group was enriched compared with the low group (Fig. 3c). These results support the presence of at least two distinct glycolytic phenotypes in PDAC, with one subgroup exhibiting a markedly elevated glycolytic profile. Leveraging this stratification, we assessed the clinical implications by analyzing 3-year survival rates among PDAC patients. A strong inverse relationship emerged between the glycolytic phenotype and survival, with the high-glycolytic group having the poorest prognosis (Fig. 3d). These associations were independently validated in separate patient cohorts, confirming the link between elevated glycolytic gene expression and adverse clinical outcomes (Supplementary Fig. 3a, b). Notably, these findings closely mirror those observed when patients were stratified by tumor stage (Supplementary Fig. 4a). Interestingly, metabolic stratification consistently revealed distinct survival trends across all datasets, aligning closely with those observed when stratifying by tumor stage. While the degree of statistical significance varied between cohorts, likely reflecting differences in cohort composition and stage distribution, the overall patterns observed support the notion that metabolic profiling captures clinically meaningful tumor heterogeneity. To further examine the influence of tumor hypoxia, we generated a hypoxia signature based on genes upregulated in PDAC compared with normal pancreatic tissue and regulated by hypoxia-inducible factor 1-alpha (HIF-1α). Hypoxia scores were calculated for both the TCGA and validation cohorts and confirmed via an established hypoxia signature. These scores progressively increased across groups with higher glycolytic expression, indicating a positive correlation between hypoxic status and glycolytic gene expression (Supplementary Fig. 4b). To determine whether metabolic expression patterns were associated with established PDAC transcriptional programs, the samples were grouped as basal-like or classical according to their gene expression signatures (Supplementary Fig. 4c). Additional variables analyzed included hypoxia score, KRAS mutation status, tumor purity, molecular subtype, and tumor stage. Notably, the glycolytic group showed consistent and significant associations with both hypoxia status and the basal-like subtype across cohorts (Fig. 3e). In contrast, no significant association was observed with tumor stage. Tumor purity and KRAS mutation status were significantly associated with the glycolytic group in two out of three cohorts, indicating partial cohort-dependent relationships. Additionally, the deconvoluted expression data revealed associations with the immune composition; specifically, a positive correlation between glycolytic groups and hypoxia conditions with M0 macrophages was observed (Supplementary Fig. 5a). Collectively, these findings demonstrate a strong association between hypoxia, enhanced glycolytic activity, and poor clinical outcomes in PDAC, supporting the existence of a hypoxia-driven shift toward anaerobic metabolism in highly glycolytic tumors.

### High-glycolytic PDAC models respond to metabolic interference with decreased proliferation via LDHA inhibition

**Fig. 4:**
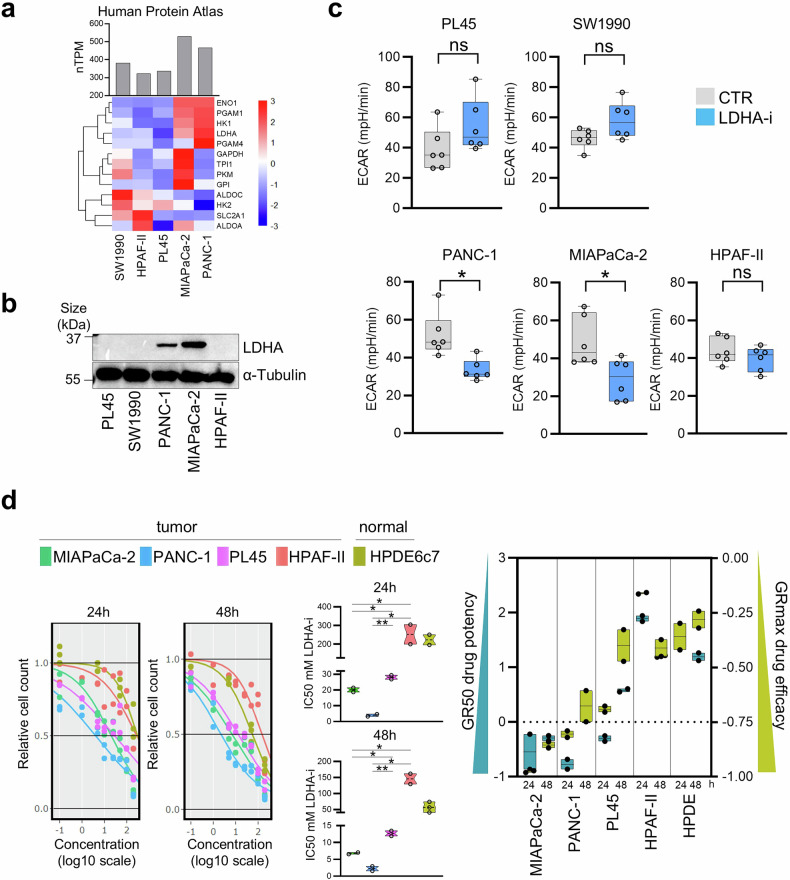
Glycolytic Profiling and LDHA Inhibition Reveal Differential Sensitivity Across PDAC Cell Lines. **a** Heatmap of glycolytic signature expression, reported as normalized transcript per million (nTPM) across five PDAC cell lines from Human Protein Atlas. **b** Western blot analysis of LDHA expression in PL45, SW1990, PANC-1, MIAPaCa-2, and HPAF-II cells. **c** Glycolytic activity in PL45, SW1990, PANC-1, MIAPaCa-2, and HPAF-II cells after LDHA-i treatment, measured as extracellular acidification rate (ECAR). Each dot represents a biological replicate (*n* = 6 per group). Data are represented as mean ± SD. Statistical analysis performed with a paired *t*-test. **d** Viability assay in MIAPaCa-2 (green), PANC-1 (blue), PL45 (purple), HPAF-II (red), and HPDE6c7 (brown) cells upon LDHA-i treatment at 24 and 48 h. Boxplots display IC50 values for each time point across cell lines, with statistical significance assessed by paired *t*-test (*n* = 2 per group). On the right, GR50 and GRmax values represent drug potency and efficacy, respectively

The functional significance of the glycolytic phenotype was substantiated via the use of PDAC cell models and transcriptomic profiles (Fig. 4a). A consistent pattern of glycolysis-related gene expression was observed across the PDAC cell lines, and dependency scores retrieved from public databases further confirmed distinct biological functions across these models (Supplementary Fig. 5b), enabling the identification of “high glycolytic” and “low glycolytic” profiles. These models were subsequently used to mirror the glycolytic heterogeneity identified in primary PDAC samples. Differences in terms of sensitivity, along with variations in heterogeneity between high and low systems following glycolysis disruption, were then investigated. LDHA expression, in addition to serving as the functional endpoint of glycolysis, was found to resemble glycolytic activity patterns across datasets, including both primary PDAC patient samples and PDAC cell lines (Supplementary Fig. 6a). This consistent correlation with the overall glycolytic profile highlights LDHA as a promising therapeutic target. After LDHA expression was validated at the protein level (Fig. 4b), the impact of LDHA inhibition on glycolytic activity was assessed by measuring the extracellular acidification rate (ECAR) in real time (Supplementary Fig. 6b). The results revealed a significant reduction in glycolysis in the high-glycolytic cell systems (MIAPaCa-2 and PANC-1), whereas the low-glycolytic systems (PL45, SW1990 and HPAF-II) presented a minimal response, as expected (Fig. 4c). The antiproliferative effects of oxamate-induced LDHA inhibition were evaluated by determining the IC50 values at 24 and 48 h in different pancreatic cell models. MIAPaCa-2 and PANC-1 cells were compared with PL45 and HPAF-II cells, along with normal pancreatic HPDE6c7 cells. Notably, highly glycolytic cell lines exhibited greater sensitivity to LDHA inhibition (Fig. 4d), highlighting the vulnerability of glycolytically active PDAC to metabolic disruption. In HPDE6c7 cells, treatment with the LDHA inhibitor had a negligible effect. This differential sensitivity was not observed with gemcitabine, a standard chemotherapeutic agent, which demonstrated more comparable efficacy across all cancer cell models (Supplementary Fig. 6c). To better assess the anticancer inhibitory effect of LDHA-i, tumor growth was also evaluated in 3D human pancreatic cancer organoids (Supplementary Fig. 7a). The results demonstrated that treatment inhibited organoid growth and reduced the organoid area in a dose-dependent manner, indicating that LDHA inhibition effectively targeted the clonogenic capacity of pancreatic cancer cells. Additionally, anticancer activity and toxicity were investigated with an in vivo chicken embryo model (Supplementary Fig. 7b). In terms of tumor growth, LDHA-i treatment resulted in significant regression of PDAC xenografts. With respect to toxicity, all the tested doses of LDHA-i were well tolerated by the embryos. Overall, PDAC cell line models recapitulate the glycolytic heterogeneity observed in primary PDAC samples, providing strong support for the existence of a therapeutic window in glycolytically active tumors. These results reinforce the potential utility of glycolytic stratification in PDAC to guide metabolic targeting strategies, particularly through LDHA inhibition. Furthermore, in vitro and in vivo complementary models provide physiologically and clinically relevant platforms to assess the safety and tolerability of metabolic intervention.

### LDHA inhibition substantially impacts intracellular metabolic flux, reverting high-glycolytic cell settings

**Fig. 5:**
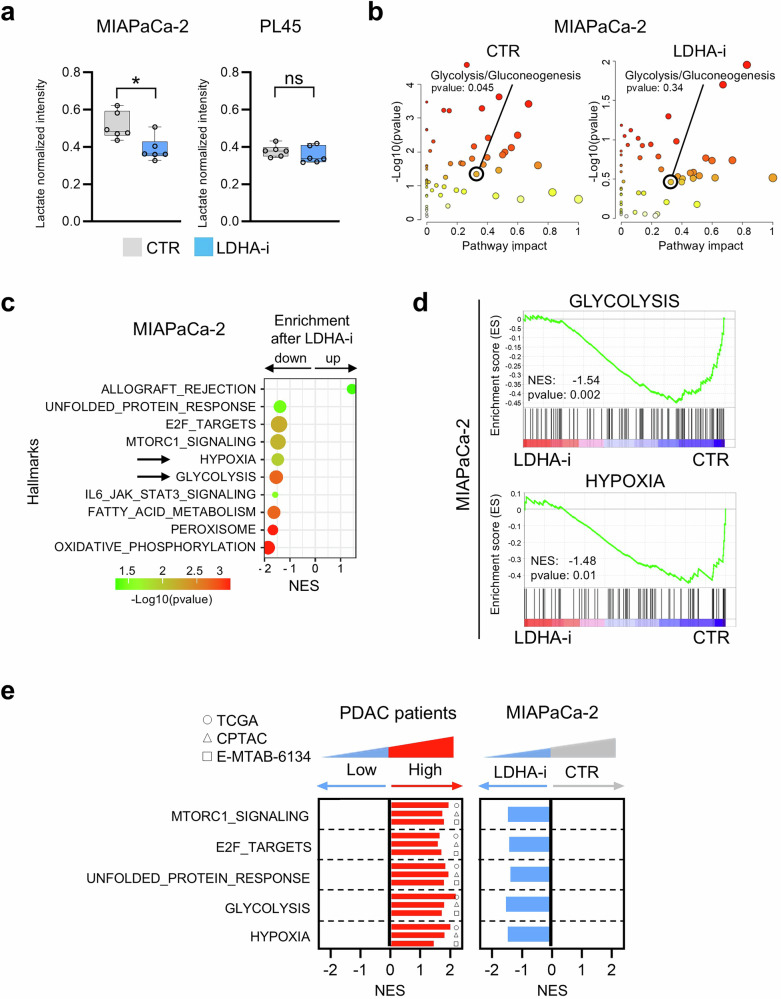
Metabolomic, Proteomic, and Transcriptomic Profiling Uncovers the Impact of LDHA Inhibition in PDAC. **a** Amount of lactate in MIAPaCa-2 and PL45 cells at the basal level (gray) and after LDHA inhibition (blue). Each dot represents a biological replicate (*n* = 6 per group). Data are shown as normalized intensity and are represented as mean ± SD. Statistical significance was calculated with a paired *t*-test. **b** Pathway enrichment analysis performed on metabolomics data in MIAPaCa-2 cells at basal level and after LDHA inhibition. The *x*-axis indicates pathway impact based on metabolite expression. The *y*-axis indicates *p* value transformed as −Log_10_. **c** Significant results from enrichment analysis performed on proteomics data in MIAPaCa-2 cells after LDHA inhibition. The *x*-axis indicates the normalized enrichment score. The label shows *p* value transformed as −Log_10_. **d** Enrichment plots for glycolysis and hypoxia in MIAPaCa-2 cells treated with LDHA-i. Normalized enrichment score and *p* value are reported in the figure. **e** Hallmarks significantly upregulated in PDAC patient samples exhibiting High glycolytic profile compared to those with Low glycolytic profile (left). Hallmarks significantly downregulated in MIAPaCa-2 cells following treatment with LDHA-i (right). The *y*-axis indicates the normalized enrichment score

The potential of glycolysis-based stratification of PDACs in response to metabolic inhibitors such as LDHA-i was further explored via a multiomics approach. Specifically, metabolomic, proteomic, and lipidomic analyses were performed in both high- and low-glycolytic cell systems upon treatment with LDHA-i (Supplementary Fig. 8a–c). Metabolomic analysis revealed a significant reduction in lactate levels in the high-glycolytic system following oxamate treatment, whereas no significant changes were observed in the low-glycolytic system (Fig. 5a). Indeed, LDHA-i reverted the lactate levels to those of the lower glycolytic cell systems, supporting its potential use in high-glycolytic settings. In MIAPaCa-2 high glycolytic cells, a distinct set of metabolites significantly contributed to the enrichment of glycolysis/gluconeogenesis pathways under basal conditions, whereas this enrichment was lost upon LDHA inhibition (Fig. 5b). No significant enrichment was observed in PL45 cells, the model for the low-glycolytic phenotype (Supplementary Fig. 9a), strongly supporting the ability of LDHA-i to reset the high-glycolytic phenotype to a less aggressive, low-glycolytic phenotype. These findings were validated in two additional cell lines representative of the respective glycolytic phenotypes (Supplementary Fig. 9b), confirming the robustness of the observed metabolic shifts and strongly supporting the ability of LDHA-i to reset the high-glycolytic phenotype to the less aggressive, low-glycolytic phenotype. Proteomic analysis highlighted metabolic differences, with enrichment analysis strengthening the differential glycolysis activity in the two systems at baseline (Supplementary Fig. 9c). When the effects of LDHA inhibition were compared, glycolysis was significantly reduced in the high-glycolytic MIAPaCa-2 system, along with reductions in other energy-related pathways, such as oxidative phosphorylation and fatty acid metabolism (Fig. 5c). In the low-glycolytic PL45 cell system, glycolysis was not significantly modulated, and oxidative phosphorylation and fatty acid metabolism were increased, suggesting the existence of a potential compensatory mechanism (Supplementary Fig. 10a). Notably, in MIAPaCa-2 cells, the reduction in glycolysis upon LDHA inhibition was also associated with a reduction in hypoxia (Fig. 5d), suggesting a tight link between these two features. Lipidomic analysis revealed further differences (Supplementary Fig. 10b). A significant reduction in triglyceride (TG) and ether-linked triglyceride (TG-O) species was observed in both cell lines following LDHA inhibition. Interestingly, PL45 cells presented greater reductions in these species, particularly the unsaturated species, and further differences in the degree of lipid saturation of ceramides were observed. Biological processes associated with PDAC patients with a high glycolytic profile modified by LDHA-i were then investigated. In addition to glycolysis, other processes, including hypoxia, the unfolded protein response (UPR), E2F targets, and mTORC1 signaling, which are highly expressed in highly glycolytic PDAC primary samples, were downregulated upon treatment in the MIAPaCa-2 cell model (Fig. 5e). Additionally, genes involved in the regulation of cell proliferation were highly expressed in primary samples with high glycolytic profiles but were downregulated in the cell model following LDHA-i treatment (Supplementary Fig. 10c). A comparison of our metabolomics data with published metabolomics datasets from PDAC patients revealed a panel of metabolites associated with lactate levels and the glycolytic profile (Supplementary Fig. 11a, b). L-Histidine, lysine, arginine, and 2′-deoxyguanosine were negatively correlated with lactate in PDAC primary samples and were present at greater levels in the low-glycolytic cell model with low lactate levels. In contrast, L-citrulline, L-carnitine, hypoxanthine, nicotinamide, and tiglylcarnitine were positively correlated with lactate in patient-derived samples and exhibited greater levels in the high-lactate cell model. Together, these findings corroborate the presence of glycolytic heterogeneity in PDAC, highlighting a different impact in response to glycolytic inhibition. In general, our data suggest that LDHA inhibition may convert a high glycolytic profile into a low glycolytic profile, which is clinically associated with a better prognosis and response to treatment.

### Divergent metabolic and stress response adaptations to LDHA inhibition in PDAC models

A multilayered analysis encompassing epithelial‒mesenchymal transition (EMT), cellular behavior, metabolic reprogramming, and stress-related signaling pathways was conducted to characterize and identify potential mechanisms of adaptation or vulnerability. Enrichment analyses revealed a trend toward EMT suppression, although this reduction did not reach statistical significance (Supplementary Fig. 12a). These results were supported by colony formation and transwell invasion assays, which demonstrated a significant decrease in proliferative and invasive capacities (Supplementary Fig. 12b, c). To complement the primary molecular assessments with a functional readout that is more representative of neoplastic behavior, a live-cell imaging assay was performed to monitor cell motility over 24 h. As shown in Supplementary Videos 1 and 2, LDHA inhibition was associated with a notable reduction in cell motility relative to that of the untreated controls (Supplementary Fig. 12d). Furthermore, the integration of metabolomic and proteomic data following LDHA-i enabled a comprehensive analysis of key metabolic pathways, including the pentose phosphate pathway (PPP), oxidative phosphorylation (OXPHOS), and the tricarboxylic acid (TCA) cycle (Supplementary Fig. 13). In MIAPaCa-2, a notable reduction in PPP-related targets was observed, suggesting a slowdown of anabolic processes essential for biosynthesis and redox homeostasis. In contrast, the PL45 model exhibited only minimal alterations in PPP activity, indicating a greater ability to sustain this pathway, potentially through compensatory mechanisms or intrinsic metabolic flexibility. Analysis of OXPHOS and TCA cycle components revealed global downregulation in MIAPaCa-2 cells. Conversely, several mitochondrial targets were upregulated in PL45 cells, indicating an attempt to activate alternative oxidative pathways to compensate for impaired glycolytic flux. Despite these molecular changes, mitochondrial stress tests did not reveal increased cellular respiration in either model following LDHA inhibition (Supplementary Fig. 14a–c), implying that the observed transcriptional or proteomic alterations do not translate into enhanced mitochondrial function. To further dissect the relationship between LDHA activity and downstream stress responses, we analyzed protein markers associated with hypoxia signaling and the UPR (Supplementary Fig. 14d). In MIAPaCa-2 cells, LDHA-i treatment led to time-dependent downregulation of HIF-1α protein levels, accompanied by a progressive increase in the expression of TIAR1, a well-known ER stress and UPR marker. In contrast, PL45 cells displayed an opposing trend, with sustained upregulation of HIF-1α and minimal changes in TIAR1 expression, suggesting differential engagement of the UPR and hypoxia pathways in response to LDHA-i. Baseline redox profiling revealed a higher GSH/GSSG ratio in MIAPaCa-2 cells than in PL45 cells (4.03 vs. 2.57), indicative of a more reducing intracellular environment and stronger antioxidant defenses. Following LDHA-i, MIAPaCa-2 cells presented a marked decrease in the GSH/GSSG ratio (to 2.36; Δ = −1.67), which was consistent with redox imbalance and elevated oxidative stress. This effect likely stems from impaired NAD⁺ regeneration, which disrupts glycolysis and compromises antioxidant systems. Interestingly, the NADH/NAD⁺ ratio in MIAPaCa-2 increased only modestly (from 0.0245 to 0.0356), suggesting partial compensation via residual mitochondrial activity or alternative oxidation routes. Conversely, PL45 cells displayed a more substantial increase in the NADH/NAD⁺ ratio (from 0.0593 to 0.1752) but only a moderate decrease in the GSH/GSSG ratio (to 1.98; Δ = −0.59), indicating comparatively milder redox perturbation. Additionally, LDHA-i elicited opposite effects on SOD2 gene expression in the two models, supporting the notion of divergent redox regulation (Supplementary Fig. 14e). In summary, the two PDAC models demonstrate distinct metabolic adaptations to LDHA inhibition, involving energy and anabolic pathways, coupled with redox imbalance and limited compensatory responses.

### Integrative analysis reveals molecular networks not directly related to glycolysis modulated by LDHA-i in a high-glycolytic PDAC system

**Fig. 6:**
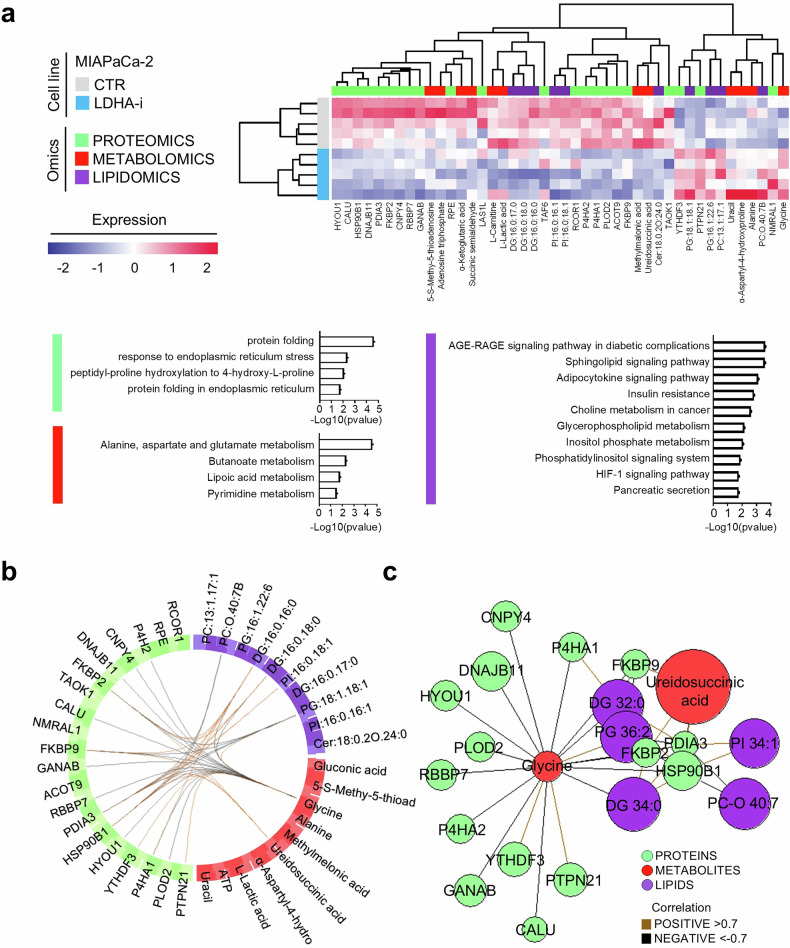
Integrative multiomics analysis correlates molecular signatures in PDAC. **a** Clustered image map of variables selected by multiblock partial least squares discriminant analysis (PLS-DA) based on omics datasets. Samples are displayed as rows, and selected features in columns. Histograms represent significant functional analysis results for proteins (light green), pathway analysis for metabolites (red), and enrichment analysis for lipids (purple). Statistical significance is expressed as −Log_10_ transformed *p* values. **b** Circos plot from multiblock PLS-DA performed on proteomic (green), metabolomic (red), and lipidomic (purple) data in MIAPaCa-2 cells treated with LDHA-i. The plot shows correlations between 0.7 and −0.7 across proteins, metabolites, and lipids, represented on the side quadrants. The internal connecting lines show positive (light brown) and negative (black) correlations. **c** Network plot of correlated features derived by multiblock PLS-DA. Each node represents a variable, color-coded by type. The color of the edges indicates positive (light brown) or negative (black) correlations greater than ±0.7 between variables of different types

An integrative approach combining metabolomics, proteomics, and lipidomics was employed to explore associations and correlations among metabolites, proteins, and lipids in MIAPaCa-2, a highly glycolytic PDAC system. This analysis identified distinct blocks of correlated features across the metabolite, protein, and lipid datasets, many of which were modulated by LDHA-i (Fig. 6a). At the proteomic level, processes related to protein folding and endoplasmic reticulum function were affected. The key modulated proteins included HYOU1, FKBP9, HSP90B1, DNAJB1, and PDIA3. Proline hydroxylation, involving P4HA1 and P4HA2, has also emerged as relevant. From a metabolic perspective, changes in α-ketoglutaric acid, alanine, N-carbonyl-aspartate, and succinic semialdehyde indicated disruptions in alanine, aspartate, and glutamate metabolism. Uracil levels indicate altered pyrimidine metabolism. Lipidomic analysis revealed a decrease in diacylglycerols (DGs) and ceramides, implicating pathways such as sphingolipid and phosphatidylinositol metabolism, in addition to HIF-1α signaling, insulin resistance, and diabetic complications. In contrast, increased phosphatidylcholine and phosphatidylinositol levels are linked to phosphatidylinositol signaling and choline metabolism in cancer. Cross-omics correlation analysis highlighted glycine and ureidosuccinic acid as key hubs (Fig. 6b). Glycine, which was upregulated after LDHA inhibition, was negatively correlated with folding-related proteins (HYOU1, FKBP9, HSP90B1, DNAJB1, and PDIA3) and various DGs (Fig. 6c). In contrast, ureidosuccinic acid, which was downregulated, was positively correlated with the same molecules. In summary, this integrative analysis revealed new molecular links and demonstrated that LDHA inhibition affects a wide range of cellular pathways in addition to glycolysis in PDAC models, which remains to be further investigated.

## Discussion

PDAC remains a major clinical challenge because of its aggressive biology, late diagnosis, and resistance to therapy. Here, we reveal previously underappreciated glycolytic heterogeneity within PDAC, identifying distinct metabolic phenotypes that correlate with disease progression and patient outcome. Stratification into high- and low-glycolytic states in PDAC not only reveals prognostic value but also highlights differential sensitivity to metabolic inhibitors such as LDHA inhibitors. Our preclinical models robustly represent the high-glycolytic PDAC subtype, although we recognize that they cannot fully encompass the extensive molecular and phenotypic heterogeneity of PDAC as a whole. However, the strength of our study lies in the consistent and reproducible findings across integrated multiomics and functional analyses. These results offer a solid foundation for future investigations, and we propose that the data presented here serve as a framework to guide further validation studies in more diverse patient-derived systems. Rather than limiting the impact of our findings, this highlights the importance of expanding the scope of metabolic profiling in PDAC to refine patient stratification and therapeutic targeting. Our findings offer a rationale for personalized metabolic therapies in patients with PDAC displaying high glycolytic phenotypes. Targeting LDHA with a metabolic inhibitor selectively reduced glycolytic activity in highly glycolytic PDAC models, which was associated with the poorest prognosis. In our study, LDHA expression served as a surrogate for glycolytic activity, suggesting its utility as a metabolic/patient stratification biomarker. Notably, LDHA inhibition suppressed lactate production and depleted anaerobic metabolism exclusively in high-glycolytic tumors, indicating a potential therapeutic window for LDHA inhibitors in this subgroup. Our results are consistent with prior work demonstrating that distinct metabolic subtypes within PDAC not only predict patient survival but also exhibit differential drug sensitivities, suggesting that combining metabolic inhibitors with conventional therapies may improve outcomes.^5^ Notably, we also observed altered macrophage levels in high-glycolytic PDAC samples. This observation is further supported by other evidence correlating molecular subgroups of PDAC with distinct survival outcomes and immune suppression.^6^ These converging results corroborate the link between glycolysis and immune cell composition within the tumor microenvironment, reinforce the prognostic and immunological relevance of glycolytic stratification in PDAC, and further highlight the potential of integrating metabolic and immune profiling to inform therapeutic strategies. While glycolytic phenotypes do not correlate with specific mutations, tumor location, or diabetes, hypoxia has emerged as a key determinant. High-glycolytic PDACs presented increased expression of hypoxia-related genes, which is consistent with previous reports linking hypoxia to immunosuppression, therapeutic resistance, and tumor plasticity.^12^ Independent of the question of whether hypoxia, a highly glycolytic phenotype, and immunosuppression are the cause or consequence of each other, our data revealed a link between hypoxia, the UPR, and the glycolytic state—high-glycolytic tumors exhibited activation of UPR pathways, which were reduced following LDHA inhibition. These results align with the growing evidence implicating the UPR in PDAC progression and resistance^13,14^ and suggest that modulating glycolysis may attenuate ER stress and improve cellular homeostasis.^13^ Notably, one study^9^ showed that the inhibition of LDHA was associated with reduced glycolysis and decreased pyruvate, which contributed to the acetyl-CoA pool. In line with these findings, our model confirms a similar metabolic shift upon LDHA inhibition with reduced oxidative phosphorylation and the TCA cycle, highlighting a conserved mechanism across PDAC models. However, variations observed in other experimental systems, identified with different glycolytic metabolisms, underscore the importance of metabolic stratification in predicting therapeutic response. These findings support the concept that patient-specific metabolic phenotyping could guide more effective and tailored metabolic interventions, considering the heterogeneous metabolic dependencies within PDAC. Furthermore, recent integrative bioinformatics analyses of large PDAC cohorts have identified clinically relevant metabolic subgroups on the basis of differential expression of glycolytic and cholesterogenic genes.^4^ This study classified PDAC into four metabolic subtypes—quiescent, glycolytic, cholesterogenic, and mixed—with glycolytic tumors resulting in the poorest survival outcomes in both resectable and metastatic settings. Notably, glycolytic tumors presented lower expression of the mitochondrial pyruvate carriers MPC1 and MPC2 and were enriched for KRAS and MYC amplifications, underscoring a genetic‒metabolic link. These findings align with and further validate our metabolic stratification, highlighting that balancing glycolysis and cholesterol synthesis pathways may influence tumor aggressiveness and patient prognosis. The incorporation of such metabolic axes could refine patient stratification and identify novel therapeutic vulnerabilities that target both glycolytic and lipid metabolic pathways. These insights are corroborated by recent findings demonstrating a strong correspondence between transcriptomic-based molecular subtypes and metabolically defined profiles in PDAC.^7^ In that study, the integration of genomic and metabolomic data further validated the clinical relevance of metabolic stratification, particularly in identifying glycolytic tumors as the most aggressive subtype with actionable vulnerabilities. These results reinforce our approach and confirm the translational potential of classifying PDAC on the basis of glycolytic activity to guide therapeutic interventions. Our multiomics approach further revealed the downregulation of proline hydroxylation signaling, particularly involving P4HA1, a regulator that stabilizes HIF-1α by limiting its degradation.^15^ Conversely, HIF-1α is normally targeted for degradation under normoxia via proline hydroxylation,^16^ suggesting that LDHA inhibition may restore the hypoxic balance and reduce HIF-1α–mediated tumor adaptation. At the mechanistic level, extended analyses revealed divergent cellular responses to LDHA inhibition between cancer models, particularly in pathways related to hypoxia, ER stress, and redox homeostasis. These differences reflect distinct metabolic adaptation strategies and underscore the importance of the tumor metabolic context in shaping the therapeutic response. Notably, we did not observe compensatory activation of oxidative phosphorylation or evidence of EMT, suggesting that LDHA inhibition does not induce major escape mechanisms commonly associated with treatment resistance. Thus, supporting previous evidence that high LDHA activity promotes migration and invasion in PDAC,^17^ our findings highlight the therapeutic potential of targeting metabolic vulnerabilities within molecularly and metabolically stratified patient subgroups. Excitingly, the metabolites histidine and lysine were found to be associated with low glycolytic activity and with better prognosis in PDAC patients. A separate study revealed that these metabolites are reduced in patients with PDAC,^18^ suggesting that histidine and lysine levels decrease at disease onset and continue to decrease as the disease progresses. Arginine, whose role in deprivation therapy was previously described,^19^ and its metabolite, citrulline, were negatively and positively correlated with lactate, respectively. This finding suggests that arginine-based metabolism may be accelerated in highly glycolytic PDAC profiles and could also indicate that evaluating the arginine-to-citrulline ratio may serve as a complementary tool for stratifying patients and informing treatment choices targeting metabolism. These findings further support the functional impact of LDHA-i in highly glycolytic PDAC patients and indicate that its ability to reverse PDAC characteristics is associated with poor clinical outcomes. Importantly, therapeutic strategies targeting LDHA within the PDAC microenvironment, including the use of novel pan-LDH inhibitors, have shown promising antitumor effects in preclinical models, supporting the translational potential of our findings.^20^ Additionally, recent advances in the discovery of novel LDH inhibitors have provided further impetus for this therapeutic avenue. For example, a structure-based virtual screening study identified a pyridazine derivative, compound 18 (RS6212), as a potent and specific LDH inhibitor with micromolar anticancer activity across multiple cancer cell lines. This compound demonstrated synergy with complex I inhibition in suppressing tumor growth, underscoring the potential for combinatorial metabolic targeting strategies. These findings support continued investigations of LDHA inhibitors as promising agents for cancer therapy and reinforce the translational importance of targeting metabolic vulnerabilities such as LDHA in PDAC.^21^ Furthermore, oxamate, the lead compound we used as an LDHA inhibitor in our experimental setting, has been demonstrated to improve glycemic control and insulin sensitivity in diabetic db/db mice by inhibiting tissue lactate production, reducing systemic inflammation and proinflammatory cytokines such as TNF-α and IL-6, and improving pancreatic islet morphology.^22^ This finding is particularly relevant given the frequent co-occurrence of diabetes in PDAC patients and suggests that LDHA inhibition may exert dual beneficial effects by simultaneously targeting tumor glycolysis and systemic metabolic dysfunction. Such systemic metabolic improvements may also enhance treatment efficacy and patient outcomes, reinforcing the therapeutic potential of LDHA inhibitors beyond direct tumor targeting. While our study provides novel insights into the glycolytic phenotype of PDAC and potential therapeutic targeting of LDHA, several limitations must be acknowledged. First, a comprehensive assessment of the potential toxicity and off-target effects of LDHA inhibitor(s) has not been performed. Understanding the safety profile of these compounds is critical before considering their clinical translation, as their effects on organismal health and tolerability remain largely unexplored. In our study, we compared data from patients with PDAC with data from the normal immortalized pancreatic cell line HPDE6c7 and evaluated the toxicity and anticancer effects of oxamate in an embryo chicken model and patient-derived PDAC organoids. In vivo, treatment with oxamate led to a measurable reduction in tumor size compared with that of the controls, without affecting embryo viability, even at high doses. These complementary models provide an important bridge between in vitro assays and full animal studies, offering clinically and biologically relevant platforms for validating metabolic interventions. Importantly, they also help address potential concerns about the nonspecific toxicity and translational applicability of LDHA-targeting strategies. Second, our spatial transcriptomics cohort included only six patients, which represents a relatively small sample size. This limitation reduces the statistical power and may affect the generalizability of our findings across the broader PDAC patient population. Larger, independent cohorts will be necessary to validate and extend our observations. The integration of data from multiple independent studies and the ongoing back-and-forth relationships between identified signatures and their reproducibility in in vitro models can only partially address this limitation. Importantly, pancreatic cancer is not always operable, and even when surgery is possible, obtaining a sufficiently large tissue sample for research purposes is often challenging. Therefore, the inclusion of only six patients can help build a more robust framework for future investigations. Third, the technical aspects of our study introduce inherent challenges. Batch effects and other experimental biases are known issues when integrating data from multiple sources and platforms, potentially influencing data interpretation and reproducibility. Although we implemented strategies to mitigate these factors, residual confounding cannot be fully excluded. Moreover, the complex nature of multiomics data analysis demands cautious interpretation, and further refinement of analytical approaches is warranted. From a clinical perspective, the integration of technologies such as spatial transcriptomics into routine practice presents significant challenges, including high costs, limited availability, and the need for specialized infrastructure and expertise. Additionally, the clinical utility of LDHA inhibition remains speculative at this stage, and validated biomarkers to guide patient stratification or therapeutic response are lacking. Before such strategies can be translated into clinical use, further preclinical development, toxicological assessment, and, ultimately, clinical trials are needed.

In conclusion, our study reveals that glycolytic heterogeneity is a previously unrecognized stratification axis in PDAC and highlights the distinct metabolic vulnerability of its most aggressive forms. By linking high glycolytic activity to specific molecular features and therapeutic sensitivity, we provide a strong rationale for incorporating metabolic profiling into precision treatment strategies, paving the way for targeted metabolic therapies in PDAC. These data integrate well with recent advances showing that metabolic subtype classification not only reflects tumor biology but also offers actionable targets for therapy, emphasizing the importance of combining metabolic inhibitors with established treatments to overcome the aggressive nature of PDAC.^7^

## Materials and methods

### Single-cell RNA sequencing analysis

#### Preprocessing and clustering methods

The scRNA-seq dataset used for downstream analysis included 24 PDAC and 11 normal pancreas samples from ref. ^23^ and 23 PDAC and 5 normal pancreas samples from ref. ^24^. Data analysis was performed via the Seurat R package (version 5.3.0).^25–29^ The CreateSeuratObject function was used to create a Seurat object starting from raw data and then subsetted with the following parameters: nFeature_RNA > 200; nFeature_RNA < 2500; percent.mt < 5. The normalization function with the “LogNormalize” method was used to normalize the matrix, and FindVariableFeatures with selection.method “vst” was used to define cell-to-cell variation. Next, a linear transformation (scaling) was performed as a standard preprocessing step prior to dimensional reduction techniques such as principal component analysis (PCA) via the ScaleData function. This was followed by a linear dimensional reduction step that, as described in the Seurat vignette, uses the RunPCA function with previously determined variable features or a custom subset of features as input. Two analysis methods were then performed in parallel via the RunPCA function, with (i) features = VariableFeatures and (ii) features = metabolism gene list from Reactome (https://reactome.org/), as shown in Supplementary Table 1. For both methods, the FindNeighbors and FindClusters functions were used to compute the k.param nearest neighbors and to identify clusters of cells via a shared nearest-neighbor modularity optimization-based clustering algorithm. Finally, the RunUMAP function was used to build a Seurat object containing a UMAP representation. To correct for batch effects and integrate single-cell datasets originating from different sources, the Harmony v1.2.3^30^ algorithm implemented in the Seurat workflow via the RunHarmony() function was used. Specifically, batch correction was performed using the dataset of origin as the grouping variable.

#### Canonical markers

The cell type was determined on the basis of the expression of known markers as described in refs. ^23,31,32^: COL1A1, LUM, DCN (fibroblasts); ACTA2, RGS5, ADIRF, PDGFRB (stellate cells); CHGA, CHGB (endocrine cells); REG1B, PRSS1, CTRB1 (acinar cells); AMBP, CFTR (normal ductal cells); KRT7, KRT19, TSPAN8, SLPI5 (tumor ductal cells); MS4A1, CD79A, CD79B (B cells); CD3D, CD3E, CD3G, CD8A (T cells); CD14, AIF1, MS4A7 (macrophages); and CDH5, CLDN5, PLVAP (endothelial cells).

#### Gene ontology analysis

GO analysis for biological processes was performed via DAVID (https://david.ncifcrf.gov/), an online tool for functional annotation, which uses a gene list from tumor cells defined with the FindMarkers function.

#### Per-sample centroid calculation and permutation testing

To assess the intersample heterogeneity of tumor cells, UMAP coordinates for each cell were extracted and grouped by embedded tumor ductal cells. Centroids were calculated as the mean UMAP coordinates per sample. Pairwise Euclidean distances between centroids were computed, and the mean intercentroid distance was taken as a measure of heterogeneity. To evaluate statistical significance, we performed a permutation test (*n* = 1000). Sample labels were randomly shuffled across cells, centroids were recalculated, and mean centroid distances were computed for each permutation. The empirical *p* value was defined as the fraction of permuted datasets with a mean centroid distance ≥ observed.

#### Differential expression analysis between the high- and low-glycolysis groups and GO analysis

Differential expression analysis between the high- and low-glycolysis patient groups was performed via the Seurat framework (v4.4.0). The tumor ductal cells were subsets from the integrated Seurat object. Patients were assigned to high- or low-glycolysis groups on the basis of the median of their mean log₂-normalized expression of glycolytic genes. The analysis was performed at the single-cell level, and all tumor cells from high-glycolysis patients were compared with those from low-glycolysis patients. Genes with an adjusted *p* value (Benjamini–Hochberg correction) < 0.05 were considered significantly differentially expressed. These upregulated gene lists were then used for GO analysis for pathways and biological processes, and analysis was carried out via the enrichR v3.4 R package^33^ for functional annotation.

#### Gene set enrichment analysis

Glycolytic enrichment analysis was performed with GSEA (version 4.3.1) via the hallmark gene set database to investigate differences across the high- and low-glycolytic groups in single-cell ductal tumor cells.

### Spatial transcriptomic analysis

Pancreas samples were acquired from patients with a PDAC diagnosis, with a grade between 2–3, T and N, respectively, between 2–3 and 0–1. The clinical information of the PDAC patients is provided in Supplementary Table 2.

#### Sequencing

As described in ref. ^34^, RNA in situ hybridization was used to localize mRNA within tissue samples. The GeoMx RNA assay was performed for multiplexed detection of mRNA targets in formalin-fixed, paraffin-embedded (FFPE) tissues. Briefly, 5 μm FFPE sections were dewaxed, digested with proteinase K, fixed, and incubated overnight with RNA detection probes linked to indexing oligo barcodes via a photocleavable linker. After washing, fluorescently labeled antibodies were added to serve as morphology markers. Regions of interest (ROIs) were then defined: PanCK+ staining was used to segment ductal cells, PanCK− staining was used for acinar cells, and Ins+ and Gcg+ staining was used for β- and α-cells, respectively. ROIs were manually drawn around specific structures in each stained tissue core. The samples were subsequently processed and profiled on a GeoMx Digital Spatial Profiler (NanoString Technologies, Seattle, WA, USA) through the Technology Access Program following the manufacturer’s instructions. The RNA targets were analyzed via region-specific UV photocleavage, and the indexing oligos were collected for downstream quantification. Cleaved indices were then quantified via NanoString nCounter Technology, resulting in the digital quantification of RNA expression in a spatial context. Quality control (QC) and data normalization were carried out following the standard workflow recommended by NanoString for GeoMx analysis. ROIs were filtered on the basis of the following QC criteria: more than 1000 reads per ROI; more than 80% of reads successfully trimmed, stitched, and aligned; sequencing saturation greater than 50%; minimum negative control counts above 5; no-template control counts less than 3000; more than 100 nuclei per ROI; and an ROI area larger than 1000 μm^2^.

#### Deconvolution analysis

Spatial transcriptomics data were deconvoluted for TME estimation via the SpatialDecon R package (version 1.12.3), a computational tool for spatial and/or bulk gene expression data.^35^ Briefly, normalized counts and the raw matrix from spatial transcriptomics were analyzed via the TME reference as a preloaded gene list on pure tumor segments, and nuclei counts were used to estimate total cells. All the cell types identified in the tumor regions are provided in Supplementary Table 3.

#### Clustering modules on spatial transcriptomics

The unsupervised classification technique was used to reveal trends hidden in large datasets with the Mfuzz R package (version 2.64.0),^36^ which uses gene expression as input and then exports the gene list from the cluster of interest.

#### Gene ontology analysis

GO analysis for biological processes was performed with DAVID, using the gene list from tumor cells.

### Correlations in public databases

#### Data acquisition and preprocessing

TCGA cohort was selected for correlation analyses because of its large patient sample size and the availability of comprehensive clinical data, including mutational profiles and survival information. To validate our findings, two independent validation cohorts, the Clinical Proteomic Tumor Analysis Consortium (CPTAC) and E-MTAB-6134, which were chosen on the basis of similar criteria—substantial patient numbers and publicly accessible clinical and molecular data—were used to ensure the robustness and generalizability of the results. The transcriptomic expression, mutational profile, and survival data of PDAC patients were downloaded from the TCGA (https://www.cancer.gov/), CPTAC (https://portal.gdc.cancer.gov/), and OmicsDI (https://www.omicsdi.org/) databases. For TCGA and CPTAC, raw RNA-seq count data and clinical metadata were imported into the R environment via TCGAbiolinks v2.32.00. E-MTAB-6134 array data^37^ were retrieved directly from BioStudies (https://www.ebi.ac.uk/biostudies/arrayexpress/studies/E-MTAB-6134).

#### Data filtering

For exploratory analysis, the TCGA-PDAC cohort (*n* = 185) was filtered by diagnosis according to Peran et al.^38^, resulting in a cohort of 144 patients. Patients whose KRAS mutation status was unavailable were subsequently excluded, yielding a final cohort of 139 patients. The CPTAC-PDAC cohort (*n* = 179) was filtered by diagnosis, excluding patients whose KRAS mutation status was unavailable, resulting in a cohort of 105 patients. The E-MTAB-6134 cohort (*n* = 309) was filtered by diagnosis, excluding patients whose KRAS mutation status was unavailable, resulting in a cohort of 261 patients. The list of filtered datasets used for downstream analyses in this study is available in Supplementary Table 4.

#### Differential expression analysis, normalization, score calculation, and group stratification

For the TCGA and CPTAC cohorts, differential expression analysis was performed via the DESeq2 v1.44.00 package.^39^ Normalization was performed via estimation of size factors to account for differences in sequencing depth. Variance-stabilizing transformation (VST) was applied to normalized counts to stabilize variance across the mean. Pairwise Pearson correlation coefficients were computed from the VST-transformed expression matrix to visualize with a heatmap of pairwise correlation as a hierarchical clustering method. For E-MTAB-6134, CEL files were unzipped and imported into R via the affy v1.82.00 package.^40^ The raw intensity values were processed via robust multiarray average normalization. Probe-level expression data were mapped to gene symbols via the hgu219.db v3.2.3 annotation package. For group stratification, a subset of genes related to the glycolytic signature (HK1, HK2, PGAM4, LDHA, SLC2A1, PKM2, ALDOA, ENO1, ALDOC, GPI, PGAM1, GAPDH, TPI1, and PKLR) was selected to assess expression patterns across samples in each cohort. The score was defined as the average of the normalized expression values of the genes in the glycolytic signature and was calculated for each patient. Patients were stratified into three groups (low, medium, and high) on the basis of tertiles of the expression score distribution.

#### Gene set enrichment analysis

Glycolytic enrichment analysis was performed with GSEA (version 4.3.1)^41^ via the hallmark gene set database to investigate differences across three groups (low, medium, and high) of PDAC patients identified in the TCGA, CPTAC, and E-MTAB cohorts uploaded as normalized counts. Parameters used for analysis: nperm 1000 and phenotype permutation.

#### Survival analysis

The associations between glycolytic groups and overall survival were calculated for the three expression tertiles (low, medium, high) of PDAC patients identified in the TCGA, CPTAC, and E-MTAB cohorts via the survminer v0.5.0 package. Statistical significance was assessed via the log-rank test, and Cox proportional hazards models were used to test for trends in survival across groups.

#### Hypoxic score signature

The PDAC hypoxia signature was calculated in each glycolytic group as the median of overlapping genes upregulated in PDAC compared with normal tissue and transcriptionally regulated by HIF-1α (Supplementary Table 5). Differentially expressed genes in PDAC and normal pancreas samples were identified via GEPIA,^42^ an online tool that assesses gene expression in cancer types and paired *normal* samples. Gene targets of HIF-1α were identified via TRRUST (https://www.grnpedia.org/trrust), an online database of human transcriptional regulatory networks. A published hypoxia signature from ref. ^43^ was used for signature validation. Statistical significance was calculated via ANOVA.

#### Inference of basic-like and classical PDAC subtypes from gene expression signatures

Basal-like and classical PDAC subtypes were inferred within the glycolytic groups to define molecular associations. Gene expression signatures for each subtype were retrieved from Moffitt et al.^44^. For each PDAC cohort (TCGA, CPTAC, and E-MTAB-6134), the mean expression value of each signature was computed per sample from normalized gene expression matrices. The samples were subsequently classified as basal-like or classical, depending on which signature presented the highest mean expression score.

#### Tumor purity estimation

Tumor purity was estimated via the tidyestimate (v1.1.1) R package. For each PDAC cohort, the normalized gene expression matrix was filtered to retain only genes overlapping with the ESTIMATE reference gene set. On the basis of the resulting tumor purity percentages, the samples were categorized into three groups: <50%, 50–75%, and >75% purity.

#### Statistical associations between glycolytic status and clinical/molecular variables

Associations between glycolytic status and selected clinical or molecular variables were assessed in RStudio. Categorical variables—including hypoxia score, KRAS mutation status, tumor purity, subtype, and stage—were tested for associations with the glycolytic group. For each comparison, both Chi-square and Fisher’s exact tests were performed as appropriate. *P* values were calculated for each variable, rounded to four decimal places, and ranked by significance. Associations with *p* < 0.05 were considered statistically significant.

### PDAC cell line dataset sources

The dataset analyzed in this study was obtained from the Human Protein Atlas (https://www.proteinatlas.org/) and contains transcriptomic profiles across various human cell lines. Normalized transcripts per million (nTPM) values were imported into R, where a subset of pancreatic cancer cell lines—PANC-1, PL45, HPAF-II, SW1990, and MIAPaCa-2—was selected for analysis. To investigate glycolytic heterogeneity, the expression of a predefined glycolytic gene signature (HK1, HK2, LDHA, SLC2A1, PKM, ALDOA, ENO1, ALDOC, GPI, PGAM1, GAPDH, TPI1, PGAM4, and PKLR), as well as an overall glycolytic score, was assessed across the selected cell models.

### Gene dependency analysis

To evaluate the functional relevance of key glycolytic genes in pancreatic cancer cell lines, CRISPR‒Cas9 gene dependency data were obtained from the DepMap project (Broad Institute) via the depmap R package (v1.18.0). Gene dependency scores (CERES scores) were extracted via the depmap_crispr() function. Pancreatic cancer cell lines were filtered from the gene effect matrix, and dependency scores were retrieved for a predefined set of glycolysis-related genes: HK1, HK2, LDHA, SLC2A1, PKM, ALDOA, ENO1, ALDOC, GPI, PGAM1, GAPDH, TPI1, PGAM4, and PKLR. An aggregate glycolysis dependency score was calculated for each cell line by averaging the CERES values across the entire gene set. Data visualization was performed via scatter plots to assess overall sensitivity to glycolytic pathway inhibition. In parallel, the GeMap database (https://depmap.org/portal/gemap/), a curated resource that consolidates genetic dependency data across multiple platforms and tissue types, was queried. The same glycolytic genes were analyzed within matched PDAC cell lines to compare dependency patterns and confirm consistency with the DepMap-derived results. This complementary analysis further supported the importance of selected glycolytic enzymes in pancreatic cancer models.

### Cell lines

The PL45 (CRL-2558),^45^ SW1990 (CRL-2172),^46^ PANC-1 (CRL-1469),^47^ HPAF-II (CRL-1997),^48^ and MIAPaCa-2 (CRL-1420)^49^ cell lines were purchased from ATCC. The cell lines were cultured in DMEM (Euroclone, #ECB7501L) supplemented with 10% heat-inactivated fetal bovine serum (Sigma‒Aldrich, #F7524), 250 ng/mL amphotericin B (Euroclone, #ECM0009D), 2 mM L-glutamine (Euroclone, #ECB3000D), and penicillin–streptomycin mixture at 100 U/mL and 100 μg/mL (Euroclone, #ECB3001D). The incubator conditions for cell culture were 37 °C and 5% CO_2_. An EZ‒PCR Mycoplasma Test Kit (Biological Industries, #20–700-20) was used to test the cells for mycoplasma contamination. HPDE6c7 cells were maintained in Advanced DMEM F12 (Gibco, #12634-010), B27 17504044 (Gibco, #17504044), N2 (Gibco, #17502-048), bFGF (PEPROTECH, #AF-100-18b-250UG), EGF (PEPROTECH, #AF-100-15-500UG), and HEPES (Euroclone, # ECM0180D). Glutamax (Gibco, #35050-038).

### Chemicals

Oxamate (S6871) and gemcitabine (S1714) were purchased from Selleck Chemicals and dissolved according to the manufacturer’s instructions.

### Western blot analysis

The extraction of cell pellets was carried out as reported previously.^50^ The primary antibodies used were as follows: tubulin (#sc-5286) from Santa Cruz Biotechnology, LDHA (C4B5, #3582) from Cell Signaling Technology, TIAR (#610352) from BD Biosciences, HIF-1α (#ab16066) from Abcam, G3BP1 (#A302-033A) from BETHYL, vinculin (#66305-1) and β-actin (#66009-1) from Proteintech. Antibodies were used according to the manufacturer’s instructions. Image acquisition was performed via a ChemiDoc™ MP Imaging System (Bio-Rad, #12003154).

### Cell viability assay

The viability of the MIAPaCa-2, PL45, PANC-1, HPAF-II, and HPDE6c7 cell lines was assessed via the use of thiazolyl blue tetrazolium bromide (MTT; Sigma-Aldrich, #57,360-69-7) according to the manufacturer’s instructions. A total of 3 × 10^3^ cells/well were seeded in a 96-well plate. The following day, the cells were treated with oxamate and gemcitabine. Oxamate was used at final concentrations of 0.1, 1, 5, 10, 25, 50, 100, and 200 mM for 24 h and 48 h. Gemcitabine was used at final concentrations of 0.001, 0.01, 0.1, 0.2, 0.5, 1, 5, and 10 μM for 24 h and 48 h. Absorbance values were measured at a wavelength of 570 nm via an Infinite M-plex instrument (Tecan, #30190085). Statistical analysis and IC_50_ calculations were performed via the GRmetrics R package (version 1.30.0).^51^

### Seahorse assays

A Glycolytic Stress Test Kit (Agilent Technologies, #103017-100) was used in PL45, SW1990, PANC-1, HPAF-II, and MIAPaCa-2 cells, and a Mito Stress Test Kit (Agilent Technologies, #103015–100) was used in PL45, PANC-1, HPAF-II, and MIAPaCa-2 cells with a Seahorse XF96 Analyzer (Agilent Technologies). Briefly, 8 × 10^3^ cells were plated 24 h prior to analysis and then treated with 5 mM oxamate for 3 h. The steps for incubation, medium replacement, loading into the XFe96 Analyzer, and injection were performed according to the manufacturer’s instructions. Seahorse Wave software (version 2.2.0) (Agilent Technologies) was used to analyze the data. The experiments were conducted in sextuplicate. The paired *t*-test was used to determine statistical significance, which was then expressed as a *p* value. The error bars display the standard deviations.

### Metabolic phenotypes

The energy metabolism of PL45, PANC-1, HPAF-II, and MIAPaCa-2 cells was analyzed through simultaneous measurements of the ECAR and oxygen consumption rate via a Seahorse XF metabolic analyzer (Agilent Technologies). Measurements were performed under basal (baseline) conditions and following metabolic stress (stressed) to evaluate the metabolic flexibility of the cells.

### Colony formation assay

MIAPaCa-2 cells were treated with 5 mM oxamate for 96 h. Following treatment, the medium was replaced with drug-free medium, and the cells were cultured for an additional 7 days. Colonies were then stained with crystal violet, and the colony count, total area, and average size were quantified via ImageJ software. The dye was subsequently dissolved, and the absorbance was measured at 592 nm via an Infinite M1000 microplate reader (Tecan) to quantify relative colony formation.

### Transwell assay

MIAPaCa-2 cells (1 × 10^5^ cells/well) were seeded and pretreated with 5 mM oxamate for 24 h. Subsequently, 5 × 10^4^ cells were counted and seeded into the upper chambers of transwell plates in serum-free medium with membrane inserts. Moreover, 0.6 ml of DMEM supplemented with 10% FBS was added to the lower chamber as a chemoattractant. The plates were incubated at 37 °C for 16 h. The cells that migrated to the lower surface of the membranes were fixed, stained with crystal violet, and imaged at 20× magnification. Migration metrics—including cell counts, total area, and average size—were quantified via ImageJ software.

### Live-cell imaging

MIAPaCa-2 cells were seeded in glass-bottom 96-well plates (PhenoPlate, PerkinElmer) at a density of 2 × 10^3^ cells per well. After 24 h, the cells were treated with 5 mM oxamate. Time-lapse brightfield imaging was performed for 24 h using an EVOS M7000 microscope (Thermo Fisher Scientific). Live-cell images for the MIAPaCa-2 control and treated groups are available in Supplementary Videos 1 and 2, respectively. Time-lapse images were processed via flat-field correction and Gaussian smoothing (1 px). Cell motility was quantified by manual tracking via the Manual Tracking plugin in FIJI (ImageJ).

### Maintenance, drug exposure, and growth assessment of human PDAC organoids

Human PDAC organoids (HCM-CSHL-0092-C25, PDM-39, ATCC) were maintained in Organoid Media Formulation #3 (ATCC, ACS-7101), which was prepared according to the manufacturer’s instructions. In brief, the complete growth medium consisted of Advanced DMEM/F12 (Thermo Fisher Scientific, 12634028) supplemented with 10 mM HEPES (Thermo Fisher Scientific, 15630080), 2 mM L-glutamine (ATCC, 30-2214), 1× B-27 supplement (Thermo Fisher Scientific, 17504-044), and the following recombinant factors from the Organoid Growth Kit: Noggin (ATCC, ACS-7200), EGF (ATCC, ACS-7202), nicotinamide (ATCC, ACS-7214), N-acetylcysteine (ATCC, ACS-7215), FGF-10 (ATCC, ACS-7204), Gastrin (ATCC, ACS-7208), and A83-01 (ATCC, ACS-7209). Conditioned media from HA-R-Spondin1-Fc 293T cells (Trevigen, 3710-001-01) and L-Wnt-3A cells (ATCC, CRL-2647) were added at final concentrations of 10% and 50%, respectively. The complete medium was sterile-filtered via a 0.22 µm PES bottle-top filter and used within 4 weeks of preparation. For the experiments, single-cell suspensions were obtained from cultured organoids. For each condition, 1000 cells were resuspended in 10 µL of basement membrane extract (BME2, R&D Systems, #3533-005-02) and plated as individual droplets in triplicate wells of a 96-well plate (Corning, #3596). The plate was then inverted and incubated at 37 °C for 15 min to allow the BME to polymerize before being overlaid with culture medium. Twenty-four hours post-plating (T1), the organoids were divided into three treatment groups: vehicle control (water), 5 mM oxamate, and 25 mM oxamate. The treatments were changed at 72 h (T3) and 120 h (T5) by replacing the culture medium with fresh medium containing the corresponding drug or vehicle concentration. After 7 days (T7) of culture, the organoids were imaged via an EVOS M5000 imaging system (Thermo Fisher Scientific) with a 40× objective. The total number of organoids per well was counted manually. The cross-sectional area of individual organoids was measured from the acquired images via ImageJ software (National Institutes of Health). Data from triplicate wells were pooled for analysis.

### In vivo efficacy and toxicity of oxamate in the CAM assay

The in vivo efficacy and toxicity of oxamate, evaluated at 10–45–90 mM in tumors derived from the PDAC cell line MIAPaCa-2, were assessed in a chick embryo chorioallantoic membrane (CAM) study conducted by Innovotion (La Tronche/Grenoble, France). The vehicle was included as a negative control. The fertilized White Leghorn eggs were incubated at 37.5 °C with 50% relative humidity for 9 days. On embryonic day 9 (E9), the CAM was exposed by drilling a small hole into the air sac and creating a 1 cm^2^ window in the eggshell. For each experimental group, at least 20 eggs were grafted; however, depending on embryo survival following tumor implantation, the actual number per group ranged from 15 to 20. On E9, the cells were detached with trypsin, washed with complete medium, and resuspended in graft medium. A total of 1 × 10^6^ cells were applied to the CAM of each egg, which were subsequently randomized into experimental groups. For tumor harvesting on E18, tumors along with the upper portion of the CAM were excised, washed with phosphate-buffered saline, and fixed in paraformaldehyde for 48 h. The tumors were then carefully separated from normal CAM tissue and weighed. Embryonic viability was monitored daily, and any deaths or visible gross abnormalities were recorded to assess treatment-induced toxicity. Final mortality rates were determined, and Kaplan–Meier survival curves were generated for all groups. Tumor weight and other quantitative data were analyzed via one-way ANOVA followed by appropriate post hoc tests.

### Immunohistochemistry

Clinical and pathological data from six PDAC patients were retrieved from medical records, and histological parameters were assessed via hematoxylin and eosin-stained sections. Immunohistochemical staining was performed on 3–4 μm FFPE tissue sections via automated platforms (BenchMark ULTRA, Ventana/Roche Diagnostics; BOND-MAX, Leica Biosystems) following the manufacturers’ protocols. Positive and negative controls were included in each staining run. Staining was independently evaluated in both tumor and adjacent normal tissues. The antibodies used were as follows: CD4, CD8, CD163, CD68, and Granzyme B from Roche Diagnostics; CD103, FOXP3, and CD56 from Leica Biosystems; and LDHA from Cell Signaling Technology, which was used according to the suppliers’ recommendations.

### Omics in PDAC cell lines

#### Proteome extraction and analysis

The cell pellets were digested and purified with a PreOmics iST kit (PreOmics) according to the manufacturer’s instructions. Proteomic analysis was performed as previously reported^52^ via nanoliquid chromatography-high-resolution mass spectrometry via an Ultimate 3000 nanoLC system (Thermo Fisher Scientific) coupled to an Orbitrap Lumos Tribrid mass spectrometer (Thermo Fisher Scientific) with an EASY-Spray nanoelectrospray ion source (Thermo Fisher Scientific).

#### Preprocessing for proteomics

Peptides were trapped for 1 min in a PepMap Trap-Cartridge, 100 Å, 5 µm, 0.3 × 5 mm (Thermo Fisher Scientific) and separated on a C18 reversed-phase column (250 mm × 75 μm I.D., 2.0 µm, 100 Å; Thermo Fisher Scientific). The mobile phases were as follows: (A) 0.1% HCOOH in water (v/v) and (B) 0.1% HCOOH in ACN/water (v/v 80/20). Peptides were separated via a linear gradient of 90 min. HRMS analysis was performed via data-dependent acquisition, with an MS1 range of 400–1500 *m*/*z*; HCD fragmentation was used with a normalized collision energy setting of 27. The resolution was set at 120,000 for MS1 and 15,000 for MS/MS. Single charge peptides and unassigned charge peptides were excluded. The quadrupole isolation was set to 3 Da. The maximum ion injection times for the MS (OT) and MS/MS (OT) scans were set to auto and 50 ms, respectively, and the ACG values were set to standard values. Dynamic exclusion: For data processing, raw MS data were analyzed via Proteome Discoverer (version 2.5) (Thermo Fisher Scientific). The following parameters were used: enzyme trypsin, missed cleavages max 1, mass accuracy tolerance 10 ppm, and 0.6 Da for precursors and fragments, respectively. The search and Percolator algorithms were subsequently used. Carbamidomethylcysteine was used as a fixed modification, and methionine oxidation was used as a variable. Proteins with at least one unique peptide were identified via false discovery rate (FDR) thresholds of 0.01% (strict) and 1% (relaxed). Each analysis was performed in triplicate. For data processing, raw MS data were analyzed via Proteome Discoverer (version 2.5) (Thermo Fisher Scientific). The following parameters were used: enzyme trypsin, missed cleavages max 1, mass accuracy tolerance 10 ppm, and 0.6 Da for precursors and fragments, respectively. Sequest search, Percolator, and INFERYS rescoring algorithm nodes were used. Carbamidomethylcysteine was used as a fixed modification, and methionine oxidation was used as a variable. Proteins were considered identified with at least one unique peptide, using FDR thresholds of 0.01% (strict) and 1% (relaxed). Each analysis was performed in duplicate.

#### Gene set enrichment analysis

Enrichment analysis was performed with GSEA (version 4.3.1) via the hallmark gene set database to investigate differences between MIAPaCa-2 and PL45 cells and the effect of LDHA inhibition in both cell lines by comparing the effects of oxamate treatment with those of the control via normalized intensities. Parameters used for analysis: nperm 1000 and gene set permutation.

#### Metabolome and lipidome extraction and analysis

The cell pellets were extracted with a mixture of ice-cold MeOH/H_2_O/MTBE as previously reported^52^. Metabolome analyses were performed on a Vanquish Flex UHPLC system (Thermo Fisher Scientific) coupled online to a quadrupole Orbitrap Exploris 120 hybrid mass spectrometer (Thermo Fisher Scientific) equipped with a heated electrospray ionization (HESI-II) probe. Lipidome analysis was performed on an Ultimate RS 3000 UHPLC (Thermo Fisher Scientific) coupled online to a quadrupole time-of-flight timsTOF Pro mass spectrometer (Bruker Daltonics) equipped with an Apollo II electrospray ionization (ESI) probe. Metabolomic and lipidomic analyses were carried out as reported previously.^53^

Each dataset (proteomics, lipidomics, and metabolomics) was independently preprocessed. Proteomics and metabolomics datasets were normalized by the total ion sum, whereas lipidomics data were normalized against class-specific internal standards.

Metabolomic analysis was performed via Compound Discoverer (version 3.3) (Thermo Fisher Scientific) to normalize, align, detect, and identify compounds. Features were extracted from 0 to 10 min and 0 to 11 min of the HILIC run, respectively, in the *m*/*z* = 70–800 mass range. The data were aligned according to an adaptive curve alignment model. The compounds were detected via the following parameter settings: the mass tolerance was set to 5 ppm, the retention time tolerance was set to 0.2 min; the minimum peak intensity was set to 100,000 a.u.; and the signal-to-noise threshold for compound detection was set to 5. The peak rating filter was set to 3. To perform blank subtraction, a maximum sample/max blank >5 was maintained. To predict the elemental compositions of the compounds, the relative intensity tolerance was set to 30% for isotope pattern matching. For the mzCloud database search, both the precursor and fragment mass tolerances were set to 5 ppm. The databases used for matching compounds in ChemSpider for structural searches were BioCyc, the Human Metabolome Database, and KEGG, and the mass tolerance in ChemSpider Search was set to 5 ppm. The mass tolerance for matching compounds in the Metabolika pathway was set to 5 ppm. Compounds were assigned by comparing annotations via the following nodes in order of priority: (1) mzCloud; (2) predicted compositions; (3) mass list search; (4) ChemSpider search; and (5) Metabolika search.

#### Downstream analysis via metabolomics

Metabolite enrichment and network pathway analysis were performed to investigate significantly enriched processes functionally related to metabolite amount via the MetaboAnalyst R package (version 6.0).^54^ The CalculateOraScore command was used to calculate the overrepresentation analysis score in MIAPaCa-2 and PL45 cells at the basal level and treated with oxamate. The PlotPathSummary command was used to visualize the glycolysis/gluconeogenesis pathway.

#### Metabolites in PDAC patients

The PDAC patient cohort used to investigate metabolites positively and negatively correlated with lactate levels, and for comparison with PDAC cellular models, was downloaded from the Zenodo database (https://zenodo.org/), as reported previously.^55^

#### Preprocessing and analysis for lipidomics

MetaboScape 2023b (Bruker) was used for lipidomic data processing and lipid annotation. The number of features detected was set to 500 and 250 counts for the positive and negative modes, respectively. The minimum number of data points was set to 100, and recursive feature extraction was used (75 points). Lipid annotation was first performed with a rule-based annotation, which was based on diagnostic class-specific fragments and their intensity in corresponding MS/MS spectra, and subsequently, the LipidBlast spectral library (http://prime.psc.riken.jp/compms/msdial/main.html) was used. The following parameters were set: mass accuracy window, narrow 2 ppm, wide 10 ppm; mSigma, narrow 30, wide 250; MS/MS score, narrow 800, wide 150; collision cross-section (CCS) %, narrow 1, wide 3.5. The spectra were processed in ESI^+^ mode as adducts [M + H]^+^, [M+Na]^+^, [M + K]^+^, [M + H−H_2_O]^+^, and [M + NH_4_]^+^ ions, whereas they were processed in ESI^−^ [M−H]^−^, [M+Cl]^−^, [M + HCOO]^−^, and [M−H_2_O]^−^. CCS values were matched with those predicted by the CCSbase platform (https://ccsbase.net/) and CCS-Predict tool by MetaboScape; Smart Formula™ (SF) was used for molecular formula assignment. Manual curation of each lipid was then performed following Lipidomics Standard Initiative guidelines (https://lipidomics-standards-initiative.org/guidelines/lipid-species-identification/general-rules). Specifically, in addition to MS/MS diagnostic ions, crucial aspects of lipid annotation, such as (i) lipid adducts in electrospray ionization and (ii) regular retention behavior, e.g., the equivalent carbon number model used for RPLC, were carefully evaluated. The LipidCreator tool (https://lifs-tools.org/lipidcreator.html) extension in Skyline (https://skyline.ms/project/home/begin.view) was used for in silico comparisons of specific product ions.

#### Downstream analysis via lipidomics

The R package LipidomeR (version 0.1.2) was used to analyze the lipidomic content through a regression model fitted on the dataset. Lipids were categorized by lipid class and presented on two-dimensional maps organized by size and level of saturation. Lipidome-wide heatmaps of statistical associations were created to evaluate covariates and lipid content. Statistical significance was calculated via the *F* test. The compute_models_with_limma and compute_F_test_with_limma commands were used to compute multiple regression models and F_test, respectively.

### Integration analysis

To better elucidate the detailed functional mechanism and cross-correlation in MIAPaCa-2 cells upon LDHA inhibition, an integrated analysis of the proteomics, metabolomics, and lipidomics datasets was performed with the mixOmics R package (version 6.28.0)^56^ applying N-integration. Partial least squares discriminant analysis (PLS-DA) and data integration analysis for biomarker discovery using latent variable approaches for omics studies (DIABLO) were used to calculate the number of components, maximize the separation between groups, and account for correlations between the omics datasets. Integrated proteome, metabolome, and lipidome data were visualized with a heatmap, Circos plot, and network plot by extracting the strongest connections, with correlations >0.7 and <−0.7, across datasets between groups. Cross-correlated features (Supplementary Table 6) were further investigated with DAVID for functional annotation of proteins, whereas MetaboAnalystR and LIPEA (https://hyperlipea.org/home) were used for enrichment analysis of metabolite and lipid datasets, reporting results with *p* values < 0.05.

### Pathview

To visualize integrated metabolite and protein data, expression and metabolomic profiles were analyzed via the pathview package v.1.44.0 in R. Pathview enables automatic mapping and integration of experimental data onto KEGG metabolic pathways, providing a graphical representation of molecular changes. Protein expression and metabolite level data were preprocessed and normalized prior to analysis. The identifiers were converted to the appropriate KEGG IDs to allow accurate mapping onto the pathways. Analysis was performed via the pathview() function, with differential expression values supplied as input vectors corresponding to each metabolite or protein, along with the KEGG pathway ID of interest. Pathways investigated include the following: hsa00030 PENTOSE PHOSPHATE PATHWAY, hsa00190 OXIDATIVE PHOSPHORYLATION, and hsa00020 CITRATE CYCLE (TCA). Color coding was applied to represent the up- and downregulation of molecules within the metabolic maps for clear visualization.

## Supplementary information

Supplementary Materials

Original files Western Blot

Supplementary Video 1

Supplementary Video 2

## Data Availability

The spatial transcriptomics data generated in this study have been deposited in NCBI’s Gene Expression Omnibus (GEO) and are accessible through the following GEO series accession number: GSE303338. Omics data on metabolomics, proteomics, and lipidomics generated in this study have been deposited in Zenodo (10.5281/zenodo.15921521). The transcriptomic and clinical data of the TCGA-PAAD, CPTAC-PAAD, and E-MTAB-6134 cohorts were retrieved from the TCGA data portal (https://portal.gdc.cancer.gov/), CPTAC data portal (https://gdc.cancer.gov/), and OmicsDI (https://www.omicsdi.org/), respectively. Single-cell RNA-seq data for pancreatic cancer and clinical information were retrieved from Zenodo (https://zenodo.org/) with accession number CRA001160 and from NCBI (https://www.ncbi.nlm.nih.gov/) with accession number GSE263733. Data on the metabolites of PDAC patients were retrieved from Zenodo (10.5281/zenodo.7150252).

## References

[1] Bengtsson, A., Andersson, R. & Ansari, D. The actual 5-year survivors of pancreatic ductal adenocarcinoma based on real-world data. *Sci. Rep.* **10**, 16425 (2020). 33009477 10.1038/s41598-020-73525-y PMC7532215

[2] Nakaoka, K. et al. Current Status of the diagnosis of early-stage pancreatic ductal adenocarcinoma. *Diagnostics* **13**, 215 (2023). 36673023 10.3390/diagnostics13020215 PMC9857526

[3] Daemen, A. et al. Metabolite profiling stratifies pancreatic ductal adenocarcinomas into subtypes with distinct sensitivities to metabolic inhibitors. *Proc. Natl. Acad. Sci. USA* **112**, E4410–E4417 (2015). 26216984 10.1073/pnas.1501605112 PMC4538616

[4] Karasinska, J. M. et al. Altered gene expression along the glycolysis-cholesterol synthesis axis is associated with outcome in pancreatic cancer. *Clin. Cancer Res.* **26**, 135–146 (2020). 31481506 10.1158/1078-0432.CCR-19-1543

[5] Kaoutari, A. E. et al. Metabolomic profiling of pancreatic adenocarcinoma reveals key features driving clinical outcome and drug resistance. *EBioMedicine* **66**, 103332 (2021). 33862584 10.1016/j.ebiom.2021.103332 PMC8054161

[6] Song, W. et al. Glycolysis-related gene expression profiling screen for prognostic risk signature of pancreatic ductal adenocarcinoma. *Front. Genet.* **12**, 639246 (2021). 34249078 10.3389/fgene.2021.639246 PMC8261051

[7] Espiau-Romera, P., Courtois, S., Parejo-Alonso, B. & Sancho, P. Molecular and metabolic subtypes correspondence for pancreatic ductal adenocarcinoma classification. *J. Clin. Med.* **9**, 4128 (2020). 33371431 10.3390/jcm9124128 PMC7767410

[8] Barthel, S., Falcomata, C., Rad, R., Theis, F. J. & Saur, D. Single-cell profiling to explore pancreatic cancer heterogeneity, plasticity and response to therapy. *Nat. Cancer* **4**, 454–467 (2023). 36959420 10.1038/s43018-023-00526-x PMC7615362

[9] Lu, Q. Y., Zhang, L., Yee, J. K., Go, V. W. & Lee, W. N. Metabolic consequences of LDHA inhibition by epigallocatechin gallate and oxamate in MIA PaCa-2 pancreatic cancer cells. *Metabolomics* **11**, 71–80 (2015). 26246802 10.1007/s11306-014-0672-8 PMC4523095

[10] Balachandran, V. P., Beatty, G. L. & Dougan, S. K. Broadening the impact of immunotherapy to pancreatic cancer: challenges and opportunities. *Gastroenterology* **156**, 2056–2072 (2019). 30660727 10.1053/j.gastro.2018.12.038 PMC6486864

[11] Heymans, C., Degosserie, J., Spourquet, C. & Pierreux, C. E. Pancreatic acinar differentiation is guided by differential laminin deposition. *Sci. Rep.* **9**, 2711 (2019). 30804366 10.1038/s41598-019-39077-6 PMC6389953

[12] Abou Khouzam, R. et al. Hypoxia, a targetable culprit to counter pancreatic cancer resistance to therapy. *Cancers* **15**, 1235 (2023). 36831579 10.3390/cancers15041235 PMC9953896

[13] Robinson, C. M. et al. An emerging role for the unfolded protein response in pancreatic cancer. *Cancers* **13**, 261 (2021). 33445669 10.3390/cancers13020261 PMC7828145

[14] Kern, J. et al. Identification of the unfolded protein response pathway as target for radiosensitization in pancreatic cancer. *Radiother. Oncol.* **191**, 110059 (2024). 38135186 10.1016/j.radonc.2023.110059

[15] Xiong, G. et al. Collagen prolyl 4-hydroxylase 1 is essential for HIF-1alpha stabilization and TNBC chemoresistance. *Nat. Commun.* **9**, 4456 (2018). 30367042 10.1038/s41467-018-06893-9 PMC6203834

[16] Snell, C. E. et al. Proline-hydroxylated hypoxia-inducible factor 1alpha (HIF-1alpha) upregulation in human tumours. *PLoS ONE* **9**, e88955 (2014). 24563687 10.1371/journal.pone.0088955 PMC3923075

[17] Cheng, C. S. et al. Functional inhibition of lactate dehydrogenase suppresses pancreatic adenocarcinoma progression. *Clin. Transl. Med.* **11**, e467 (2021). 34185423 10.1002/ctm2.467 PMC8238920

[18] Michalkova, L. et al. Early detection of pancreatic cancer in type 2 diabetes mellitus patients based on (1)H NMR metabolomics. *J. Proteome Res.* **20**, 1744–1753 (2021). 33617266 10.1021/acs.jproteome.0c00990

[19] Yang, J. S., Wang, C. C., Qiu, J. D., Ren, B. & You, L. Arginine metabolism: a potential target in pancreatic cancer therapy. *Chin. Med. J.* **134**, 28–37 (2020). 33395072 10.1097/CM9.0000000000001216 PMC7862822

[20] Moir, J. A. G. et al. Therapeutic strategies toward lactate dehydrogenase within the tumor microenvironment of pancreatic cancer. *Pancreas* **49**, 1364–1371 (2020). 33122526 10.1097/MPA.0000000000001689

[21] Di Magno, L. et al. Discovery of novel human lactate dehydrogenase inhibitors: Structure-based virtual screening studies and biological assessment. *Eur. J. Med. Chem.* **240**, 114605 (2022). 35868126 10.1016/j.ejmech.2022.114605

[22] Ye, W. et al. Oxamate improves glycemic control and insulin sensitivity via inhibition of tissue lactate production in db/db mice. *PLoS ONE* **11**, e0150303 (2016). 26938239 10.1371/journal.pone.0150303 PMC4777529

[23] Peng, J. et al. Single-cell RNA-seq highlights intra-tumoral heterogeneity and malignant progression in pancreatic ductal adenocarcinoma. *Cell Res.* **29**, 725–738 (2019). 31273297 10.1038/s41422-019-0195-y PMC6796938

[24] Park, J. K. et al. Single-cell transcriptome analysis reveals subtype-specific clonal evolution and microenvironmental changes in liver metastasis of pancreatic adenocarcinoma and their clinical implications. *Mol. Cancer* **23**, 87 (2024). 38702773 10.1186/s12943-024-02003-0 PMC11067162

[25] Satija, R., Farrell, J. A., Gennert, D., Schier, A. F. & Regev, A. Spatial reconstruction of single-cell gene expression data. *Nat. Biotechnol.* **33**, 495–502 (2015). 25867923 10.1038/nbt.3192 PMC4430369

[26] Butler, A., Hoffman, P., Smibert, P., Papalexi, E. & Satija, R. Integrating single-cell transcriptomic data across different conditions, technologies, and species. *Nat. Biotechnol.* **36**, 411–420 (2018). 29608179 10.1038/nbt.4096 PMC6700744

[27] Stuart, T. et al. Comprehensive integration of single-cell data. *Cell* **177**, 1888–1902.e1821 (2019). 31178118 10.1016/j.cell.2019.05.031 PMC6687398

[28] Hao, Y. et al. Integrated analysis of multimodal single-cell data. *Cell* **184**, 3573–3587.e3529 (2021). 34062119 10.1016/j.cell.2021.04.048 PMC8238499

[29] Hao, Y. et al. Dictionary learning for integrative, multimodal and scalable single-cell analysis. *Nat. Biotechnol.* **42**, 293–304 (2024). 37231261 10.1038/s41587-023-01767-y PMC10928517

[30] Korsunsky, I. et al. Fast, sensitive and accurate integration of single-cell data with Harmony. *Nat. Methods* **16**, 1289–1296 (2019). 31740819 10.1038/s41592-019-0619-0 PMC6884693

[31] Ren, X. et al. Single-cell RNA-seq reveals invasive trajectory and determines cancer stem cell-related prognostic genes in pancreatic cancer. *Bioengineered* **12**, 5056–5068 (2021). 34474642 10.1080/21655979.2021.1962484 PMC8806718

[32] Wang, Y. et al. Single-cell analysis of pancreatic ductal adenocarcinoma identifies a novel fibroblast subtype associated with poor prognosis but better immunotherapy response. *Cell Discov.* **7**, 36 (2021). 34035226 10.1038/s41421-021-00271-4 PMC8149399

[33] Kuleshov, M. V. et al. Enrichr: a comprehensive gene set enrichment analysis web server 2016 update. *Nucleic Acids Res.* **44**, W90–W97 (2016). 27141961 10.1093/nar/gkw377 PMC4987924

[34] Zollinger, D. R., Lingle, S. E., Sorg, K., Beechem, J. M. & Merritt, C. R. GeoMx RNA Assay: high multiplex, digital, spatial analysis of RNA in FFPE tissue. *Methods Mol. Biol.* **2148**, 331–345 (2020). 32394392 10.1007/978-1-0716-0623-0_21

[35] Danaher, P. et al. Advances in mixed cell deconvolution enable quantification of cell types in spatial transcriptomic data. *Nat. Commun.* **13**, 385 (2022). 35046414 10.1038/s41467-022-28020-5 PMC8770643

[36] Kumar, L. & E Futschik, M. Mfuzz: a software package for soft clustering of microarray data. *Bioinformation* **2**, 5–7 (2007). 18084642 10.6026/97320630002005 PMC2139991

[37] Puleo, F. et al. Stratification of pancreatic ductal adenocarcinomas based on tumor and microenvironment features. *Gastroenterology* **155**, 1999–2013.e1993 (2018). 30165049 10.1053/j.gastro.2018.08.033

[38] Peran, I., Madhavan, S., Byers, S. W. & McCoy, M. D. Curation of the pancreatic ductal adenocarcinoma subset of The Cancer Genome Atlas is essential for accurate conclusions about survival-related molecular mechanisms. *Clin. Cancer Res.***24**, 3813–3819 (2018).29739787 10.1158/1078-0432.CCR-18-0290

[39] Love, M. I., Huber, W. & Anders, S. Moderated estimation of fold change and dispersion for RNA-seq data with DESeq2. *Genome Biol.* **15**, 550 (2014). 25516281 10.1186/s13059-014-0550-8 PMC4302049

[40] Gautier, L., Cope, L., Bolstad, B. M. & Irizarry, R. A. affy—analysis of Affymetrix GeneChip data at the probe level. *Bioinformatics* **20**, 307–315 (2004). 14960456 10.1093/bioinformatics/btg405

[41] Mootha, V. K. et al. PGC-1alpha-responsive genes involved in oxidative phosphorylation are coordinately downregulated in human diabetes. *Nat. Genet.* **34**, 267–273 (2003). 12808457 10.1038/ng1180

[42] Tang, Z. et al. GEPIA: a web server for cancer and normal gene expression profiling and interactive analyses. *Nucleic Acids Res.* **45**, W98–W102 (2017). 28407145 10.1093/nar/gkx247 PMC5570223

[43] Eustace, A. et al. A 26-gene hypoxia signature predicts benefit from hypoxia-modifying therapy in laryngeal cancer but not bladder cancer. *Clin. Cancer Res.* **19**, 4879–4888 (2013). 23820108 10.1158/1078-0432.CCR-13-0542 PMC3797516

[44] Moffitt, R. A. et al. Virtual microdissection identifies distinct tumor- and stroma-specific subtypes of pancreatic ductal adenocarcinoma. *Nat. Genet.* **47**, 1168–1178 (2015). 26343385 10.1038/ng.3398 PMC4912058

[45] Jaffee, E. M. et al. Development and characterization of a cytokine-secreting pancreatic adenocarcinoma vaccine from primary tumors for use in clinical trials. *Cancer J. Sci. Am.* **4**, 194–203 (1998). 9612602

[46] Kyriazis, A. P. et al. Establishment and characterization of human pancreatic adenocarcinoma cell line SW-1990 in tissue culture and the nude mouse. *Cancer Res.* **43**, 4393–4401 (1983). 6871872

[47] Lieber, M., Mazzetta, J., Nelson-Rees, W., Kaplan, M. & Todaro, G. Establishment of a continuous tumor-cell line (panc-1) from a human carcinoma of the exocrine pancreas. *Int. J. Cancer* **15**, 741–747 (1975). 1140870 10.1002/ijc.2910150505

[48] Kim, Y. W., Kern, H. F., Mullins, T. D., Koriwchak, M. J. & Metzgar, R. S. Characterization of clones of a human pancreatic adenocarcinoma cell line representing different stages of differentiation. *Pancreas* **4**, 353–362 (1989). 2734279 10.1097/00006676-198906000-00013

[49] Yunis, A. A., Arimura, G. K. & Russin, D. J. Human pancreatic carcinoma (MIA PaCa-2) in continuous culture: sensitivity to asparaginase. *Int. J. Cancer* **19**, 128–135 (1977). 832918 10.1002/ijc.2910190118

[50] Chianese, U. et al. FASN multi-omic characterization reveals metabolic heterogeneity in pancreatic and prostate adenocarcinoma. *J. Transl. Med.* **21**, 32 (2023). 36650542 10.1186/s12967-023-03874-5 PMC9847120

[51] Clark, N. A. et al. GRcalculator: an online tool for calculating and mining dose-response data. *BMC Cancer* **17**, 698 (2017). 29065900 10.1186/s12885-017-3689-3 PMC5655815

[52] Scisciola, L. et al. Multi-omics analysis reveals attenuation of cellular stress by empagliflozin in high glucose-treated human cardiomyocytes. *J. Transl. Med.* **21**, 662 (2023). 37742032 10.1186/s12967-023-04537-1 PMC10518098

[53] Caponigro, V. et al. Integrated plasma metabolomics and lipidomics profiling highlights distinctive signature of hepatocellular carcinoma in HCV patients. *J. Transl. Med.* **21**, 918 (2023). 38110968 10.1186/s12967-023-04801-4 PMC10729519

[54] Chong, J. & Xia, J. MetaboAnalystR: an R package for flexible and reproducible analysis of metabolomics data. *Bioinformatics* **34**, 4313–4314 (2018). 29955821 10.1093/bioinformatics/bty528 PMC6289126

[55] Benedetti, E. et al. A multimodal atlas of tumour metabolism reveals the architecture of gene-metabolite covariation. *Nat. Metab.* **5**, 1029–1044 (2023). 37337120 10.1038/s42255-023-00817-8 PMC10290959

[56] Rohart, F., Gautier, B., Singh, A. & Le Cao, K. A. mixOmics: an R package for ‘omics feature selection and multiple data integration. *PLoS Comput. Biol.* **13**, e1005752 (2017). 29099853 10.1371/journal.pcbi.1005752 PMC5687754

