# Supplementary material for: Glycolytic heterogeneity drives metabolic-targeted therapy in pancreatic ductal adenocarcinoma
Source: Signal Transduct Target Ther. 2026 Jan 20;11:25. doi: 10.1038/s41392-025-02546-8 (PMC12816621; doi:10.1038/s41392-025-02546-8)
Supplement: Supplementary file 1 — Supplementary Materials [file 41392_2025_2546_MOESM1_ESM.docx]

Supplementary Materials for

**Glycolytic heterogeneity drives metabolic-targeted therapy in pancreatic ductal adenocarcinoma**

Ugo Chianese^1^, Chiara Papulino^1^, Gerardo Saggese^1^, Ahmad Ali^1^, Marianna Ciotola^2^, Enza Lonardo^2^, Mirko Cortese^3^, Gregorio Favale^1^, Annabella Di Mauro^1,4^, Danila La Gioia^5^, Valentina Golino^5^, Eduardo Sommella^5^, Pietro Campiglia^5^, Renato Franco^6^, Fortunato Ciardiello^1^, Ferdinando De Vita^1^, Vincenzo Carafa^1,7^, Lucia Altucci^1,7,8*‡^, Rosaria Benedetti^1,8*‡^

Correspondence to: Lucia Altucci ([lucia.altucci@unicampania.it](mailto:lucia.altucci@unicampania.it)) and Rosaria Benedetti ([rosaria.benedetti@unicampania.it](mailto:rosaria.benedetti@unicampania.it)). Address: Department of Precision Medicine, University of Campania “Luigi Vanvitelli”, via L. De Crecchio 7, 80138 Naples, Italy

**This PDF file includes:**

Figures. S1 to S14

Captions for Figures. S1 to S14

Tables S1 to S6

Captions for Supplementary Video S1 to S2


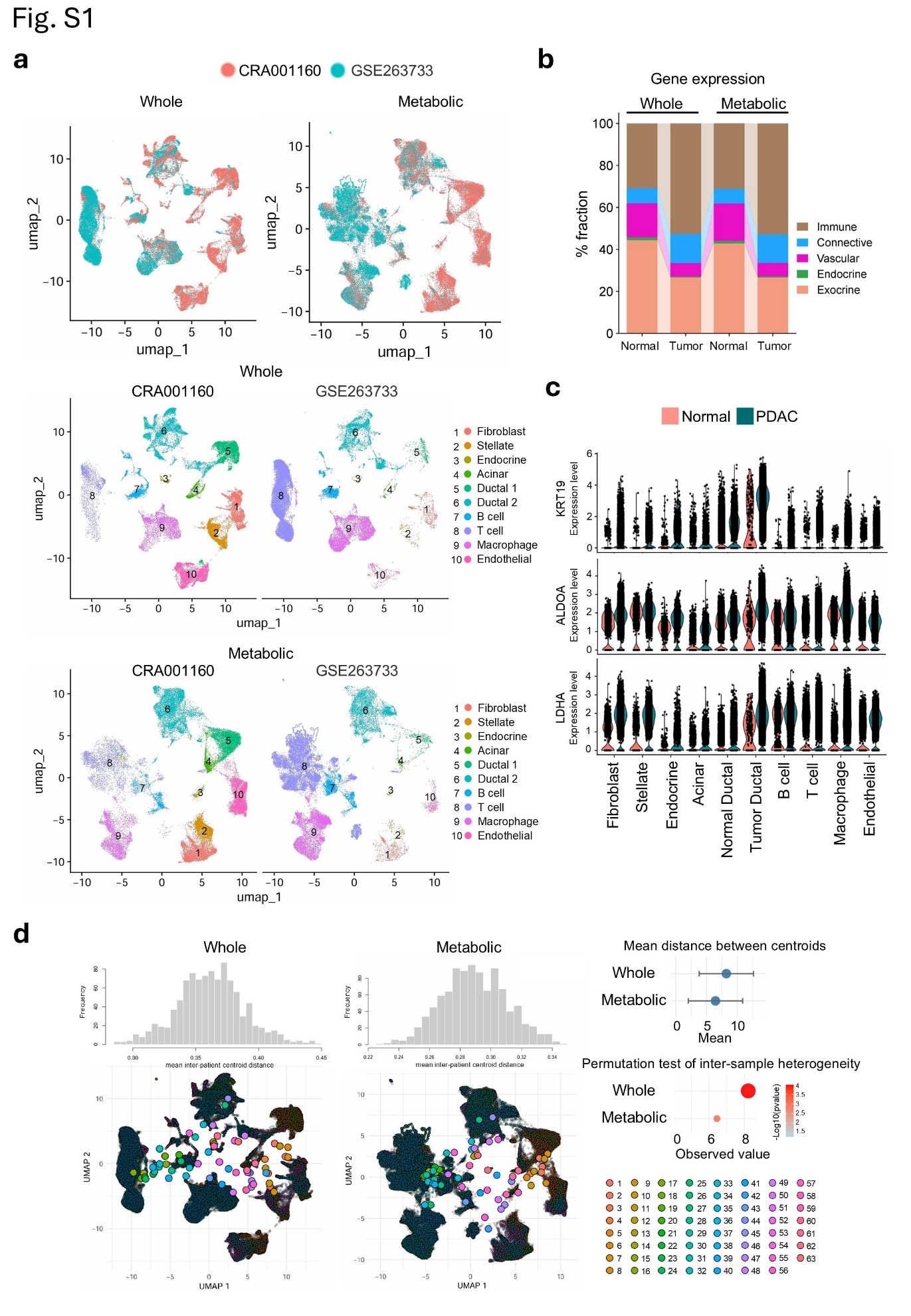


**Supplementary Figure 1: a)** UMAP plots showing cells of PDAC and normal samples for whole gene expression and metabolic genes in CRA001160 and GSE263733 (above), for whole gene expression (middle), and for metabolic genes (below). **b)** Percentage distribution across cellular components in PDAC and normal samples using whole gene expression and metabolic genes. **c)** Violin plots of tumor and glycolytic markers across cell types in PDAC and normal samples. **d)** UMAP plots showing the centroid (mean UMAP coordinates) of PDAC and normal samples for whole gene expression and metabolic genes in CRA001160 and GSE263733. Each point represents the average position of cells from one patient (below). Histogram showing the distribution of mean inter-patient centroid distances obtained from 1000 random permutations of patient labels (above).


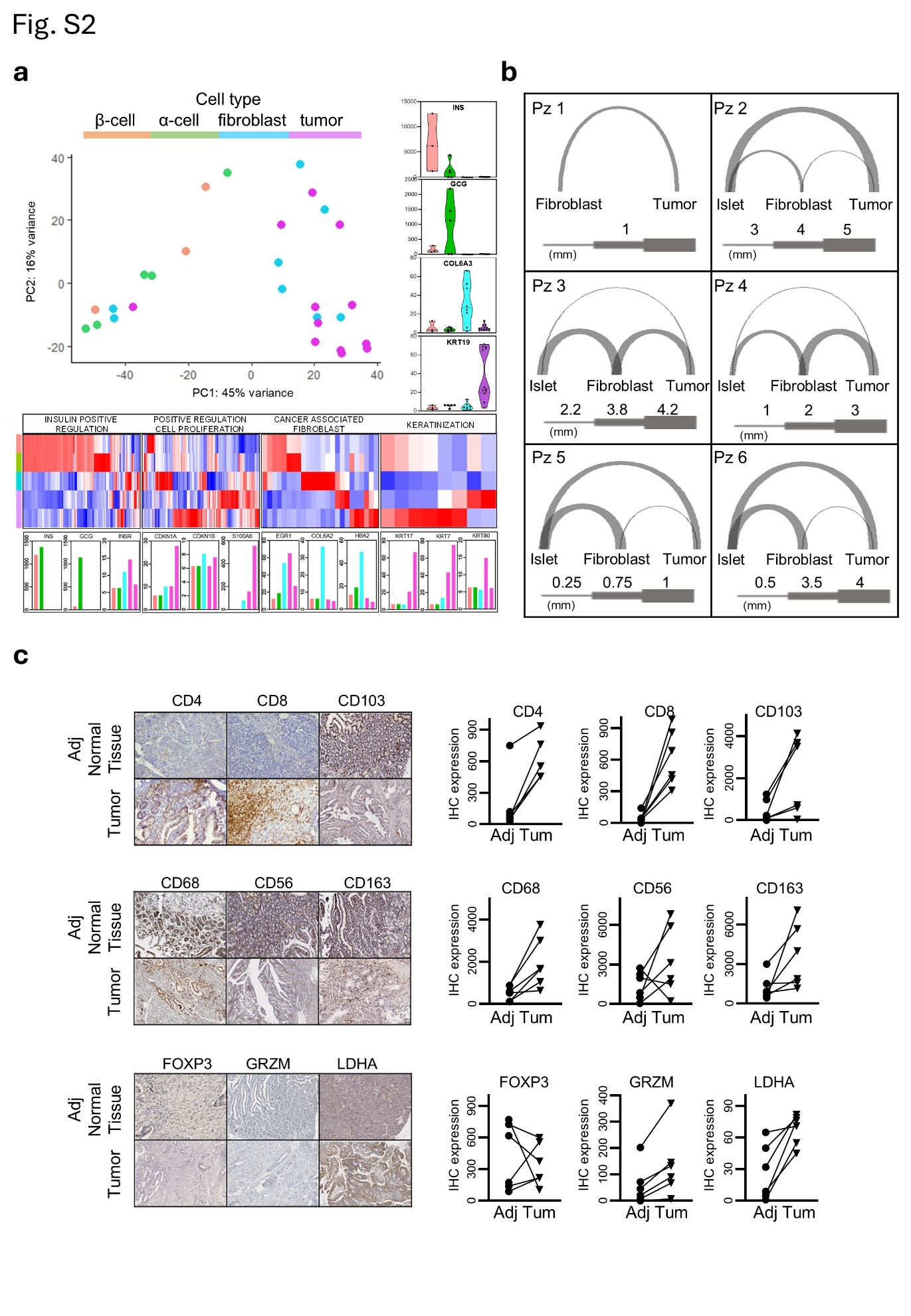
 **Supplementary Figure 2: a)** Principal component analysis (PCA) and violin plot showing cell types defined with spatial transcriptomics and representative marker genes for each cell type, respectively (above). Graph shows biological processes corresponding to cell types (below). **b)** Figure representing geometric distances calculated with spatial transcriptomics between islets, fibroblasts, and tumor cells in each PDAC patient sample. Distances are reported in scale as mm. **c)** Representative image of immunohistochemical (IHC) staining of ductal adenocarcinoma and adjacent normal tissue with CD4, CD8, CD163, CD68, CD103, FOXP3, CD56, CD163, and LDHA markers of patient n°6. Graphs show expression of each marker between ductal adenocarcinoma and adjacent normal tissue in each patient.


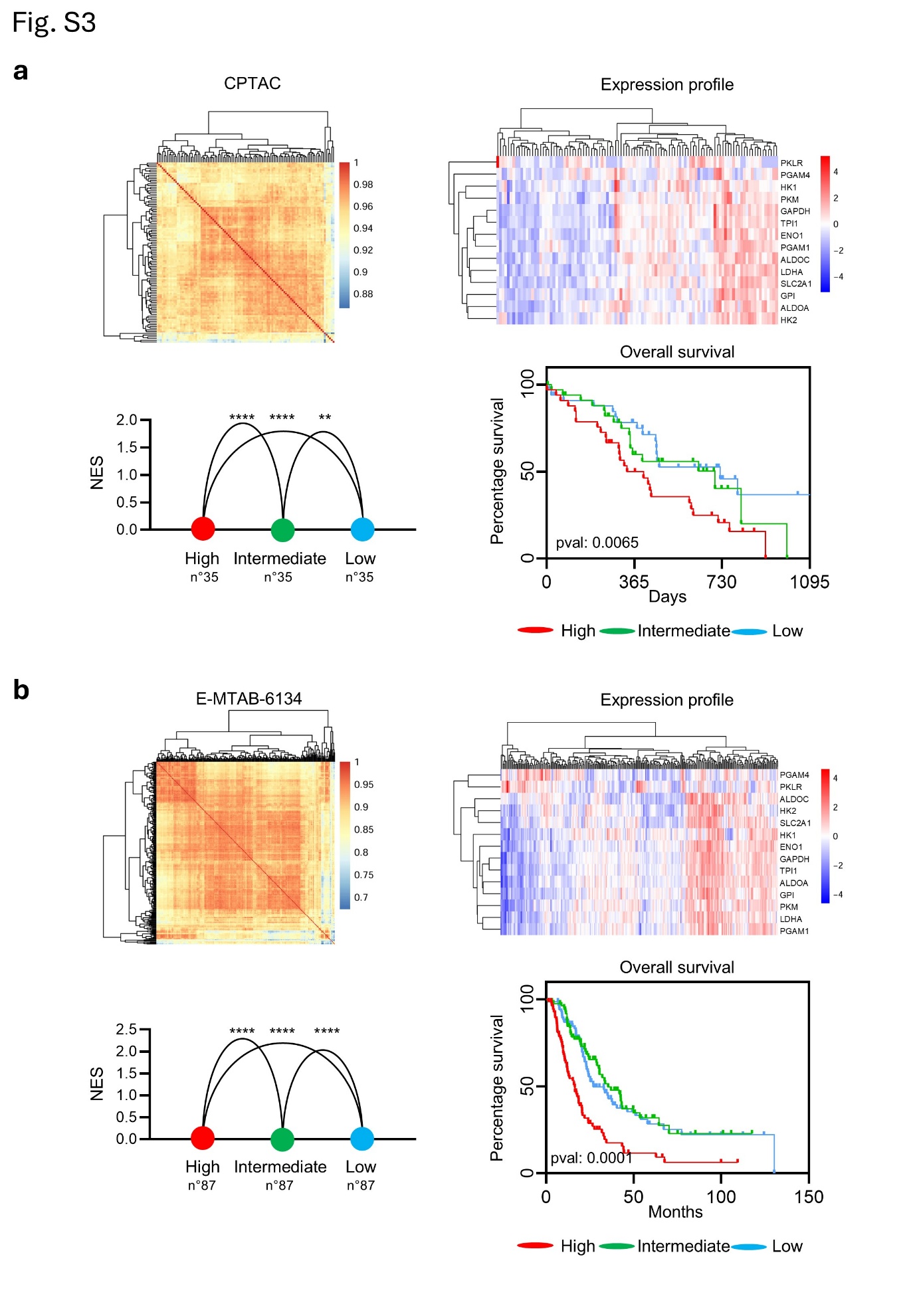


**Supplementary Figure 3: a)** Pairwise correlation of gene expression and unsupervised clustering heatmap of glycolytic signature expression in CPTAC cohort of PDAC patients. CPTAC-PDAC cohort has been divided in tertile and grouped in High, Intermediate, and Low based on glycolytic signature expression. Bottom left, glycolytic enrichment comparison for High vs Intermediate and Low groups; and for Intermediate vs Lowest group. The y-axis reports normalized enrichment score while statistical significance is reported as p-value: <0.0001 ****, <0.001 **. Bottom right, Kaplan–Meier plot showing 3-year overall survival across glycolytic groups in TCGA cohort of PDAC patients. **b)** Pairwise correlation of gene expression and unsupervised clustering heatmap of glycolytic signature expression in E-MTAB-6134 cohort of PDAC patients. E-MTAB-6134-PDAC cohort divided in tertile and grouped in High, Intermediate, and Low based on glycolytic signature expression. Bottom left, glycolytic enrichment comparison for High vs Intermediate and Low groups; and for Intermediate vs Lowest group. The y-axis reports normalized enrichment score while statistical significance is reported as p-value: <0.0001 ****, <0.001 **. Bottom right, Kaplan–Meier plot showing 3-year overall survival across glycolytic groups in E-MTAB-6134 cohort of PDAC patients.


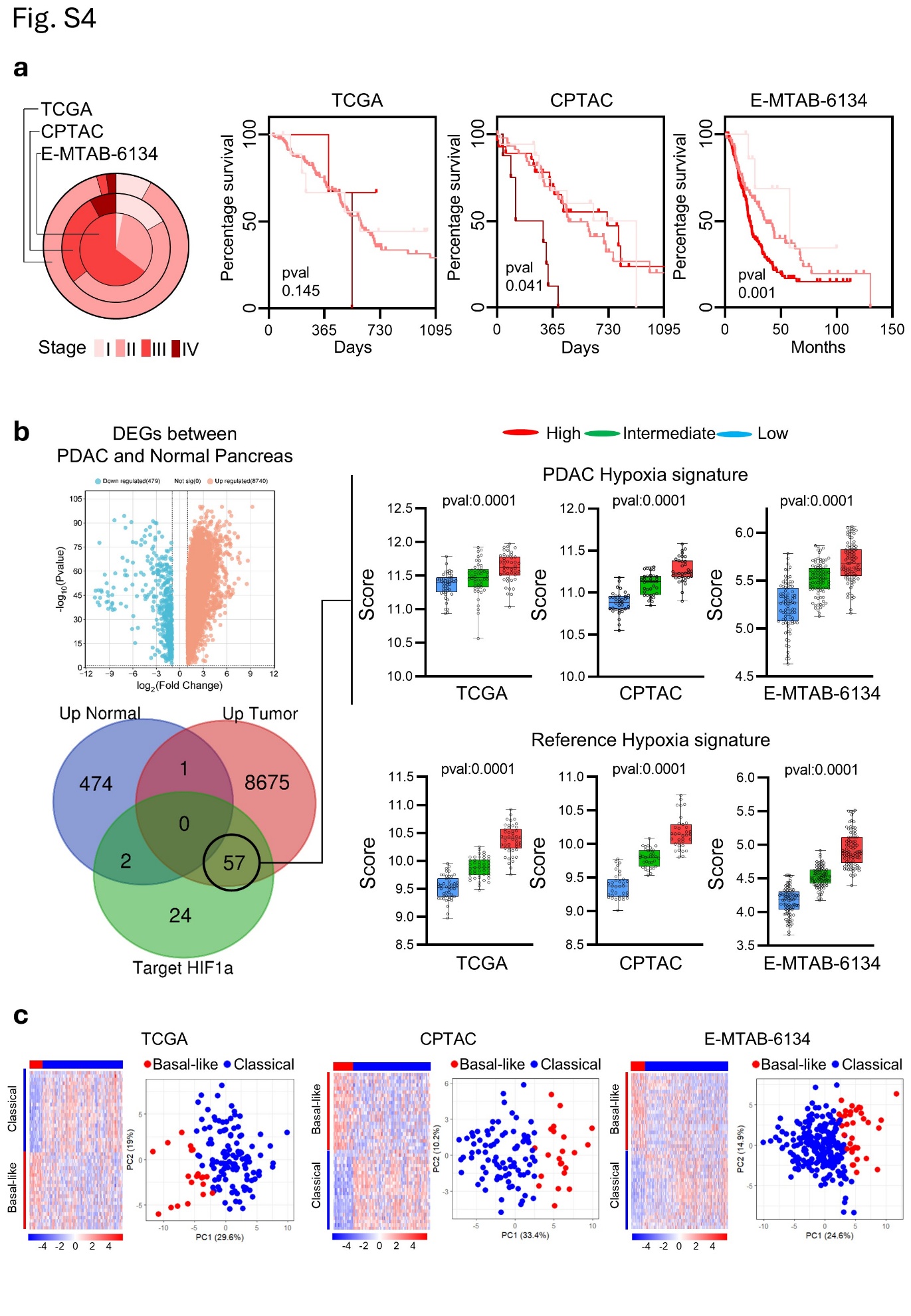


**Supplementary Figure 4: a)** Multiple pie chart with patient percentage stratified by tumor stage (I, II, III, IV) in each of the three analysed cohorts. Kaplan–Meier plot for survival across tumor stages. **b)** Venn diagram of differentially expressed genes in tumor and normal pancreas (left, below), alongside volcano plot (left, above), intersecting with genes transcriptionally regulated by HIF-1α; hypoxia score in glycolytic groups calculated as average of overlapping genes from hypoxia signature and a published hypoxia signature (right); statistical analysis by ANOVA. Each dot represents a patient sample. Data are represented as mean ± SD. **c)** Heatmaps and PCA plots showing the expression patterns of Basal-like and Classical subtype gene signatures across PDAC samples from the TCGA, CPTAC, and E-MTAB-6134 cohorts. Samples are color-coded according to subtype (Basal-like, red; Classical, blue).


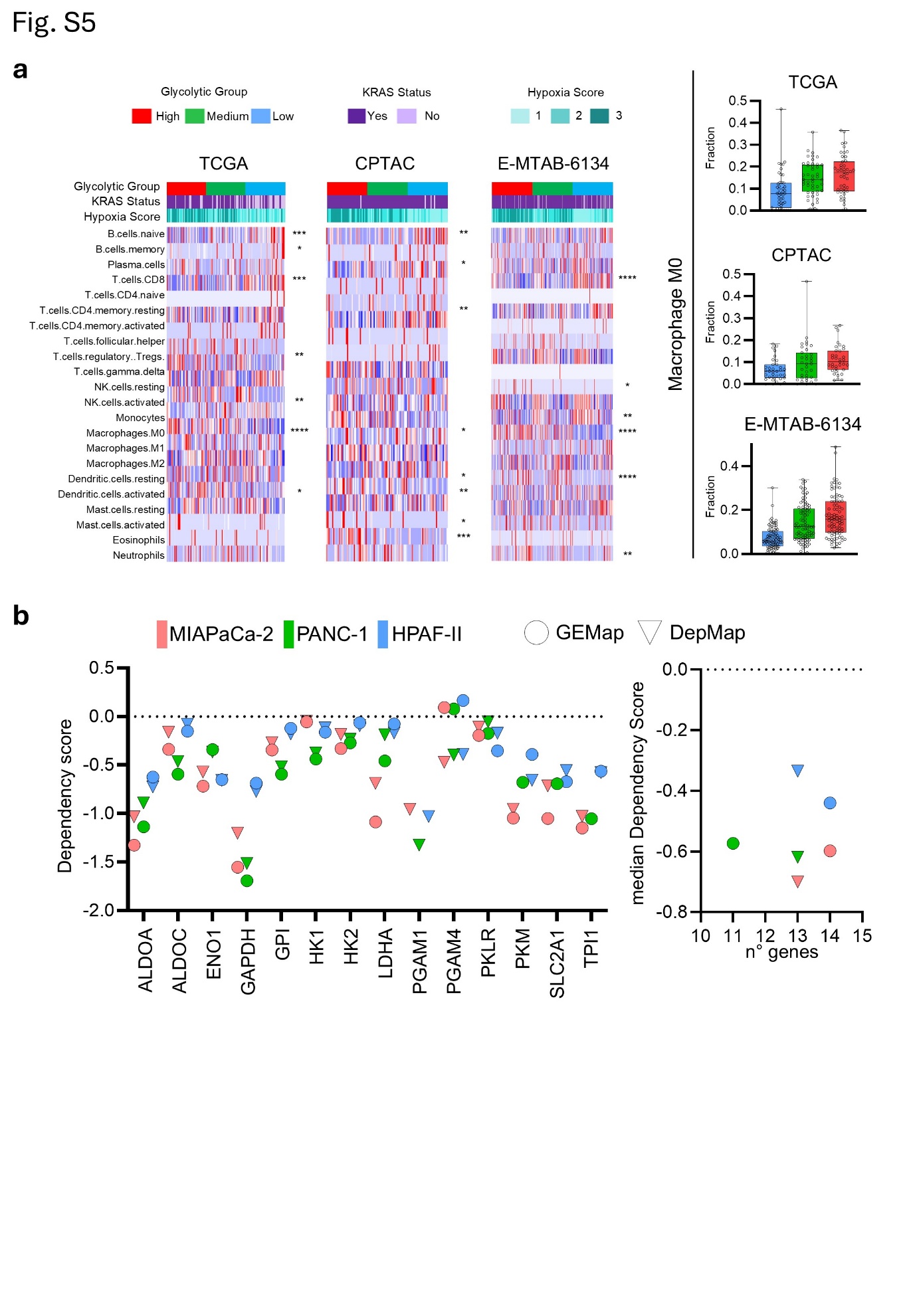
 **Supplementary Figure 5: A)** Heatmap of deconvoluted immune cell fractions from TCGA and the validation cohorts, across patients stratified by glycolytic groups; boxplots showing the relative abundance of M0 macrophages across these groups. Statistical significance is indicated as p-values: * < 0.05, ** < 0.01, **** < 0.0001. Each dot represents a patient sample. Data are represented as mean ± SD. **B)** Plot of gene-level dependency scores and the overall dependency per each cell line for the glycolytic signature genes, retrieved from GEMap (circle) and DepMap (triangle) databases.


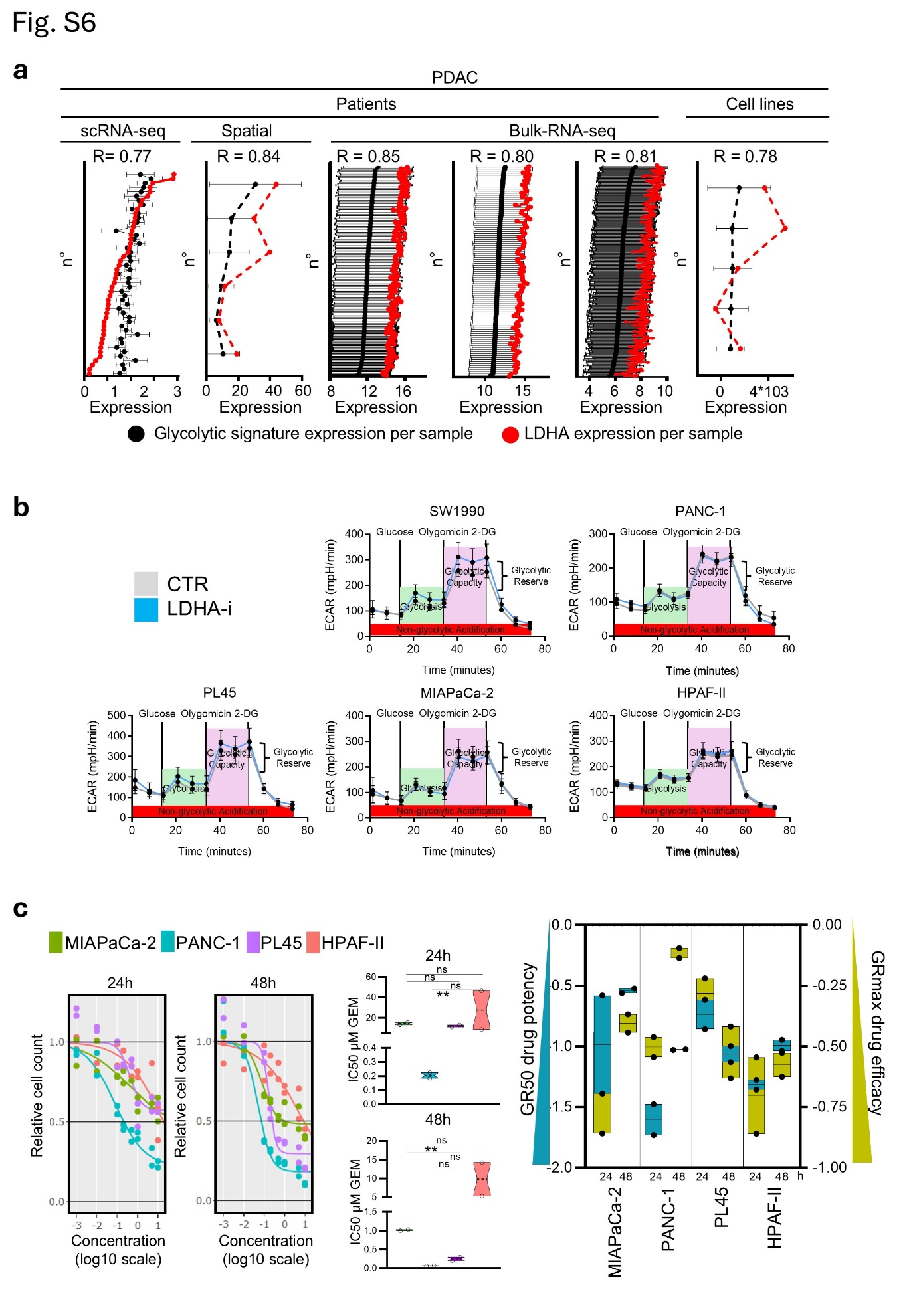


**Supplementary Figure 6: a)** Glycolytic signature and LDHA expression in PDAC patient samples and cell line datasets. CRA001160 and GSE263733 single cell datasets; spatial transcriptomics data generated in this study; TCGA, CPTAC, E-MTAB-6134 bulk rna seq; MIAPaCa-2, PL45, PANC-1, HPAF-II, SW1990 from Human Protein Atlas. **b)** ECAR in PL45, SW1990, PANC-1, MIAPaCa-2, and HPAF-II cells after LDHA-i treatment. **c)** Viability assay in MIAPaCa-2 (green), PANC-1 (blue), PL45 (purple), and HPAF-II (red) cells upon gemcitabine treatment at 24 h and 48 h. Boxplots show IC_50_ values from each time point incell lines and statistical significance calculated with paired t-test analysis (left) (n= 2 per group), and values of GR_50_ and GR_max_ referring to drug potency and efficacy, respectively (right).


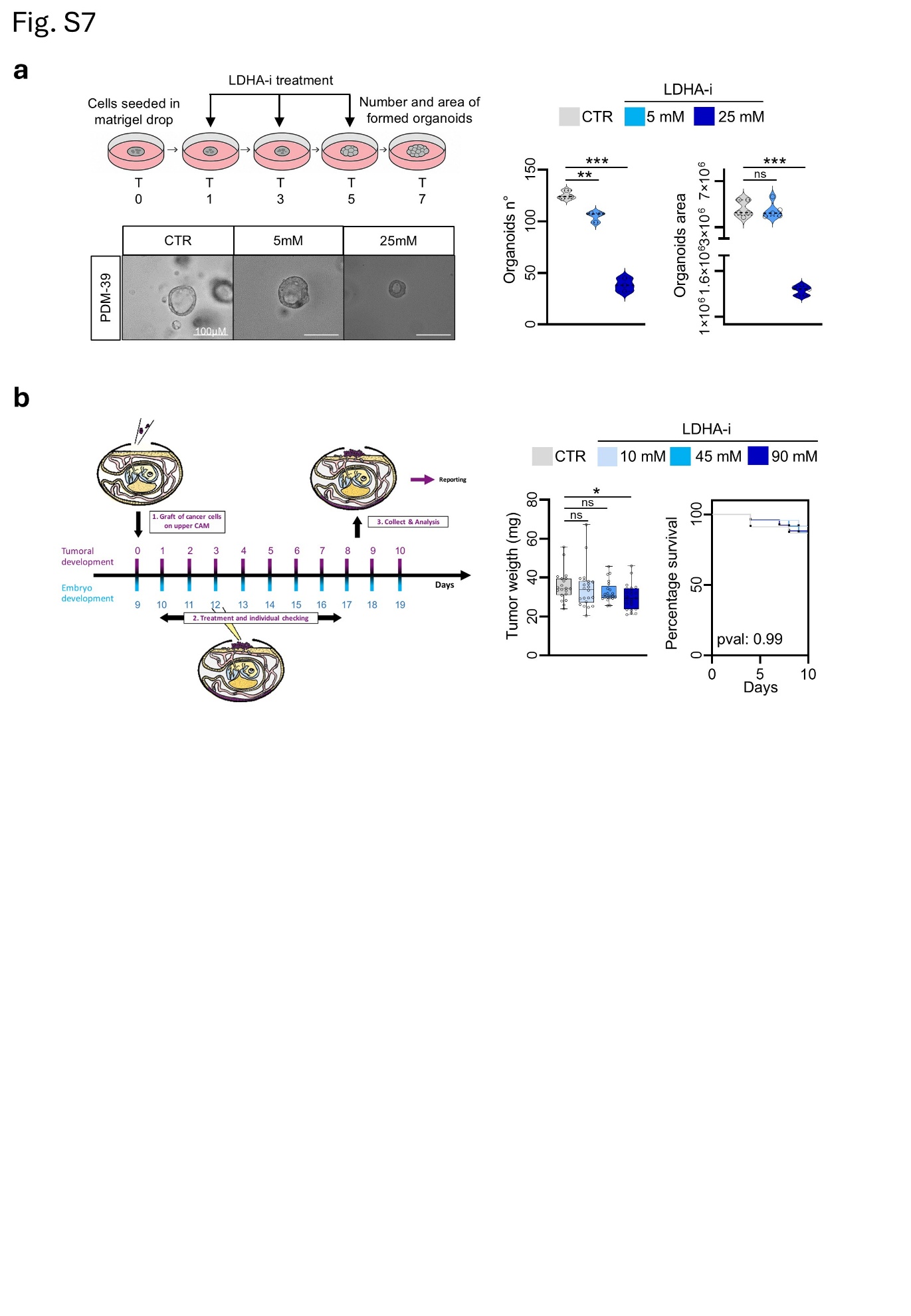


**Supplementary Figure 7: a**) Schematic representation of the experimental timeline. Representative bright-field images of organoids at T7 from cultures treated with vehicle, 5 mM, or 25 mM oxamate. Scale bar, 100 µm. Graphs show organoid count and total organoid area. Data are presented as mean ± SEM of at least three independent replicates. *p < 0.05, **p < 0.01, ***p < 0.001 compared to vehicle control. Each dot represents a biological replicate. Data are represented as mean ± SD. **b)** Schematic representation of the experimental timeline. Graph shows tumor weight of PDAC xenograft. Each dot represents a biological replicate. Data are represented as mean ± SD. Kaplan-Meier curve of survival rate for all group during the study.


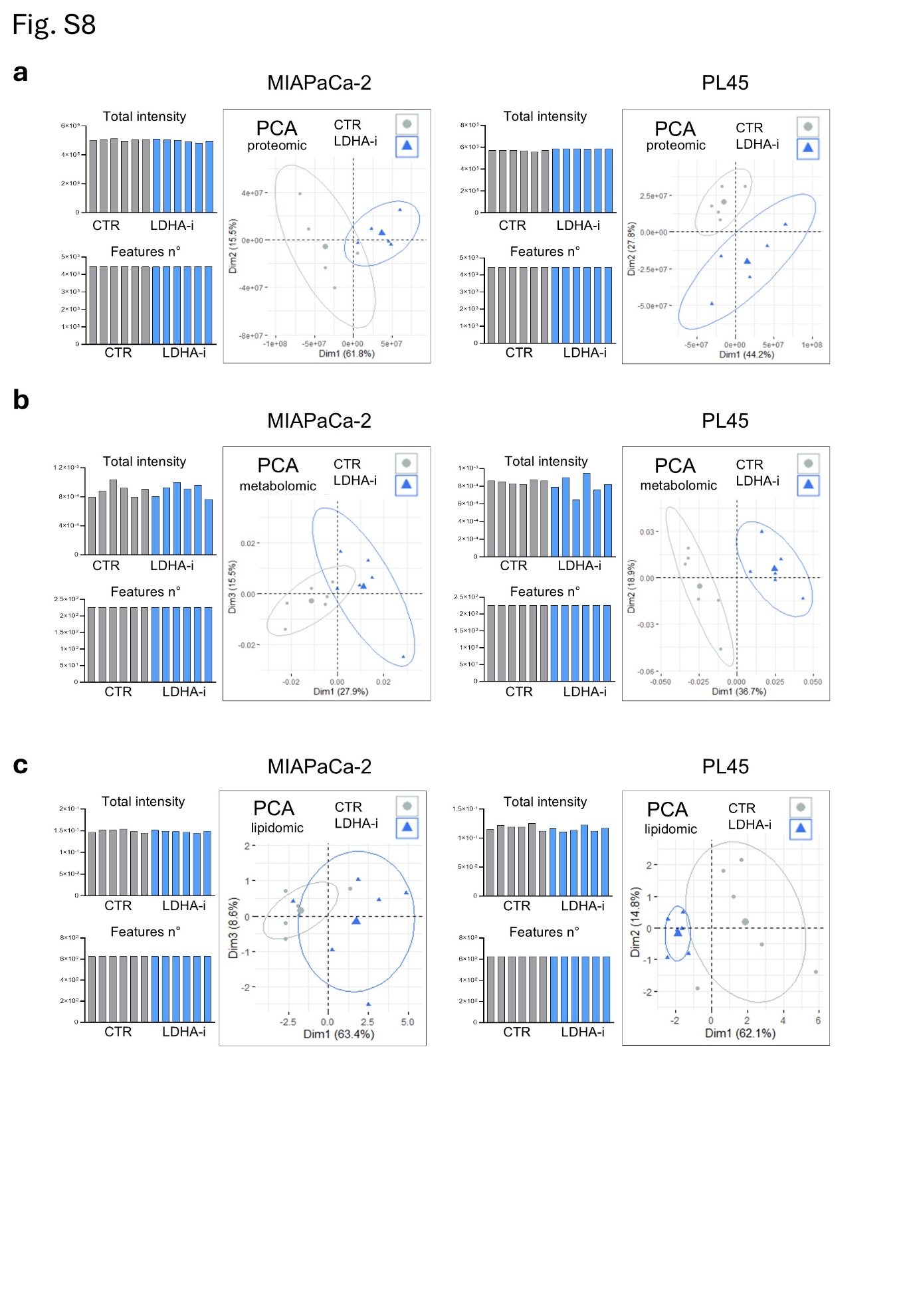


**Supplementary Figure 8:** PCA analyses of proteomic **(a)**, metabolomic **(b),** and lipidomic **(c)** data from MIAPaCa-2 and PL45 cells at basal level and after LDHA-i treatment.


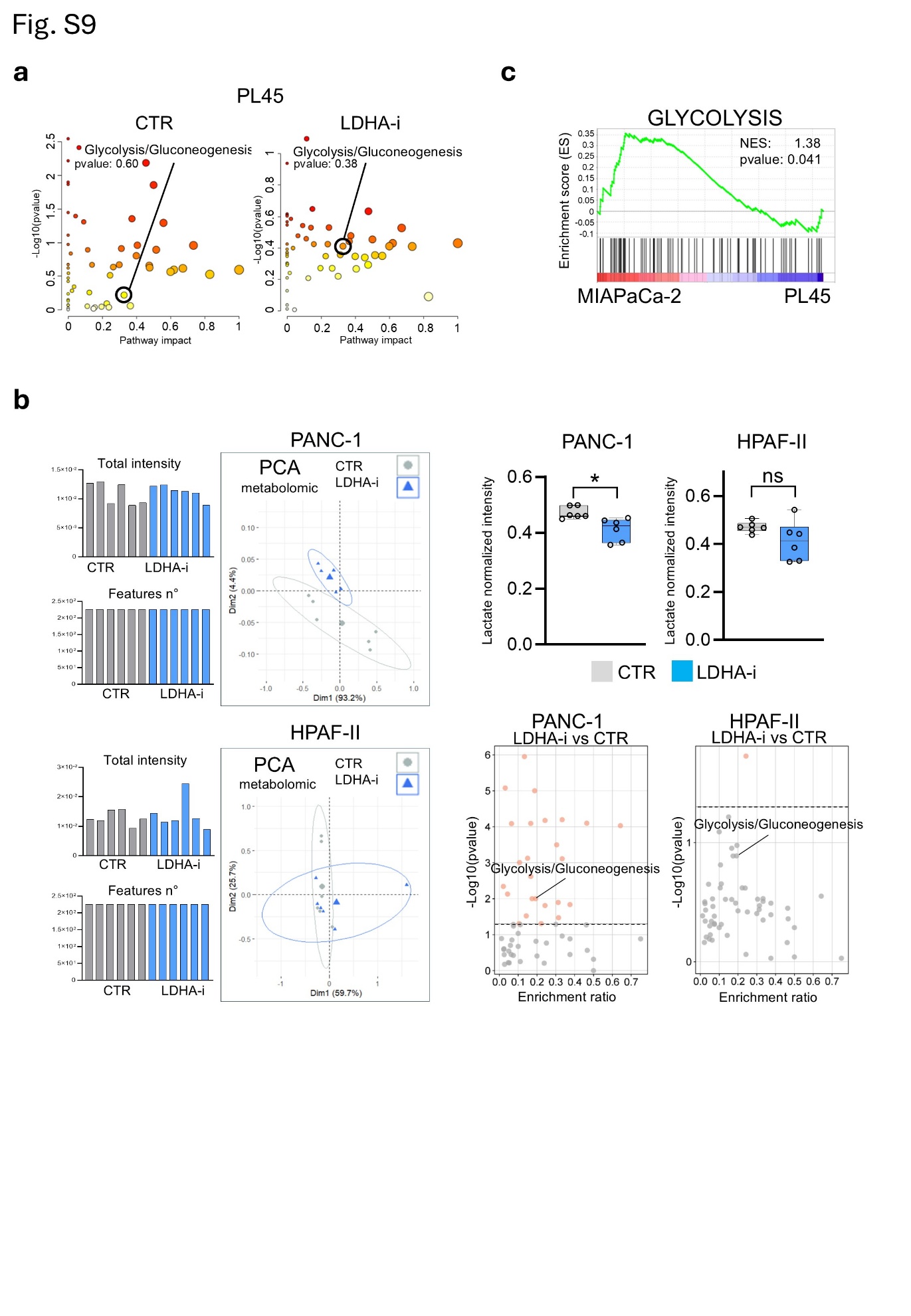


**Supplementary Figure 9: a)** Pathway enrichment analysis of metabolomics data in PL45 cells under basal conditions and following LDHA inhibition. The x-axis represents pathway impact based on metabolite expression, while the y-axis shows statistical significance expressed as –log₁₀(p-value). **b)** Metabolomic PCA analysis of PANC-1 and HPAF-II cells at basal level and treated with LDHA-i. Amount of the lactate in PANC-1 and HPAF-II cells at basal level (grey) and after LDHA inhibition (blue). Each dot represents a biological replicate (n= 6 per group). Data are shown as normalized intensity and are represented as mean ± SD. Statistical significance was calculated with paired t-test. Pathway enrichment analysis performed on comparison of metabolomics data in PANC-1 and HPAF-II after LDHA inhibition. The x-axis indicates enrichment ratio of metabolic pathway. The y-axis indicates p-value transformed as –Log10. **c)** Enrichment plot comparing glycolytic pathway activation between MIAPaCa-2 and PL45 cells at basal level.


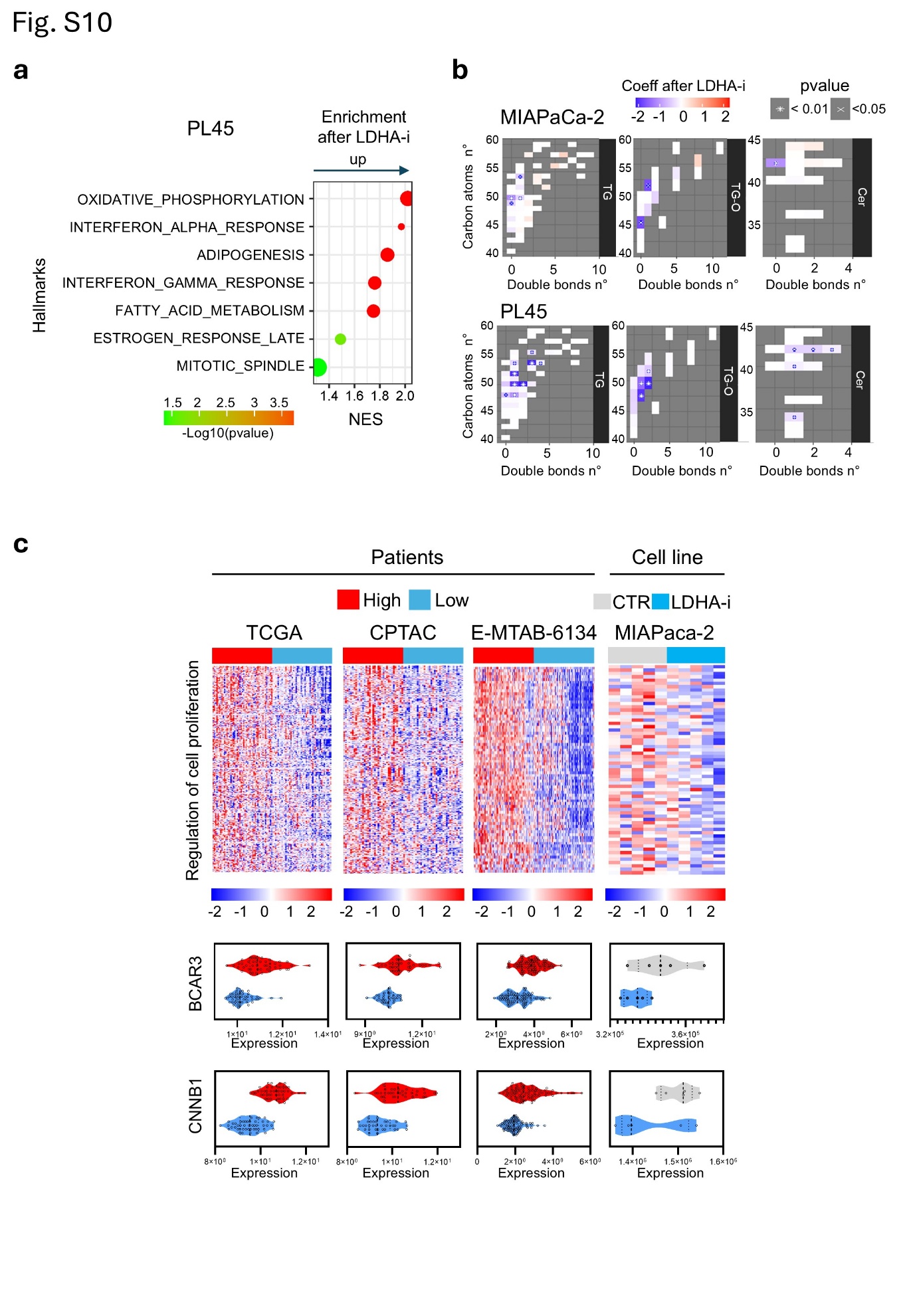


**Supplementary Figure 10: a)** Significantly enriched pathways identified from proteomics data in PL45 cells after LDHA inhibition. The x-axis shows the normalized enrichment score (NES), and bar labels indicate –log₁₀(p-value). **b)** Differential expression analysis of lipid species in MIAPaCa-2 and PL45 cells post-LDHA inhibition. The fold change (coefficient) indicates treatment effect. Statistical significance is denoted as p < 0.01 (*) and p < 0.05 (×). The x- and y-axes represent lipid saturation degree and carbon chain length, respectively. **c)** Heatmaps showing expression of genes involved in cell proliferation in PDAC patients with High and Low glycolytic activity in TCGA, CPTAC, and E-MTAB-6134, and in MIAPaCa-2 cells under basal conditions and following LDHA inhibition. Each dot represents a biological replicate. Data are represented as mean ± SD.


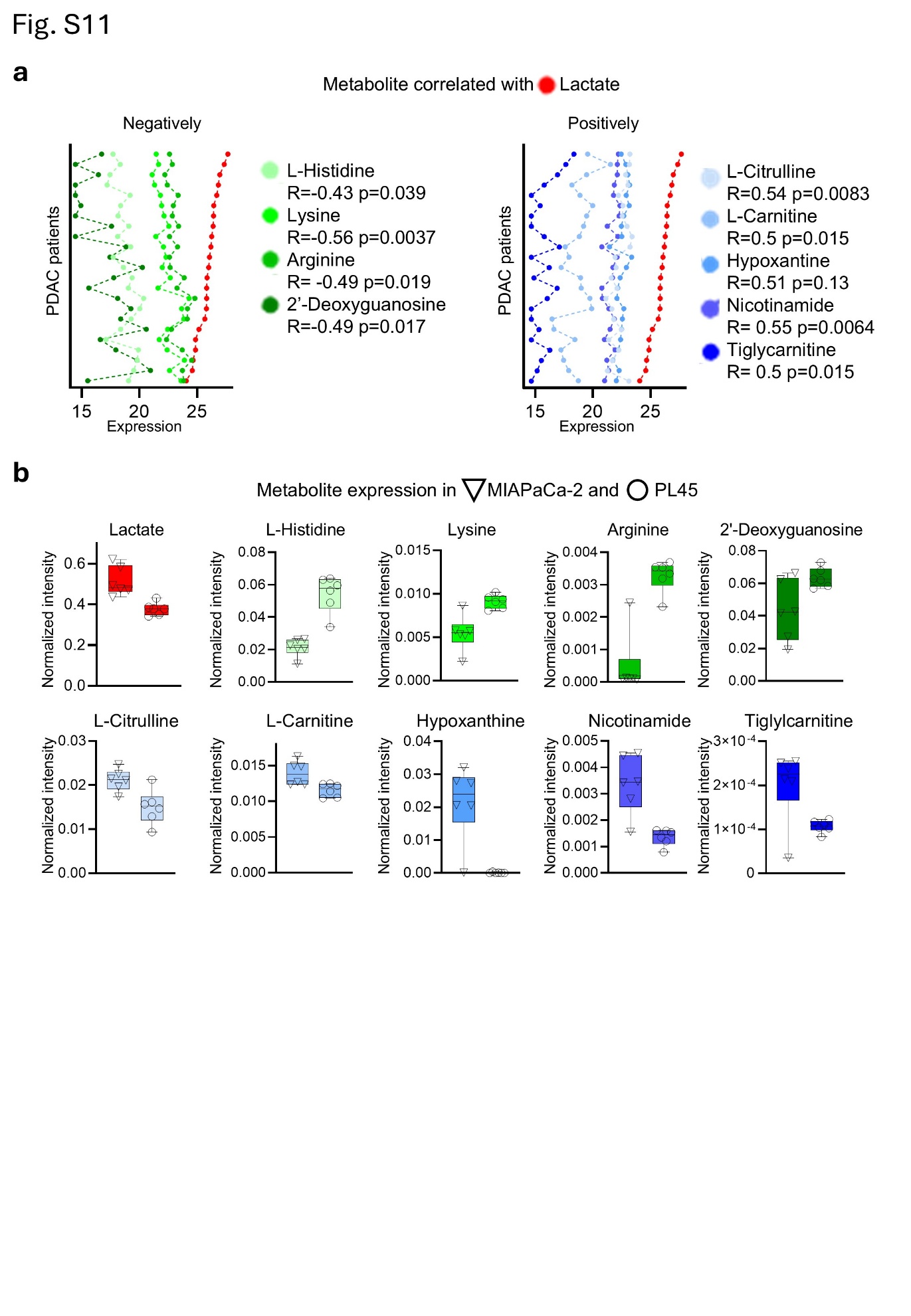


**Supplementary Figure 11: a)** Metabolites significantly correlated to lactate in PDAC patients. **b)** Metabolites up- and downregulated based on lactate amount in MIAPaCa-2 and PL45 cells. Each dot represents a biological replicate (n= 6 per group). Data are represented as mean ± SD.


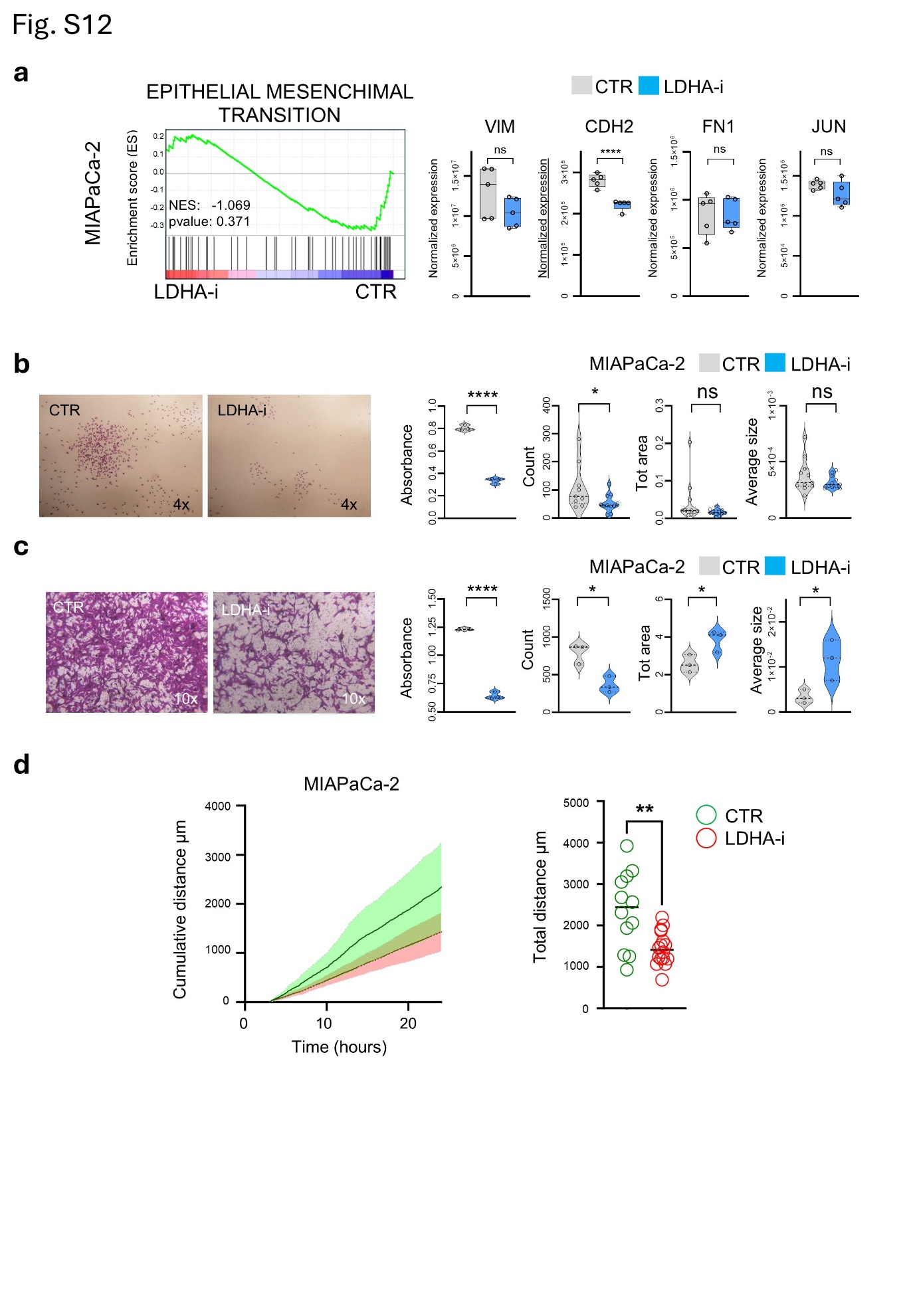


**Supplementary Figure 12: a)** Enrichment plots showing epithelial–mesenchymal transition (EMT) pathway activation in MIAPaCa-2 cells following LDHA inhibition compared to control. On the right side, bar plots display the most significantly enriched genes involved in epithelial to mesenchymal transition in both cell systems. Each dot represents a biological replicate. Data are represented as mean ± SD. Statistical significance (ns=not significant, ****p< 0.0001). **b)** Colony formation assay in MIAPaCa-2 cells treated with LDHA inhibitor (LDHA-i) or control (CTR). Representative images of colony formation are shown. Violin plots quantify absorbance, total colony number, total colony-covered area, and average colony size. Each dot represents a biological replicate. Data are represented as mean ± SD. Statistical significance (ns=not significant, *p< 0.05, ****p< 0.0001). **c)** Transwell migration assay in MIAPaCa-2 cells treated with LDHA-i or CTR. Representative images of migrated cells are shown. Violin plots report absorbance, total number of migrated colonies, total covered area, and average colony size. Each dot represents a biological replicate (n= 3 per group). Data are represented as mean ± SD. Statistical significance (*p< 0.05, ****p< 0.0001). **d)** Cell motility by live-cell imaging for MIAPaCa-2 cells at basal level (green) and treated (red) over 24 h. Cumulative distance traveled by cells is reported as a function of time. Total distance traveled, showed as dotplot, measured as the sum of distances covered by each individual cell between consecutive time points over 24 h. Each dot represents one cell; lines indicate mean ± SD. Statistical significance was determined using unpaired two-tailed Student’st-test (** =p< 0.01, ****p< 0.0001).


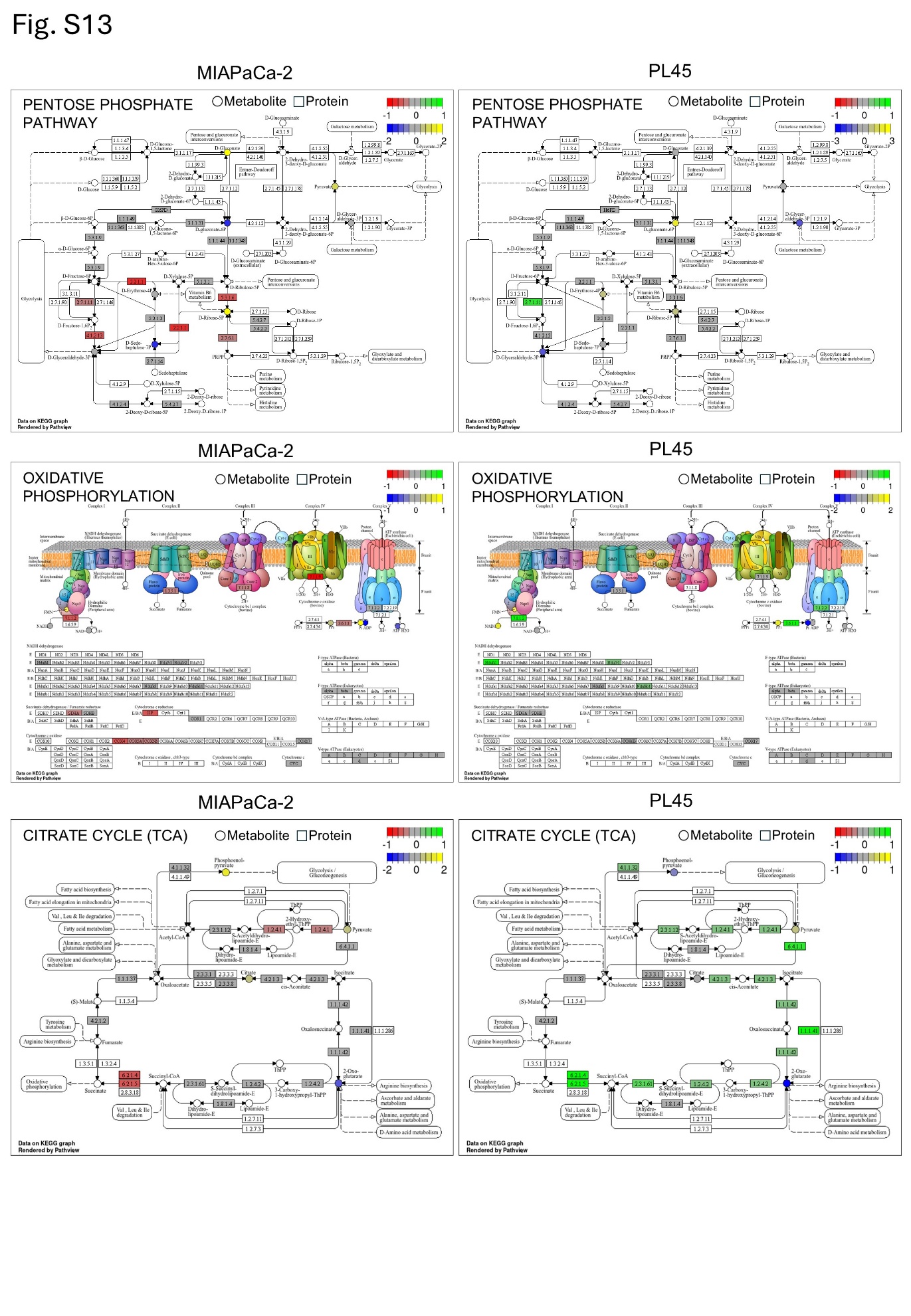


**Supplementary Figure 13:** Visualization of metabolite and protein expression changes mapped onto key metabolic pathways using Pathview in MIAPaCa-2 8left side) and PL45 cells (right side). Log₂ fold changes (treated vs. control) are represented for the Pentose Phosphate Pathway (PPP), Oxidative Phosphorylation (OXPHOS), and Tricarboxylic Acid (TCA) cycle. Color-coded nodes indicate upregulated proteins (green), downregulated proteins (red), upregulated metabolites (yellow), and downregulated metabolites (blue).


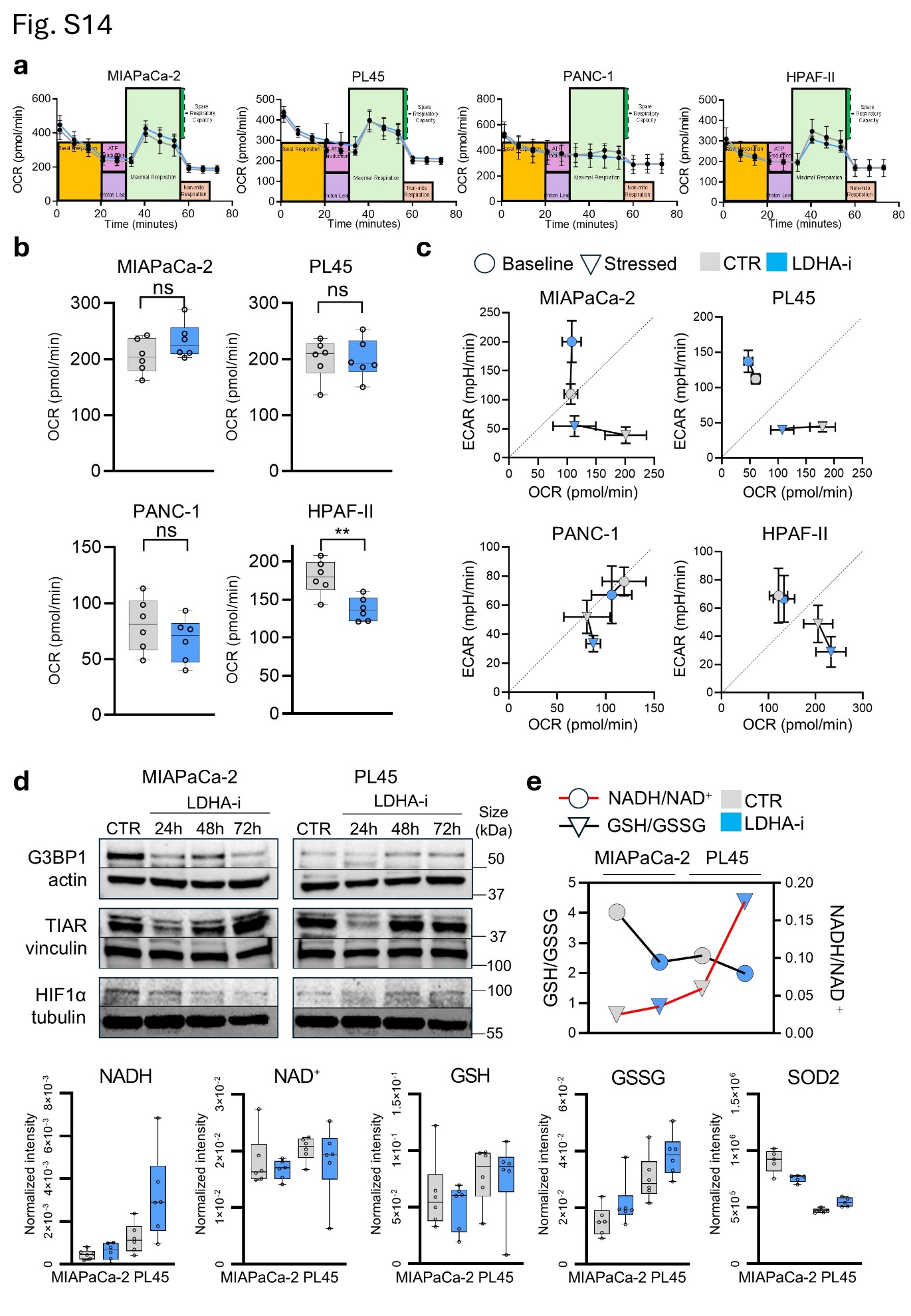


**Supplementary Figure 14: a)** Oxygen consumption rate (OCR) profiles in MIAPaCa-2, PL45, PANC-1, and HPAF-II cells following LDHA inhibition. The assay distinguishes key mitochondrial parameters including ATP production, maximal respiration, spare respiratory capacity, and non-mitochondrial respiration. **b)** Boxplots comparing maximal respiration between control and LDHA-i-treated pancreatic cells. Each dot represents a biological replicate (n= 6 per group). Data are represented as mean ± SD. Statistical significance (ns=not significant, **p< 0.01). **c)** Metabolic phenotype plots showing extracellular acidification rate (ECAR) versus OCR in basal and treated conditions, illustrating shifts in metabolic states upon LDHA inhibition. **d)** Western blot analysis of G3BP1, TIAR1, and HIF-1α expression in MIAPaCa-2 and PL45 cells following LDHA inhibition over a 24, 48, and 72-hours. **e)** Quantification of metabolites and proteins in MIAPaCa-2 and PL45 cells under basal conditions (grey) and after LDHA inhibition (blue), shown as normalized intensity in boxplots. Each dot represents a biological replicate (n= 6 per group). Data are represented as mean ± SD. Graph reports the GSH/GSSG and NADH/NAD⁺ ratios, reflecting redox balance alterations following treatment.

**Table. S1.**

List of metabolic genes from Reactome used as features for single cell data

| AAAS | AMDHD1 | CHST5 | ECI1 | GNG4 | IVD | NAXD | PDHA1 | PRKCA | RPSA | SULT2B1 |
| --- | --- | --- | --- | --- | --- | --- | --- | --- | --- | --- |
| AACS | AMN | CHST6 | ECI2 | GNG5 | IYD | NAXE | PDHA2 | PRKD1 | RRM1 | SULT2B1 |
| AADAC | AMPD1 | CHST7 | ECSIT | GNG7 | KARS1 | NCAN | PDHB | PRKD2 | RRM2 | SULT4A1 |
| AADAT | AMPD2 | CHST9 | EEF1E1 | GNG8 | KCNB1 | NCOA1 | PDHX | PRKD3 | RRM2B | SULT6B1 |
| AANAT | AMPD3 | CHSY1 | EEFSEC | GNGT1 | KCNC2 | NCOA2 | PDK1 | PRKG2 | RTEL1 | SUMF1 |
| AASDHPPT | AMT | CHSY3 | EHHADH | GNGT2 | KCNG2 | NCOA3 | PDK2 | PRODH | RUFY1 | SUMF2 |
| AASS | ANGPTL4 | CIAO1 | ELOVL1 | GNMT | KCNJ11 | NCOA6 | PDK3 | PRODH2 | RXRA | SUMO2 |
| ABCA1 | ANKRD1 | CIAO2B | ELOVL2 | GNPAT | KCNS3 | NCOR1 | PDK4 | PRPS1 | RXRB | SUOX |
| ABCB11 | AOC1 | CIAO3 | ELOVL3 | GNPDA1 | KDSR | NCOR2 | PDP1 | PRPS1L1 | SACM1L | SURF1 |
| ABCB4 | AOC2 | CIAPIN1 | ELOVL4 | GNPDA2 | KERA | NDC1 | PDP2 | PRPS2 | SAMD8 | SYNJ1 |
| ABCB7 | AOC3 | CIDEA | ELOVL5 | GNS | KHK | NDOR1 | PDPR | PRSS1 | SAMHD1 | SYNJ2 |
| ABCC1 | AOX1 | CIDEC | ELOVL6 | GOT1 | KMO | NDST1 | PDSS1 | PRSS3 | SAR1B | SYT5 |
| ABCC2 | APIP | CKB | ELOVL7 | GOT2 | KPNB1 | NDST2 | PDSS2 | PRXL2B | SARDH | TACO1 |
| ABCC3 | APOA1 | CKM | ENO1 | GPAM | KYAT1 | NDST3 | PDXK | PSAP | SARS1 | TALDO1 |
| ABCC5 | APOA2 | CKMT1A | ENO2 | GPAT2 | KYAT3 | NDST4 | PDZD11 | PSAT1 | SAT1 | TAT |
| ABCC8 | APOA4 | CKMT2 | ENO3 | GPAT3 | KYNU | NDUFA1 | PECR | PSMA1 | SBF1 | TAZ |
| ABCD1 | APOA5 | CLOCK | ENOPH1 | GPAT4 | L2HGDH | NDUFA10 | PEMT | PSMA2 | SBF2 | TBL1X |
| ABCD4 | APOB | CLPS | ENPP1 | GPC1 | LALBA | NDUFA11 | PEX11A | PSMA3 | SC5D | TBL1XR1 |
| ABCG2 | APOC2 | CMBL | ENPP2 | GPC2 | LARS1 | NDUFA12 | PFAS | PSMA4 | SCAP | TBXAS1 |
| ABHD10 | APOC3 | CMPK1 | ENPP3 | GPC3 | LBR | NDUFA13 | PFKFB1 | PSMA5 | SCD | TCN1 |
| ABHD14B | APOE | CNDP2 | ENPP6 | GPC4 | LCLAT1 | NDUFA2 | PFKFB2 | PSMA6 | SCD5 | TCN2 |
| ABHD3 | APOM | COA1 | ENPP7 | GPC5 | LDHA | NDUFA3 | PFKFB3 | PSMA7 | SCLY | TDH |
| ABHD4 | APRT | COASY | ENTPD1 | GPC6 | LDHAL6A | NDUFA4 | PFKFB4 | PSMA8 | SCO1 | TDO2 |
| ABHD5 | ARF1 | COMT | ENTPD2 | GPCPD1 | LDHAL6B | NDUFA5 | PFKL | PSMB1 | SCO2 | TECR |
| ABO | ARF3 | COQ10A | ENTPD3 | GPD1 | LDHB | NDUFA6 | PFKM | PSMB10 | SCP2 | TECRL |
| ACAA1 | ARG1 | COQ10B | ENTPD4 | GPD1L | LDHC | NDUFA7 | PFKP | PSMB11 | SCP2 | TGS1 |
| ACAA2 | ARG2 | COQ2 | ENTPD5 | GPD2 | LDLR | NDUFA8 | PGAM1 | PSMB2 | SDC1 | TH |
| ACACA | ARNT | COQ3 | ENTPD6 | GPHN | LGMN | NDUFA9 | PGAM2 | PSMB3 | SDC2 | THEM4 |
| ACACB | ARNT2 | COQ5 | ENTPD7 | GPI | LHB | NDUFAB1 | PGD | PSMB4 | SDC3 | THEM5 |
| ACAD10 | ARNTL | COQ6 | ENTPD8 | GPIHBP1 | LHPP | NDUFAF1 | PGK1 | PSMB5 | SDC4 | THRAP3 |
| ACAD11 | ARSA | COQ7 | EP300 | GPS2 | LIAS | NDUFAF2 | PGK2 | PSMB6 | SDHA | THRSP |
| ACAD8 | ARSB | COQ9 | EPHX1 | GPT | LIPE | NDUFAF3 | PGLS | PSMB7 | SDHB | THTPA |
| ACAD9 | ARSD | COX10 | EPHX2 | GPT2 | LIPH | NDUFAF4 | PGM1 | PSMB8 | SDHC | TIAM2 |
| ACADL | ARSF | COX11 | EPM2A | GPX1 | LIPI | NDUFAF5 | PGM2 | PSMB9 | SDHD | TIMMDC1 |
| ACADM | ARSG | COX14 | EPRS1 | GPX2 | LIPT1 | NDUFAF6 | PGM2L1 | PSMC1 | SDS | TK1 |
| ACADS | ARSH | COX15 | ERCC2 | GPX4 | LIPT2 | NDUFAF7 | PGP | PSMC2 | SDSL | TK2 |
| ACADSB | ARSI | COX16 | ESD | GPX4 | LMBRD1 | NDUFB1 | PGS1 | PSMC3 | SEC13 | TKFC |
| ACADVL | ARSJ | COX18 | ESRRA | GRHL1 | LPCAT1 | NDUFB10 | PHGDH | PSMC4 | SEC23A | TKT |
| ACAN | ARSK | COX19 | ESYT1 | GRHPR | LPCAT2 | NDUFB11 | PHKA1 | PSMC5 | SEC24A | TM7SF2 |
| ACAT1 | ARSL | COX20 | ESYT2 | GSR | LPCAT3 | NDUFB2 | PHKA2 | PSMC6 | SEC24B | TMEM126B |
| ACAT2 | ARV1 | COX4I1 | ESYT3 | GSS | LPCAT4 | NDUFB3 | PHKB | PSMD1 | SEC24C | TMEM186 |
| ACBD4 | AS3MT | COX5A | ETFA | GSTA1 | LPGAT1 | NDUFB4 | PHKG1 | PSMD10 | SEC24D | TMEM86B |
| ACBD5 | ASAH1 | COX5B | ETFB | GSTA2 | LPIN1 | NDUFB5 | PHKG2 | PSMD11 | SECISBP2 | TMLHE |
| ACBD6 | ASAH2 | COX6A1 | ETFDH | GSTA3 | LPIN2 | NDUFB6 | PHOSPHO1 | PSMD12 | SEH1L | TNFAIP8 |
| ACBD7 | ASL | COX6B1 | ETHE1 | GSTA4 | LPIN3 | NDUFB7 | PHYH | PSMD13 | SEH1L | TNFAIP8L1 |
| ACER1 | ASMT | COX6C | ETNK1 | GSTA5 | LPL | NDUFB8 | PHYKPL | PSMD14 | SELENOI | TNFAIP8L2 |
| ACER2 | ASNS | COX7A2L | ETNK2 | GSTK1 | LRAT | NDUFB9 | PI4K2A | PSMD2 | SEM1 | TNFAIP8L3 |
| ACER3 | ASPA | COX7B | ETNPPL | GSTM1 | LRP1 | NDUFC1 | PI4K2B | PSMD3 | SEPHS2 | TNFRSF21 |
| ACHE | ASPG | COX7C | EXT1 | GSTM2 | LRP10 | NDUFC2 | PI4KA | PSMD4 | SEPSECS | TPH1 |
| ACLY | ASRGL1 | COX8A | EXT2 | GSTM3 | LRP12 | NDUFS1 | PI4KB | PSMD5 | SERINC1 | TPH2 |
| ACMSD | ASS1 | CPNE1 | FA2H | GSTM4 | LRP2 | NDUFS2 | PIAS4 | PSMD6 | SERINC2 | TPI1 |
| ACO2 | ATIC | CPNE3 | FAAH | GSTM5 | LRP8 | NDUFS3 | PIK3C2A | PSMD7 | SERINC3 | TPK1 |
| ACOT1 | ATP5F1A | CPNE6 | FAAH2 | GSTO1 | LRPPRC | NDUFS4 | PIK3C2B | PSMD8 | SERINC4 | TPMT |
| ACOT11 | ATP5F1B | CPNE7 | FABP1 | GSTO2 | LSS | NDUFS5 | PIK3C2G | PSMD9 | SERINC5 | TPO |
| ACOT12 | ATP5F1C | CPOX | FABP12 | GSTP1 | LTA4H | NDUFS6 | PIK3C3 | PSME1 | SERPINA6 | TPR |
| ACOT13 | ATP5F1D | CPS1 | FABP2 | GSTT1 | LTC4S | NDUFS7 | PIK3CA | PSME2 | SGMS1 | TPST1 |
| ACOT2 | ATP5F1E | CPT1A | FABP3 | GSTT2 | LUM | NDUFS8 | PIK3CB | PSME3 | SGMS2 | TPST2 |
| ACOT4 | ATP5MC1 | CPT1B | FABP4 | GSTT2B | LYPLA1 | NDUFV1 | PIK3CD | PSME4 | SGPL1 | TPTE |
| ACOT6 | ATP5MC2 | CPT2 | FABP5 | GSTZ1 | LYRM4 | NDUFV2 | PIK3CG | PSMF1 | SGPP1 | TPTE2 |
| ACOT7 | ATP5MC3 | CPTP | FABP6 | GUK1 | LYVE1 | NDUFV3 | PIK3R1 | PSPH | SGPP2 | TRAP1 |
| ACOT7L | ATP5ME | CRAT | FABP7 | GUSB | MAN2B1 | NEU1 | PIK3R2 | PSTK | SGSH | TRIB3 |
| ACOT8 | ATP5MF | CREBBP | FABP9 | GUSB | MAN2B2 | NEU2 | PIK3R3 | PTDSS1 | SHMT1 | TRMT112 |
| ACOT9 | ATP5MG | CRLS1 | FADS1 | GYG1 | MAN2C1 | NEU3 | PIK3R4 | PTDSS2 | SHMT2 | TSHB |
| ACOX1 | ATP5PB | CROT | FADS2 | GYG2 | MANBA | NEU4 | PIK3R5 | PTEN | SHPK | TSPO |
| ACOX1 | ATP5PD | CRYL1 | FAH | GYS1 | MAOA | NFS1 | PIK3R6 | PTGDS | SIN3A | TSPOAP1 |
| ACOX2 | ATP5PF | CRYM | FAHD1 | GYS2 | MAOB | NFS1 | PIKFYVE | PTGES | SIN3B | TST |
| ACOX3 | ATP5PO | CS | FAM120B | HAAO | MAPKAPK2 | NFYA | PIP4K2A | PTGES2 | SLC10A1 | TSTD1 |
| ACOXL | AUH | CSAD | FAR1 | HACD1 | MARCKS | NFYB | PIP4K2B | PTGES3 | SLC10A2 | TTPA |
| ACP5 | AWAT1 | CSGALNACT1 | FAR2 | HACD2 | MARS1 | NFYC | PIP4K2C | PTGIS | SLC16A1 | TTR |
| ACP6 | AWAT2 | CSGALNACT2 | FASN | HACD3 | MAT1A | NHLRC1 | PIP4P1 | PTGR1 | SLC16A3 | TXN |
| ACSBG1 | AZIN1 | CSNK1G2 | FAU | HACD4 | MAT2A | NME1 | PIP5K1A | PTGR2 | SLC16A8 | TXN2 |
| ACSBG2 | AZIN2 | CSNK2A1 | FBP1 | HACL1 | MAT2B | NME2 | PIP5K1B | PTGS1 | SLC19A1 | TXNRD1 |
| ACSF2 | B3GALNT1 | CSNK2A2 | FBP2 | HADH | MBOAT1 | NME2P1 | PIP5K1C | PTGS2 | SLC19A2 | TYMP |
| ACSF3 | B3GALT1 | CSNK2B | FDFT1 | HADHA | MBOAT2 | NME3 | PIPOX | PTPMT1 | SLC19A3 | TYMS |
| ACSL1 | B3GALT2 | CSPG4 | FDPS | HADHB | MBOAT7 | NME4 | PISD | PTPN13 | SLC22A13 | TYR |
| ACSL3 | B3GALT4 | CSPG5 | FDX1 | HAGH | MBTPS1 | NMNAT1 | PITPNB | PTS | SLC22A5 | TYRP1 |
| ACSL4 | B3GALT5 | CTH | FDX2 | HAL | MBTPS2 | NMNAT2 | PITPNM1 | PUDP | SLC23A1 | UBA52 |
| ACSL5 | B3GALT6 | CTPS1 | FDXR | HAO1 | MCAT | NMNAT3 | PITPNM2 | PXMP2 | SLC23A2 | UBB |
| ACSL6 | B3GAT1 | CTPS2 | FECH | HAO2 | MCCC1 | NMRAL1 | PITPNM3 | PYCR1 | SLC25A1 | UBC |
| ACSM1 | B3GAT2 | CTRB1 | FFAR1 | HAS1 | MCCC2 | NMRK1 | PKLR | PYCR2 | SLC25A10 | UBE2I |
| ACSM2A | B3GAT3 | CTRB2 | FH | HAS2 | MCEE | NMRK2 | PKLR | PYCR3 | SLC25A11 | UBIAD1 |
| ACSM2B | B3GNT2 | CTRC | FHL2 | HAS3 | MDH1 | NNMT | PKM | PYGB | SLC25A12 | UCK1 |
| ACSM3 | B3GNT3 | CTSA | FIG4 | HDAC3 | MDH2 | NNT | PKM | PYGL | SLC25A13 | UCK2 |
| ACSM4 | B3GNT4 | CUBN | FITM1 | HDC | ME1 | NOS3 | PLA1A | PYGM | SLC25A14 | UCKL1 |
| ACSM5 | B3GNT7 | CYB5A | FITM2 | HELZ2 | ME2 | NOSIP | PLA2G10 | QARS1 | SLC25A15 | UCP1 |
| ACSM6 | B4GALNT1 | CYB5B | FLAD1 | HEXA | ME3 | NOSTRIN | PLA2G12A | QDPR | SLC25A16 | UCP2 |
| ACSS1 | B4GALNT2 | CYB5R3 | FLVCR1 | HEXB | MECR | NPAS2 | PLA2G15 | QPRT | SLC25A17 | UCP3 |
| ACSS2 | B4GALT1 | CYC1 | FLVCR1 | HGD | MED1 | NQO1 | PLA2G1B | RAB14 | SLC25A19 | UGCG |
| ACSS3 | B4GALT2 | CYCS | FMO1 | HGSNAT | MED10 | NQO2 | PLA2G2A | RAB4A | SLC25A2 | UGDH |
| ACY1 | B4GALT3 | CYGB | FMO2 | HIBADH | MED11 | NR1D1 | PLA2G2D | RAB5A | SLC25A20 | UGP2 |
| ACY3 | B4GALT4 | CYP11A1 | FMO3 | HIBCH | MED12 | NR1H2 | PLA2G2E | RAE1 | SLC25A21 | UGT1A1 |
| ADA | B4GALT5 | CYP11B1 | FMOD | HILPDA | MED13 | NR1H3 | PLA2G2F | RAN | SLC25A27 | UGT1A10 |
| ADAL | B4GALT6 | CYP11B2 | FOLH1 | HK1 | MED13L | NR1H4 | PLA2G3 | RANBP2 | SLC25A28 | UGT1A3 |
| ADCY1 | B4GALT7 | CYP17A1 | FOLH1B | HK2 | MED14 | NRF1 | PLA2G4A | RAP1A | SLC25A32 | UGT1A4 |
| ADCY2 | B4GAT1 | CYP19A1 | FOLR2 | HK3 | MED15 | NSDHL | PLA2G4B | RAPGEF3 | SLC25A37 | UGT1A5 |
| ADCY3 | BAAT | CYP1A1 | FPGS | HLCS | MED16 | NT5C | PLA2G4C | RAPGEF4 | SLC25A44 | UGT1A6 |
| ADCY4 | BBOX1 | CYP1A2 | FPGS | HMBS | MED17 | NT5C1A | PLA2G4D | RARS1 | SLC26A1 | UGT1A7 |
| ADCY5 | BCAN | CYP1B1 | FTCD | HMGCL | MED18 | NT5C1B | PLA2G4E | RBKS | SLC26A2 | UGT1A8 |
| ADCY6 | BCAT1 | CYP21A2 | FUT1 | HMGCLL1 | MED19 | NT5C2 | PLA2G4F | RBP1 | SLC27A1 | UGT1A9 |
| ADCY7 | BCAT2 | CYP24A1 | FUT10 | HMGCR | MED20 | NT5C3A | PLA2G5 | RBP2 | SLC27A2 | UGT2A1 |
| ADCY8 | BCHE | CYP26A1 | FUT11 | HMGCR | MED21 | NT5E | PLA2G6 | RBP4 | SLC27A3 | UGT2A2 |
| ADCY9 | BCKDHA | CYP26B1 | FUT2 | HMGCS1 | MED22 | NT5M | PLA2R1 | RDH11 | SLC27A5 | UGT2A3 |
| ADH1A | BCKDHB | CYP26C1 | FUT3 | HMGCS2 | MED23 | NUBP1 | PLAAT1 | RETSAT | SLC2A1 | UGT2B10 |
| ADH1B | BCKDK | CYP27A1 | FUT4 | HMMR | MED24 | NUBP2 | PLAAT2 | RFK | SLC2A2 | UGT2B11 |
| ADH1C | BCO1 | CYP27B1 | FUT5 | HMOX1 | MED25 | NUBPL | PLAAT3 | RGL1 | SLC2A3 | UGT2B15 |
| ADH4 | BCO2 | CYP2A13 | FUT6 | HMOX2 | MED26 | NUDT1 | PLAAT4 | RHCE | SLC35B2 | UGT2B17 |
| ADH5 | BDH1 | CYP2A6 | FUT7 | HNMT | MED27 | NUDT1 | PLAAT5 | RHD | SLC35B3 | UGT2B28 |
| ADH6 | BDH2 | CYP2A7 | FUT9 | HOGA1 | MED28 | NUDT1 | PLB1 | RIDA | SLC35D1 | UGT2B4 |
| ADH7 | BGN | CYP2B6 | FXN | HPD | MED29 | NUDT1 | PLBD1 | RIMKLA | SLC35D2 | UGT2B7 |
| ADHFE1 | BHMT | CYP2C18 | G0S2 | HPGD | MED30 | NUDT10 | PLCB1 | RIMKLB | SLC36A4 | UGT3A1 |
| ADI1 | BHMT2 | CYP2C19 | G6PC1 | HPGDS | MED31 | NUDT11 | PLCB2 | RNLS | SLC37A1 | UGT3A2 |
| ADIPOQ | BLVRA | CYP2C8 | G6PC2 | HPRT1 | MED4 | NUDT12 | PLCB3 | RORA | SLC37A2 | UGT8 |
| ADIPOR1 | BLVRB | CYP2C9 | G6PC3 | HPSE | MED6 | NUDT13 | PLCB4 | RPE | SLC37A4 | UMPS |
| ADIPOR2 | BMX | CYP2D6 | G6PD | HPSE2 | MED7 | NUDT15 | PLCD1 | RPEL1 | SLC3A2 | UPB1 |
| ADK | BPGM | CYP2E1 | GAA | HS2ST1 | MED8 | NUDT16 | PLCD3 | RPIA | SLC44A1 | UPP1 |
| ADO | BPHL | CYP2F1 | GADL1 | HS3ST1 | MED9 | NUDT18 | PLCD4 | RPL10 | SLC44A2 | UPP2 |
| ADPGK | BPNT1 | CYP2J2 | GALC | HS3ST2 | MFSD2A | NUDT19 | PLCE1 | RPL10A | SLC44A3 | UQCR10 |
| ADPRM | BPNT2 | CYP2R1 | GALE | HS3ST3A1 | MGLL | NUDT3 | PLCG1 | RPL10L | SLC44A4 | UQCR11 |
| ADRA2A | BRIP1 | CYP2S1 | GALK1 | HS3ST3B1 | MGST1 | NUDT4 | PLCG2 | RPL11 | SLC44A5 | UQCRB |
| ADRA2C | BSG | CYP2U1 | GALNS | HS3ST4 | MGST2 | NUDT5 | PLCH1 | RPL12 | SLC45A2 | UQCRC1 |
| ADSL | BST1 | CYP2W1 | GALT | HS3ST5 | MGST3 | NUDT7 | PLCH2 | RPL13 | SLC46A1 | UQCRC2 |
| ADSS1 | BTD | CYP39A1 | GAMT | HS3ST6 | MID1IP1 | NUDT9 | PLCZ1 | RPL13A | SLC51A | UQCRFS1 |
| ADSS2 | CA1 | CYP3A4 | GAPDH | HS6ST1 | MIGA1 | NUP107 | PLD1 | RPL14 | SLC51B | UQCRH |
| AFMID | CA12 | CYP3A43 | GAPDHS | HS6ST2 | MIGA2 | NUP133 | PLD2 | RPL15 | SLC52A1 | UQCRQ |
| AGK | CA13 | CYP3A5 | GART | HS6ST3 | MINPP1 | NUP153 | PLD3 | RPL17 | SLC52A2 | UROC1 |
| AGL | CA14 | CYP3A7 | GATM | HSCB | MIOX | NUP155 | PLD4 | RPL18 | SLC52A3 | UROD |
| AGMAT | CA2 | CYP46A1 | GBA | HSD11B1 | MLX | NUP160 | PLD6 | RPL18A | SLC5A5 | UROS |
| AGMO | CA3 | CYP4A11 | GBA2 | HSD11B2 | MLXIPL | NUP188 | PLEKHA1 | RPL19 | SLC5A6 | UST |
| AGPAT1 | CA4 | CYP4A22 | GBA3 | HSD17B1 | MLYCD | NUP205 | PLEKHA2 | RPL21 | SLC5A8 | VAC14 |
| AGPAT2 | CA5A | CYP4B1 | GBE1 | HSD17B10 | MMAA | NUP210 | PLEKHA3 | RPL22 | SLC6A11 | VAMP2 |
| AGPAT3 | CA5B | CYP4F11 | GC | HSD17B11 | MMAB | NUP214 | PLEKHA4 | RPL22L1 | SLC6A12 | VAPA |
| AGPAT4 | CA6 | CYP4F12 | GCAT | HSD17B12 | MMACHC | NUP35 | PLEKHA5 | RPL23 | SLC6A7 | VAPB |
| AGPAT5 | CA7 | CYP4F2 | GCDH | HSD17B13 | MMADHC | NUP37 | PLEKHA6 | RPL23A | SLC6A8 | VCAN |
| AGPS | CA9 | CYP4F22 | GCG | HSD17B14 | MMS19 | NUP42 | PLEKHA8 | RPL24 | SLC7A5 | VDAC1 |
| AGRN | CACNA1A | CYP4F3 | GCH1 | HSD17B2 | MMUT | NUP43 | PLIN1 | RPL26 | SLC9A1 | VDR |
| AGT | CACNA1C | CYP4F8 | GCHFR | HSD17B3 | MOCOS | NUP50 | PLIN2 | RPL26L1 | SLCO1A2 | VKORC1 |
| AGXT | CACNA1D | CYP4V2 | GCK | HSD17B4 | MOCS1 | NUP54 | PLIN3 | RPL27 | SLCO1B1 | VKORC1L1 |
| AGXT2 | CACNA1E | CYP51A1 | GCKR | HSD17B7 | MOCS1 | NUP58 | PLPP1 | RPL27A | SLCO1B3 | VNN1 |
| AHCY | CACNA2D2 | CYP7A1 | GCLC | HSD17B8 | MOCS2 | NUP58 | PLPP2 | RPL28 | SLCO2B1 | VNN2 |
| AHCYL1 | CACNB2 | CYP7B1 | GCLM | HSD3B1 | MOCS2 | NUP62 | PLPP3 | RPL29 | SMARCD3 | WASL |
| AHR | CACNB3 | CYP8B1 | GCSH | HSD3B2 | MOCS3 | NUP85 | PLPP6 | RPL3 | SMOX | XDH |
| AHRR | CAD | CYSLTR2 | GDA | HSD3B7 | MOGAT1 | NUP88 | PM20D1 | RPL30 | SMPD1 | XYLB |
| AIMP1 | CALM1 | D2HGDH | GDE1 | HSP90AA1 | MOGAT2 | NUP93 | PMVK | RPL31 | SMPD2 | XYLT1 |
| AIMP2 | CARM1 | DAO | GDPD1 | HSP90AB1 | MOGAT3 | NUP98 | PNLIP | RPL32 | SMPD3 | XYLT2 |
| AIP | CARNMT1 | DARS1 | GDPD3 | HSPG2 | MORC2 | NUP98 | PNMT | RPL34 | SMPD4 | ZDHHC21 |
| AK1 | CARNS1 | DBH | GDPD5 | HTD2 | MPC1 | NUP98 | PNP | RPL35 | SMS |  |
| AK2 | CAV1 | DBI | GGCT | HYAL1 | MPC2 | OAT | PNPLA2 | RPL35A | SNAP25 |  |
| AK4 | CBLIF | DBT | GGPS1 | HYAL2 | MPST | OAZ1 | PNPLA3 | RPL36 | SORD |  |
| AK5 | CBR1 | DCK | GGT1 | HYAL3 | MRI1 | OAZ2 | PNPLA4 | RPL36A | SP1 |  |
| AK6 | CBR3 | DCN | GGT3P | HYKK | MSMO1 | OAZ3 | PNPLA5 | RPL36AL | SPHK1 |  |
| AK7 | CBR4 | DCT | GGT5 | IARS1 | MTAP | OCA2 | PNPLA6 | RPL37 | SPHK2 |  |
| AK8 | CBS | DCTD | GGT6 | IDH1 | MTARC1 | OCRL | PNPLA7 | RPL37A | SPNS2 |  |
| AK9 | CBSL | DCTPP1 | GGT7 | IDH2 | MTARC2 | ODC1 | PNPLA8 | RPL38 | SPR |  |
| AKAP5 | CCNC | DCXR | GK | IDH3A | MT-ATP6 | OGDH | PNPO | RPL39 | SPTLC1 |  |
| AKR1A1 | CD320 | DDAH1 | GK2 | IDH3B | MT-ATP8 | OGN | PODXL2 | RPL39L | SPTLC2 |  |
| AKR1B1 | CD36 | DDAH2 | GK3P | IDH3G | MT-CO1 | OLAH | POLD1 | RPL3L | SPTLC3 |  |
| AKR1B10 | CD38 | DDC | GLA | IDI1 | MT-CO2 | OMD | POM121 | RPL4 | SPTSSA |  |
| AKR1B15 | CD44 | DDHD1 | GLB1 | IDI2 | MT-CO3 | OPLAH | POM121C | RPL41 | SPTSSB |  |
| AKR1C1 | CDA | DDHD2 | GLB1L | IDO1 | MT-CYB | ORMDL1 | POMC | RPL5 | SQLE |  |
| AKR1C2 | CDIPT | DDO | GLCE | IDO2 | MTF1 | ORMDL2 | PON1 | RPL6 | SQOR |  |
| AKR1C3 | CDK19 | DECR1 | GLDC | IDS | MTHFD1 | ORMDL3 | PON2 | RPL7 | SRD5A1 |  |
| AKR1C4 | CDK8 | DECR2 | GLIPR1 | IDUA | MTHFD1L | OSBP | PON3 | RPL7A | SRD5A2 |  |
| AKR1D1 | CDO1 | DEGS1 | GLO1 | IL4I1 | MTHFD2 | OSBPL10 | POR | RPL8 | SRD5A3 |  |
| AKR7A2 | CDS1 | DEGS2 | GLP1R | IMPA1 | MTHFD2L | OSBPL1A | PPA1 | RPL9 | SREBF1 |  |
| AKR7A3 | CDS2 | DERA | GLRX | IMPA2 | MTHFR | OSBPL2 | PPA2 | RPLP0 | SREBF1 |  |
| AKR7L | CEMIP | DGAT1 | GLRX5 | IMPDH1 | MTHFS | OSBPL3 | PPARA | RPLP1 | SREBF1 |  |
| AKT1 | CEPT1 | DGAT2 | GLS | IMPDH2 | MTM1 | OSBPL5 | PPARD | RPLP2 | SREBF2 |  |
| ALAD | CERK | DGAT2L6 | GLS2 | INMT | MTMR1 | OSBPL6 | PPARG | RPS10 | SRM |  |
| ALAS1 | CERS1 | DGAT2L7P | GLTP | INPP1 | MTMR10 | OSBPL7 | PPARGC1A | RPS11 | SRR |  |
| ALAS2 | CERS2 | DGUOK | GLUD1 | INPP4A | MTMR12 | OSBPL8 | PPARGC1B | RPS12 | ST3GAL1 |  |
| ALB | CERS3 | DHCR24 | GLUD2 | INPP4B | MTMR14 | OSBPL9 | PPAT | RPS13 | ST3GAL2 |  |
| ALDH18A1 | CERS4 | DHCR7 | GLUL | INPP5A | MTMR2 | OTC | PPCDC | RPS14 | ST3GAL3 |  |
| ALDH18A1 | CERS5 | DHFR | GLYAT | INPP5B | MTMR3 | OXCT1 | PPCS | RPS15 | ST3GAL4 |  |
| ALDH1A1 | CERS6 | DHFR2 | GLYATL1 | INPP5D | MTMR4 | OXCT2 | PPIP5K1 | RPS15A | ST3GAL6 |  |
| ALDH1B1 | CERT1 | DHODH | GLYATL2 | INPP5E | MTMR6 | PAH | PPIP5K2 | RPS16 | ST6GALNAC6 | |
| ALDH1L1 | CES1 | DHRS7B | GLYATL3 | INPP5F | MTMR7 | PAICS | PPM1K | RPS17 | STAB2 |  |
| ALDH1L2 | CES2 | DHTKD1 | GLYCTK | INPP5J | MTMR8 | PANK1 | PPM1L | RPS18 | STAR |  |
| ALDH2 | CES3 | DIO1 | GM2A | INPP5K | MTMR9 | PANK2 | PPOX | RPS19 | STARD10 |  |
| ALDH3A1 | CGA | DIO2 | GMPR | INPPL1 | MT-ND1 | PANK3 | PPP1CA | RPS2 | STARD3 |  |
| ALDH3A2 | CH25H | DIO3 | GMPR2 | INS | MT-ND2 | PANK4 | PPP1CB | RPS20 | STARD3NL |  |
| ALDH3A2 | CHAC1 | DLAT | GMPS | INSIG1 | MT-ND3 | PAOX | PPP1CC | RPS21 | STARD4 |  |
| ALDH3B1 | CHAC2 | DLD | GNA11 | INSIG2 | MT-ND4 | PAPSS1 | PPP1R3C | RPS23 | STARD5 |  |
| ALDH3B2 | CHAT | DLST | GNA14 | IP6K1 | MT-ND5 | PAPSS2 | PPP2CA | RPS24 | STARD6 |  |
| ALDH4A1 | CHD9 | DMAC2L | GNA15 | IP6K2 | MT-ND6 | PARP10 | PPP2CB | RPS25 | STARD7 |  |
| ALDH6A1 | CHDH | DMGDH | GNAI1 | IP6K3 | MTR | PARP14 | PPP2R1A | RPS26 | STK11 |  |
| ALDH7A1 | CHKA | DNM2 | GNAI2 | IPMK | MTRR | PARP16 | PPP2R1B | RPS27 | STS |  |
| ALDH9A1 | CHKB | DNPH1 | GNAQ | IPPK | MVD | PARP4 | PPP2R5D | RPS27A | STX1A |  |
| ALDOA | CHP1 | DPEP1 | GNAS | IQGAP1 | MVK | PARP6 | PPT1 | RPS27L | STXBP1 |  |
| ALDOB | CHPF | DPEP2 | GNAS | ISCA1 | N6AMT1 | PARP8 | PPT2 | RPS28 | SUCLA2 |  |
| ALDOC | CHPF2 | DPEP3 | GNB1 | ISCA2 | NAALAD2 | PARP9 | PRELP | RPS29 | SUCLG1 |  |
| ALOX12 | CHPT1 | DPYD | GNB2 | ISCU | NADK | PC | PRKAA2 | RPS3 | SUCLG2 |  |
| ALOX12B | CHRM3 | DPYS | GNB3 | ISYNA1 | NADK2 | PCBD1 | PRKAB2 | RPS3A | SULT1A1 |  |
| ALOX15 | CHST1 | DSE | GNB4 | ITPA | NADSYN1 | PCCA | PRKACA | RPS4X | SULT1A2 |  |
| ALOX15B | CHST11 | DSEL | GNB5 | ITPK1 | NAGLU | PCCB | PRKACB | RPS4Y1 | SULT1A3 |  |
| ALOX5 | CHST12 | DTYMK | GNG10 | ITPKA | NAGS | PCK1 | PRKACG | RPS4Y2 | SULT1A4 |  |
| ALOX5AP | CHST13 | DUOX1 | GNG11 | ITPKB | NAMPT | PCK2 | PRKAG2 | RPS5 | SULT1B1 |  |
| ALOXE3 | CHST14 | DUOX2 | GNG12 | ITPKC | NAPRT | PCTP | PRKAR1A | RPS6 | SULT1C2 |  |
| ALPI | CHST15 | DUT | GNG13 | ITPR1 | NAT1 | PCYT1A | PRKAR1B | RPS7 | SULT1C4 |  |
| AMACR | CHST2 | EBP | GNG2 | ITPR2 | NAT2 | PCYT1B | PRKAR2A | RPS8 | SULT1E1 |  |
| AMD1 | CHST3 | ECHS1 | GNG3 | ITPR3 | NAT8L | PCYT2 | PRKAR2B | RPS9 | SULT2A1 |  |

**Table. S2.**

clinical information of PDAC patients processed for Spatial Transcriptomics

| **ID** | **Diagnosis** | **Grade** | **T** | **N** | **Stage** |
| --- | --- | --- | --- | --- | --- |
| 1 | Pancreatic Ductal AdenoCarcinoma PDAC | 2 | 3 | 1 | IIB |
| 2 | Pancreatic Ductal AdenoCarcinoma PDAC | 2 | 3 | 1 | IIB |
| 3 | Pancreatic Ductal AdenoCarcinoma PDAC | 2 | 3 | 1 | IIB |
| 4 | Pancreatic Ductal AdenoCarcinoma PDAC | 2 | 3 | 0 | IIA |
| 5 | Pancreatic Ductal AdenoCarcinoma PDAC | 3 | 2 | 1 | IIB |
| 6 | Pancreatic Ductal AdenoCarcinoma PDAC | 2 | 2 | 0 | IB |

**Table. S3.**

Cell types identified in tumor regions from deconvoluted Spatial Transcriptomic data

| **ID patient** | 1 | 1 | 2 | 2 | 3 | 3 | 4 | 4 | 5 | 5 | 6 | 6 |
| --- | --- | --- | --- | --- | --- | --- | --- | --- | --- | --- | --- | --- |
| **AOI** | 1 | 2 | 1 | 2 | 1 | 2 | 1 | 2 | 1 | 2 | 1 | 2 |
| **Marker** | PanCK+ | PanCK+ | PanCK+ | PanCK+ | PanCK+ | PanCK+ | PanCK+ | PanCK+ | PanCK+ | PanCK+ | PanCK+ | PanCK+ |
| **Cell Type** | Tumor | Tumor | Tumor | Tumor | Tumor | Tumor | Tumor | Tumor | Tumor | Tumor | Tumor | Tumor |
| **B memory** | 0 | 0.035658 | 0.081415 | 0 | 0 | 0.160114 | 0.011512 | 0.035161 | 0 | 0 | 0 | 0.030468 |
| **B naive** | 0.087498 | 0.009709 | 0 | 0.034455 | 0.011236 | 0 | 0 | 0 | 0.038971 | 0.169833 | 0 | 0 |
| **Endothelial cells** | 0.183063 | 0.11162 | 0.231287 | 0.141336 | 0.013445 | 0.043703 | 0.153509 | 0 | 0.349129 | 0.257395 | 0.29516 | 0.370881 |
| **Fibroblasts** | 0.538398 | 0.412421 | 0.194468 | 0.157652 | 0.03274 | 0.141522 | 0.202537 | 0.354603 | 0.381553 | 0.100868 | 0.508328 | 0.082983 |
| **Macrophages** | 0.010719 | 0.033013 | 0.061413 | 0.209577 | 0.028918 | 0.083332 | 0.021605 | 0.062119 | 0.062725 | 0.14775 | 0 | 0 |
| **Mast cells** | 0.00071 | 0.004578 | 0.022394 | 0.004117 | 0.011946 | 0 | 0.003315 | 0.035962 | 0.004724 | 0.021685 | 0 | 0.009467 |
| **mDCs** | 0.007082 | 0.004141 | 0.023791 | 0.040377 | 0 | 0 | 0 | 0 | 0 | 0.003364 | 0.079448 | 0.018978 |
| **Monocytes C** | 0 | 0.029938 | 0 | 0 | 0 | 0.028035 | 0.041888 | 0.086771 | 0.023094 | 0.000803 | 0 | 0 |
| **Monocytes NC I** | 0 | 0 | 0 | 0 | 0.022974 | 0 | 0 | 0 | 0 | 0 | 0 | 0 |
| **Neutrophils** | 3.24E-05 | 0 | 0.120372 | 0.064578 | 0.023646 | 0.055268 | 0.094472 | 0 | 0.048323 | 0.026415 | 0 | 0 |
| **NK** | 0 | 0.021467 | 0 | 0.04216 | 0 | 0.073956 | 0.086575 | 0.008709 | 0.013829 | 0 | 0 | 0.045362 |
| **pDCs** | 0.001481 | 0 | 0.018046 | 0 | 0.041955 | 0 | 0 | 0 | 0.026325 | 0 | 0.062628 | 0.1752 |
| **Plasma** | 0 | 0 | 0.010882 | 0.034706 | 0.029757 | 0 | 0 | 0 | 0 | 0 | 0 | 0 |
| **T CD4 memory** | 0 | 0 | 0 | 0 | 0 | 0 | 0 | 0 | 0 | 0.15054 | 0 | 0 |
| **T CD4 naive** | 0.027262 | 0.098169 | 0 | 0 | 0.404843 | 0 | 0.191012 | 0 | 0 | 0 | 0 | 0 |
| **T CD8 memory** | 0 | 0.100342 | 0.093011 | 0.062206 | 0.277299 | 0.065808 | 0 | 0 | 0 | 0.121347 | 0 | 0.208705 |
| **T CD8 naive** | 0.143755 | 0.123139 | 0 | 0.084861 | 0.007467 | 0.079808 | 0.144721 | 0 | 0 | 0 | 0.054435 | 0 |
| **Treg** | 0 | 0.015803 | 0.142922 | 0.123975 | 0.093774 | 0.268454 | 0.048853 | 0.416676 | 0.051326 | 0 | 0 | 0.057957 |

**Table. S4.**

Patient IDs from TCGA, CPTAC, and E-MTAB-6134 cohorts with related clinical information, glycolytic group assignments, and hypoxia score calculation used for analysis

| **Database** | **ID** | **Site** | **Stage** | **KRAS_Mutation** | **Follow_up_days** | **Vital_Status** | **Glycolytic_Group** | **Hypoxia_Score** |
| --- | --- | --- | --- | --- | --- | --- | --- | --- |
| TCGA | TCGA-2J-AAB1 | Head | Stage IIB | Yes | 66 | 1 | Medium | 2 |
| TCGA | TCGA-2J-AAB4 | Other | Stage IIB | Yes | 729 | 0 | High | 3 |
| TCGA | TCGA-2J-AAB6 | Body | Stage IIA | Yes | 293 | 1 | High | 3 |
| TCGA | TCGA-2J-AAB8 | Head | Stage IIB | Yes | 80 | 0 | Low | 2 |
| TCGA | TCGA-2J-AAB9 | Head | Stage IIB | No | 627 | 1 | Low | 1 |
| TCGA | TCGA-2J-AABA | Head | Stage IIB | No | 607 | 1 | Low | 1 |
| TCGA | TCGA-2J-AABE | Body | Stage IIA | Yes | 676 | 0 | Medium | 3 |
| TCGA | TCGA-2J-AABF | Head | Stage IIB | Yes | 691 | 1 | Low | 1 |
| TCGA | TCGA-2J-AABH | Body | Stage IIA | Yes | 1287 | 0 | Medium | 2 |
| TCGA | TCGA-2J-AABI | Head | Stage IIA | Yes | 969 | 0 | High | 2 |
| TCGA | TCGA-2J-AABK | Other | Stage IIB | Yes | 484 | 0 | Medium | 1 |
| TCGA | TCGA-2J-AABO | Head | Stage IIB | Yes | 440 | 0 | Medium | 2 |
| TCGA | TCGA-2J-AABR | Other | Stage IIA | No | 438 | 0 | Low | 3 |
| TCGA | TCGA-2J-AABT | Head | Stage IIB | Yes | 319 | 0 | Medium | 1 |
| TCGA | TCGA-2J-AABV | Head | Stage IIB | No | 652 | 1 | Low | 1 |
| TCGA | TCGA-2L-AAQA | Head | Stage IIB | Yes | 143 | 1 | High | 3 |
| TCGA | TCGA-2L-AAQE | Head | Stage IIB | Yes | 684 | 1 | High | 1 |
| TCGA | TCGA-2L-AAQI | Head | Stage IIB | Yes | 103 | 1 | High | 2 |
| TCGA | TCGA-2L-AAQJ | Head | Stage III | Yes | 394 | 1 | Medium | 2 |
| TCGA | TCGA-2L-AAQL | Head | Stage IIB | Yes | 292 | 1 | Low | 1 |
| TCGA | TCGA-3A-A9I5 | Other |  | No | 1794 | 0 | Medium | 1 |
| TCGA | TCGA-3A-A9I7 | Head | Stage IIB | Yes | 1323 | 0 | Low | 2 |
| TCGA | TCGA-3A-A9IB | Head | Stage IIB | Yes | 224 | 1 | High | 3 |
| TCGA | TCGA-3A-A9IC | Head | Stage IIB | Yes | 738 | 1 | Medium | 3 |
| TCGA | TCGA-3A-A9IH | Body | Stage IA | Yes | 1021 | 0 | High | 3 |
| TCGA | TCGA-3A-A9IU | Head | Stage IIB | Yes | 458 | 1 | High | 3 |
| TCGA | TCGA-3A-A9IX | Head | Stage IA | No | 1037 | 0 | Low | 2 |
| TCGA | TCGA-3A-A9IZ | Head | Stage IIB | Yes | 308 | 1 | High | 3 |
| TCGA | TCGA-3A-A9J0 | Head | Stage IIB | No | 743 | 0 | High | 3 |
| TCGA | TCGA-3E-AAAY | Other | Stage IIB | No | 2285 | 0 | Low | 2 |
| TCGA | TCGA-3E-AAAZ | Head | Stage IIA | Yes | 2182 | 1 | Low | 1 |
| TCGA | TCGA-F2-6879 | Head | Stage IIB | Yes | 334 | 1 | Medium | 2 |
| TCGA | TCGA-F2-A44G | Head | Stage IIB | Yes | 233 | 1 | High | 3 |
| TCGA | TCGA-F2-A44H | Head | Stage IIA | No | 586 | 0 | Low | 2 |
| TCGA | TCGA-F2-A7TX | Head | Stage IIB | Yes | 95 | 1 | Medium | 1 |
| TCGA | TCGA-F2-A8YN | Head | Stage IIA | Yes | 517 | 0 | Medium | 1 |
| TCGA | TCGA-FB-A4P6 | Head | Stage IIB | No | 767 | 0 | Low | 1 |
| TCGA | TCGA-FB-A5VM | Head | Stage IB | Yes | 498 | 1 | High | 2 |
| TCGA | TCGA-FB-A78T | Head | Stage IIB | No | 375 | 1 | Medium | 1 |
| TCGA | TCGA-FB-AAPQ | Head | Stage IIB | Yes | 1130 | 1 | High | 3 |
| TCGA | TCGA-FB-AAPS | Head | Stage IIB | No | 228 | 0 | Low | 3 |
| TCGA | TCGA-FB-AAPU | Head | Stage IIA | Yes | 381 | 1 | High | 1 |
| TCGA | TCGA-FB-AAPY | Head | Stage IIB | No | 1059 | 1 | Medium | 2 |
| TCGA | TCGA-FB-AAPZ | Head | Stage IIB | Yes | 716 | 0 | Medium | 3 |
| TCGA | TCGA-FB-AAQ0 | Head | Stage IIA | Yes | 473 | 1 | High | 2 |
| TCGA | TCGA-FB-AAQ1 | Head | Stage IIB | Yes | 123 | 1 | High | 2 |
| TCGA | TCGA-FB-AAQ2 | Head | Stage IIB | Yes | 153 | 1 | High | 2 |
| TCGA | TCGA-FB-AAQ3 | Head | Stage IIB | Yes | 31 | 1 | Medium | 1 |
| TCGA | TCGA-FB-AAQ6 | Tail | Stage IA | Yes | 244 | 1 | High | 1 |
| TCGA | TCGA-HV-A5A4 | Body | Stage IIA | No | 232 | 0 | Medium | 3 |
| TCGA | TCGA-HV-A5A5 | Head | Stage IIB | No | 289 | 0 | Low | 1 |
| TCGA | TCGA-HV-A5A6 | Head | Stage IIB | Yes | 2036 | 1 | Medium | 1 |
| TCGA | TCGA-HV-A7OL | Head | Stage IIA | Yes | 252 | 0 | High | 3 |
| TCGA | TCGA-HV-AA8V | Head | Stage IIB | Yes | 920 | 0 | Medium | 2 |
| TCGA | TCGA-HV-AA8X | Body | Stage IIB | Yes | 532 | 1 | High | 1 |
| TCGA | TCGA-HZ-7919 | Head | Stage IIB | No | 593 | 1 | High | 3 |
| TCGA | TCGA-HZ-7922 | Head | Stage IIB | Yes | 0 | 0 | High | 3 |
| TCGA | TCGA-HZ-7925 | Head | Stage IIB | Yes | 614 | 1 | Medium | 3 |
| TCGA | TCGA-HZ-7926 | Head | Stage IIB | Yes | 518 | 1 | Medium | 3 |
| TCGA | TCGA-HZ-8001 | Head | Stage III | No | 706 | 0 | Medium | 1 |
| TCGA | TCGA-HZ-8002 | Head | Stage IIB | No | 366 | 1 | Low | 1 |
| TCGA | TCGA-HZ-8003 | Head | Stage IIB | No | 596 | 1 | Low | 1 |
| TCGA | TCGA-HZ-8005 | Other | Stage IIB | Yes | 120 | 1 | High | 3 |
| TCGA | TCGA-HZ-8315 | Head | Stage IIA | Yes | 299 | 1 | Low | 2 |
| TCGA | TCGA-HZ-8317 | Head | Stage IIB | No | 378 | 1 | Low | 2 |
| TCGA | TCGA-HZ-8636 | Tail | Stage IV | Yes | 545 | 1 | Medium | 3 |
| TCGA | TCGA-HZ-8637 | Head | Stage IIB | No | 517 | 1 | Low | 1 |
| TCGA | TCGA-HZ-A49H | Head | Stage IIB | Yes | 491 | 0 | Low | 1 |
| TCGA | TCGA-HZ-A49I | Head | Stage IIB | Yes | 308 | 1 | Medium | 3 |
| TCGA | TCGA-HZ-A4BH | Head | Stage IIB | Yes | 194 | 0 | Medium | 3 |
| TCGA | TCGA-HZ-A4BK | Head | Stage IIB | Yes | 657 | 0 | Low | 2 |
| TCGA | TCGA-HZ-A77O | Head | Stage IIB | Yes | 160 | 1 | High | 2 |
| TCGA | TCGA-HZ-A77P | Head | Stage IIB | No | 330 | 0 | Low | 1 |
| TCGA | TCGA-HZ-A77Q | Head | Stage IIB | Yes | 33 | 0 | Medium | 3 |
| TCGA | TCGA-HZ-A8P0 | Head | Stage IIB | Yes | 0 | 0 | Medium | 1 |
| TCGA | TCGA-HZ-A8P1 | Tail | Stage IB | Yes | 0 | 0 | Low | 1 |
| TCGA | TCGA-IB-7644 | Head | Stage IV | Yes | 394 | 1 | Medium | 2 |
| TCGA | TCGA-IB-7645 | Head | Stage IIB | Yes | 1502 | 1 | Low | 3 |
| TCGA | TCGA-IB-7646 | Head | Stage IIB | Yes | 145 | 1 | High | 3 |
| TCGA | TCGA-IB-7647 | Head | Stage IIB | No | 666 | 1 | High | 3 |
| TCGA | TCGA-IB-7649 | Head | Stage IIB | Yes | 467 | 1 | Low | 2 |
| TCGA | TCGA-IB-7651 | Head | Stage IIB | No | 603 | 1 | High | 2 |
| TCGA | TCGA-IB-7652 | Body | Stage IIB | No | 1116 | 0 | Medium | 2 |
| TCGA | TCGA-IB-7885 | Head | Stage IIB | No | 1257 | 0 | High | 3 |
| TCGA | TCGA-IB-7886 | Head | Stage IIB | Yes | 123 | 1 | Medium | 3 |
| TCGA | TCGA-IB-7887 | Head | Stage IIB | Yes | 110 | 1 | Medium | 3 |
| TCGA | TCGA-IB-7888 | Head | Stage IIA | No | 1332 | 1 | Low | 2 |
| TCGA | TCGA-IB-7889 | Head | Stage IIB | Yes | 481 | 1 | Low | 2 |
| TCGA | TCGA-IB-7890 | Tail | Stage IB | Yes | 598 | 1 | High | 3 |
| TCGA | TCGA-IB-7891 | Head | Stage IIB | No | 913 | 1 | Low | 1 |
| TCGA | TCGA-IB-7893 | Body | Stage IIA | Yes | 117 | 1 | High | 3 |
| TCGA | TCGA-IB-7897 | Head | Stage IIB | No | 486 | 1 | Low | 1 |
| TCGA | TCGA-IB-8126 | Head | Stage III | No | 462 | 0 | Low | 1 |
| TCGA | TCGA-IB-8127 | Head | Stage IIB | Yes | 522 | 0 | Medium | 1 |
| TCGA | TCGA-IB-A5SO | Head | Stage IIB | Yes | 365 | 1 | Medium | 3 |
| TCGA | TCGA-IB-A5SP | Head | Stage IIA | Yes | 482 | 0 | High | 1 |
| TCGA | TCGA-IB-A5SQ | Head | Stage IB | No | 219 | 1 | Medium | 3 |
| TCGA | TCGA-IB-A5SS | Tail | Stage IIB | Yes | 460 | 1 | High | 3 |
| TCGA | TCGA-IB-A5ST | Head | Stage IIB | No | 635 | 0 | Low | 2 |
| TCGA | TCGA-IB-A6UF | Head | Stage IIB | Yes | 666 | 0 | High | 2 |
| TCGA | TCGA-IB-A6UG | Head | Stage IIB | Yes | 41 | 1 | Low | 1 |
| TCGA | TCGA-IB-A7LX | Head | Stage IIB | Yes | 250 | 1 | High | 2 |
| TCGA | TCGA-IB-A7M4 | Body | Stage IIB | Yes | 483 | 0 | High | 2 |
| TCGA | TCGA-IB-AAUM | Head | Stage IIB | Yes | 0 | 0 | Low | 1 |
| TCGA | TCGA-IB-AAUN | Head | Stage IB | Yes | 144 | 1 | Medium | 2 |
| TCGA | TCGA-IB-AAUO | Head | Stage IIB | Yes | 239 | 1 | High | 3 |
| TCGA | TCGA-IB-AAUP | Head | Stage IIB | No | 431 | 0 | Low | 2 |
| TCGA | TCGA-IB-AAUQ | Tail | Stage IIB | Yes | 183 | 1 | High | 3 |
| TCGA | TCGA-IB-AAUR | Head | Stage IIB | No | 338 | 0 | Low | 2 |
| TCGA | TCGA-IB-AAUS | Other | Stage IIB | No | 225 | 0 | Medium | 3 |
| TCGA | TCGA-IB-AAUT | Head | Stage IIB | No | 287 | 0 | Low | 2 |
| TCGA | TCGA-IB-AAUU | Head | Stage IIB | Yes | 245 | 0 | High | 3 |
| TCGA | TCGA-LB-A7SX | Head | Stage IIB | No | 393 | 1 | Low | 1 |
| TCGA | TCGA-LB-A8F3 | Head | Stage IIA | No | 379 | 0 | Medium | 1 |
| TCGA | TCGA-LB-A9Q5 | Head | Stage IIB | No | 313 | 1 | Medium | 2 |
| TCGA | TCGA-M8-A5N4 | Head | Stage IIA | No | 584 | 0 | High | 3 |
| TCGA | TCGA-OE-A75W | Tail | Stage IIA | No | 267 | 1 | High | 2 |
| TCGA | TCGA-PZ-A5RE | Head | Stage IIB | No | 470 | 1 | High | 3 |
| TCGA | TCGA-Q3-A5QY | Head | Stage IIB | No | 416 | 0 | Low | 2 |
| TCGA | TCGA-Q3-AA2A | Head | Stage IB | Yes | 95 | 0 | Medium | 2 |
| TCGA | TCGA-RB-A7B8 | Head | Stage IIB | Yes | 466 | 1 | Medium | 2 |
| TCGA | TCGA-RB-AA9M | Tail | Stage IIB | No | 286 | 0 | Low | 1 |
| TCGA | TCGA-S4-A8RM | Other | Stage IIB | Yes | 737 | 0 | Low | 1 |
| TCGA | TCGA-S4-A8RO | Head | Stage IIB | Yes | 525 | 0 | High | 2 |
| TCGA | TCGA-S4-A8RP | Head | Stage IIB | Yes | 702 | 1 | Low | 1 |
| TCGA | TCGA-US-A774 | Head | Stage IIB | No | 695 | 1 | Low | 3 |
| TCGA | TCGA-US-A776 | Head | Stage IIA | No | 1216 | 0 | Medium | 1 |
| TCGA | TCGA-US-A779 | Head | Stage IIB | Yes | 511 | 1 | Medium | 1 |
| TCGA | TCGA-US-A77E | Head | Stage IIB | No | 430 | 1 | Medium | 2 |
| TCGA | TCGA-US-A77G | Head | Stage IIB | Yes | 0 | 0 | Medium | 1 |
| TCGA | TCGA-XD-AAUG | Other | Stage IV | No | 420 | 0 | Low | 2 |
| TCGA | TCGA-XD-AAUH | Head | Stage IIB | No | 395 | 0 | Low | 1 |
| TCGA | TCGA-XD-AAUI | Head | Stage IIB | Yes | 366 | 1 | High | 3 |
| TCGA | TCGA-XD-AAUL | Head | Stage IIA | Yes | 498 | 0 | Medium | 2 |
| TCGA | TCGA-XN-A8T3 | Head | Stage IB | Yes | 951 | 0 | Medium | 1 |
| TCGA | TCGA-XN-A8T5 | Head | Stage IB | No | 720 | 0 | Low | 2 |
| TCGA | TCGA-YH-A8SY | Head |  | Yes | 388 | 0 | High | 3 |
| TCGA | TCGA-YY-A8LH | Tail | Stage IIB | Yes | 2016 | 0 | High | 1 |
| TCGA | TCGA-Z5-AAPL | Other | Stage IIA | No | 467 | 0 | Low | 1 |

| **Database** | **ID** | **Site** | **Stage** | **KRAS_Mutation** | **Follow_up_days** | **Vital_Status** | **Glycolytic_Group** | **Hypoxia_Score** |
| --- | --- | --- | --- | --- | --- | --- | --- | --- |
| CPTAC | C3L.00017.02 | Head | IIA | Yes | 426 | 0 | Low | 1 |
| CPTAC | C3L.00102.01 | Head | III | Yes | 249 | 1 | High | 3 |
| CPTAC | C3L.00277.03 | Tail | IIB | Yes | 610 | 1 | High | 2 |
| CPTAC | C3L.00589.01.C3L.00589.02 | Head | IIB | Yes | 1046 | 0 | Low | 1 |
| CPTAC | C3L.00598.02.C3L.00598.03 | Head | III | Yes | 422 | 1 | High | 2 |
| CPTAC | C3L.00599.01.C3L.00599.03.C3L.00599.02 | Head | IIB | Yes | 1189 | 0 | Low | 1 |
| CPTAC | C3L.00622.01.C3L.00622.03 | Head | IIB | Yes | 311 | 1 | Medium | 2 |
| CPTAC | C3L.00625.03.C3L.00625.02 | Head | III | Yes | 864 | 0 | High | 3 |
| CPTAC | C3L.00819.01 | Head | IIB | Yes | 602 | 1 | High | 3 |
| CPTAC | C3L.00928.03 | Tail | IIB | Yes | 761 | 1 | High | 3 |
| CPTAC | C3L.01031.01 | Other | IB | Yes | 369 | 0 | Medium | 1 |
| CPTAC | C3L.01037.01 | Head | IIB | Yes | 300 | 1 | High | 3 |
| CPTAC | C3L.01051.02 | Head | IIB | Yes | 0 | 0 | Low | 1 |
| CPTAC | C3L.01124.01 | Head | III | Yes | 399 | 0 | Low | 1 |
| CPTAC | C3L.01328.01 | Head | IIB | Yes | 270 | 0 | High | 3 |
| CPTAC | C3L.01453.03 | Tail | IIA | Yes | 5 | 0 | Medium | 2 |
| CPTAC | C3L.01598.02 | Tail | IB | Yes | 912 | 1 | High | 3 |
| CPTAC | C3L.01637.02.C3L.01637.03 | Body | IIB | Yes | 715 | 1 | High | 3 |
| CPTAC | C3L.01662.03 | Head | III | Yes | 344 | 1 | Medium | 1 |
| CPTAC | C3L.01687.03.C3L.01687.02.C3L.01687.01 | Head | III | Yes | 376 | 1 | Low | 2 |
| CPTAC | C3L.01689.03.C3L.01689.02 | Head | IIB | Yes | 594 | 1 | High | 3 |
| CPTAC | C3L.01703.02.C3L.01703.03 | Other | IIA | Yes | 287 | 1 | Low | 1 |
| CPTAC | C3L.01971.02 | Head | IA | Yes | 276 | 1 | Low | 1 |
| CPTAC | C3L.02109.02.C3L.02109.03 | Body | IIB | Yes | 261 | 0 | High | 3 |
| CPTAC | C3L.02115.02.C3L.02115.03 | Head | III | Yes | 723 | 1 | Low | 1 |
| CPTAC | C3L.02116.03.C3L.02116.01 | Body | IB | Yes | 716 | 0 | Low | 2 |
| CPTAC | C3L.02118.01.C3L.02118.02 | Head | IB | Yes | 632 | 1 | Medium | 2 |
| CPTAC | C3L.02463.02.C3L.02463.01 | Head | IB | Yes | 740 | 0 | High | 2 |
| CPTAC | C3L.02604.01 | Tail | IIB | Yes | 118 | 1 | High | 3 |
| CPTAC | C3L.02606.02.C3L.02606.01 | Head | IIB | Yes | 403 | 1 | High | 3 |
| CPTAC | C3L.02610.03.C3L.02610.01 | Head | IIA | Yes | 190 | 1 | Medium | 1 |
| CPTAC | C3L.02613.02.C3L.02613.03 | Head | IIB | Yes | 211 | 1 | High | 3 |
| CPTAC | C3L.02701.02 | Other | NotAva | Yes | 243 | 1 | Medium | 3 |
| CPTAC | C3L.02809.04 | Head | IIB | Yes | 68 | 1 | Medium | 3 |
| CPTAC | C3L.02890.02.C3L.02890.01 | Head | III | Yes | 785 | 0 | Low | 1 |
| CPTAC | C3L.02897.01 | Head | IB | Yes | 256 | 0 | Medium | 2 |
| CPTAC | C3L.02899.01 | Head | IV | Yes | 90 | 1 | High | 3 |
| CPTAC | C3L.03123.02 | Head | IIB | Yes | 817 | 0 | High | 3 |
| CPTAC | C3L.03356.02 | Head | IIB | Yes | 549 | 0 | Low | 1 |
| CPTAC | C3L.03388.02 | Head | III | Yes | 11 | 1 | Low | 2 |
| CPTAC | C3L.03628.02.C3L.03628.01 | Head | III | Yes | 466 | 0 | Medium | 3 |
| CPTAC | C3L.03630.03.C3L.03630.02 | Head | IIB | Yes | 78 | 0 | Medium | 1 |
| CPTAC | C3L.03635.01.C3L.03635.02 | Tail | NotAva | Yes | 313 | 0 | Medium | 2 |
| CPTAC | C3L.03639.03.C3L.03639.02.C3L.03639.01 | Head | IB | Yes | 246 | 1 | High | 3 |
| CPTAC | C3L.03743.01 | Head | IIB | Yes | 0 | 1 | High | 2 |
| CPTAC | C3L.04027.01 | Head | IA | Yes | 240 | 0 | Medium | 3 |
| CPTAC | C3L.04072.01 | Head | III | Yes | 225 | 1 | High | 1 |
| CPTAC | C3L.04080.02.C3L.04080.03 | Head | IIB | Yes | 20 | 1 | Medium | 2 |
| CPTAC | C3L.04475.01.C3L.04475.02 | Head | III | Yes | 300 | 0 | Medium | 2 |
| CPTAC | C3L.04479.02.C3L.04479.01 | Head | IIB | Yes | 356 | 0 | Low | 1 |
| CPTAC | C3L.04848.03.C3L.04848.01 | Head | IIB | Yes | 196 | 1 | Low | 2 |
| CPTAC | C3L.04853.01.C3L.04853.02 | Head | IB | Yes | 279 | 1 | Medium | 2 |
| CPTAC | C3N.00198.02 | Body | III | No | 1364 | 0 | Low | 2 |
| CPTAC | C3N.00249.03 | Body | IIB | Yes | 1098 | 0 | Low | 1 |
| CPTAC | C3N.00302.02 | Head | IV | Yes | 323 | 1 | High | 3 |
| CPTAC | C3N.00436.01 | Head | IIA | Yes | 652 | 0 | Medium | 2 |
| CPTAC | C3N.00511.03 | Head | IIB | Yes | 1001 | 1 | Medium | 1 |
| CPTAC | C3N.00514.01.C3N.00514.02 | Head | III | Yes | 795 | 1 | Low | 1 |
| CPTAC | C3N.00516.02.C3N.00516.01 | Head | IB | Yes | 688 | 0 | High | 3 |
| CPTAC | C3N.00518.01 | Head | IIA | Yes | 469 | 1 | Low | 2 |
| CPTAC | C3N.00709.01 | Other | NotAva | Yes | 0 | 0 | Low | 1 |
| CPTAC | C3N.01012.02.C3N.01012.01 | Head | IB | No | 616 | 0 | Low | 1 |
| CPTAC | C3N.01165.03.C3N.01165.02 | Head | III | Yes | 240 | 1 | Medium | 2 |
| CPTAC | C3N.01167.01.C3N.01167.02 | Head | IIB | Yes | 640 | 0 | Low | 1 |
| CPTAC | C3N.01168.02 | Head | IIB | Yes | 460 | 1 | Low | 1 |
| CPTAC | C3N.01375.02.C3N.01375.03 | Head | IIB | Yes | 142 | 1 | Medium | 3 |
| CPTAC | C3N.01380.01.C3N.01380.03 | Head | IV | No | 400 | 1 | Low | 1 |
| CPTAC | C3N.01381.02.C3N.01381.01 | Head | IIB | Yes | 454 | 1 | Low | 1 |
| CPTAC | C3N.01383.03.C3N.01383.01 | Head | III | Yes | 667 | 0 | Medium | 3 |
| CPTAC | C3N.01388.03 | Head | III | Yes | 810 | 1 | Medium | 3 |
| CPTAC | C3N.01714.02 | Head | III | Yes | 435 | 1 | High | 3 |
| CPTAC | C3N.01716.03 | Head | IV | Yes | 335 | 1 | High | 2 |
| CPTAC | C3N.02010.03 | Head | III | Yes | 359 | 0 | Low | 1 |
| CPTAC | C3N.02573.01.C3N.02573.02 | Other | IIB | Yes | 479 | 0 | Medium | 2 |
| CPTAC | C3N.02579.02 | Head | IA | Yes | 358 | 1 | Medium | 2 |
| CPTAC | C3N.02585.01.C3N.02585.02 | Body | IA | Yes | 754 | 0 | Medium | 2 |
| CPTAC | C3N.02589.01 | Head | IA | Yes | 458 | 1 | Low | 1 |
| CPTAC | C3N.02592.01 | Head | IIB | Yes | 294 | 1 | Low | 1 |
| CPTAC | C3N.02768.02.C3N.02768.01 | Tail | IIB | Yes | 430 | 1 | High | 2 |
| CPTAC | C3N.02940.01.C3N.02940.02 | Head | III | Yes | 664 | 0 | Low | 1 |
| CPTAC | C3N.02944.01 | Tail | IB | Yes | 609 | 0 | Medium | 2 |
| CPTAC | C3N.02971.01 | Tail | IIA | Yes | 70 | 1 | Low | 2 |
| CPTAC | C3N.03000.02 | Head | IV | Yes | 122 | 1 | High | 3 |
| CPTAC | C3N.03006.02 | Head | IIB | Yes | 708 | 0 | Low | 1 |
| CPTAC | C3N.03007.02 | Head | III | Yes | 58 | 1 | High | 3 |
| CPTAC | C3N.03039.01 | Other | NotAva | Yes | 0 | 0 | High | 2 |
| CPTAC | C3N.03061.01.C3N.03061.02 | Body | IIB | Yes | 348 | 1 | Medium | 2 |
| CPTAC | C3N.03086.02 | Body | IIB | Yes | 733 | 0 | Low | 1 |
| CPTAC | C3N.03173.01 | Head | III | Yes | 32 | 0 | Low | 1 |
| CPTAC | C3N.03190.01.C3N.03190.02 | Body | IIB | Yes | 398 | 1 | Medium | 2 |
| CPTAC | C3N.03426.01 | Head | IV | No | 122 | 1 | High | 3 |
| CPTAC | C3N.03430.02 | Head | IV | Yes | 39 | 1 | High | 2 |
| CPTAC | C3N.03439.02 | Head | IIB | Yes | 457 | 1 | Low | 1 |
| CPTAC | C3N.03665.01 | Head | III | Yes | 728 | 0 | Medium | 3 |
| CPTAC | C3N.03666.01 | Head | IIB | Yes | 701 | 1 | Medium | 3 |
| CPTAC | C3N.03754.01 | Head | III | Yes | 483 | 0 | Medium | 2 |
| CPTAC | C3N.03780.01 | Head | IB | Yes | 20 | 1 | Low | 1 |
| CPTAC | C3N.03839.05.C3N.03839.02.C3N.03839.01 | Head | IIB | Yes | 696 | 0 | Medium | 2 |
| CPTAC | C3N.03840.02.C3N.03840.01 | Body | IIB | Yes | 696 | 0 | Medium | 1 |
| CPTAC | C3N.03853.02.C3N.03853.01 | Head | III | Yes | 259 | 0 | Medium | 3 |
| CPTAC | C3N.03884.01 | Head | III | Yes | 368 | 0 | High | 3 |
| CPTAC | C3N.04119.02 | Tail | IIB | Yes | 305 | 1 | High | 2 |
| CPTAC | C3N.04126.02.C3N.04126.01 | Head | III | Yes | 348 | 1 | Medium | 2 |
| CPTAC | C3N.04282.01 | Head | IV | Yes | 303 | 1 | High | 3 |
| CPTAC | C3N.04283.03 | Other | III | Yes | 1 | 1 | High | 3 |

| **Database** | **ID** | **Stage** | **KRAS_Mutation** | **Follow_up_mothss** | **Vital_Status** | **Glycolytic_Group** | **Hypoxia_Score** |
| --- | --- | --- | --- | --- | --- | --- | --- |
| E-MTAB-6134 | A11_C05.CEL | III | Yes | 49.9 | 1 | Medium | 2 |
| E-MTAB-6134 | A12_D05.CEL | IIB | Yes | 54.05 | 1 | Low | 2 |
| E-MTAB-6134 | A13_F05.CEL | III | Yes | 24.05 | 1 | Medium | 2 |
| E-MTAB-6134 | A14_C07.CEL | III | Yes | 41.88 | 1 | Medium | 2 |
| E-MTAB-6134 | A15_A07.CEL | III | Yes | 56.94 | 0 | Medium | 1 |
| E-MTAB-6134 | A16_H05.CEL | IIB | Yes | 37.01 | 0 | Low | 1 |
| E-MTAB-6134 | A2_E07.CEL | IIA | Yes | 18.42 | 1 | High | 2 |
| E-MTAB-6134 | A2299_001.CEL | IIB | Yes | 10.23 | 1 | Low | 1 |
| E-MTAB-6134 | A2299_004.CEL | III | Yes | 23.13 | 1 | Low | 1 |
| E-MTAB-6134 | A2299_006.CEL | III | No | 45.63 | 0 | Medium | 1 |
| E-MTAB-6134 | A2299_007.CEL | III | Yes | 28.03 | 0 | Medium | 1 |
| E-MTAB-6134 | A2299_008.CEL | IIA | Yes | 9.01 | 1 | Low | 1 |
| E-MTAB-6134 | A2299_009.CEL | III | Yes | 11.05 | 0 | Low | 1 |
| E-MTAB-6134 | A2299_010.CEL | III | No | 32.83 | 0 | Low | 1 |
| E-MTAB-6134 | A2299_011.CEL | III | Yes | 10.77 | 0 | Medium | 1 |
| E-MTAB-6134 | A2299_012_rr.CEL | III | Yes | 24.08 | 1 | High | 1 |
| E-MTAB-6134 | A2299_013.CEL | III | Yes | 24.38 | 1 | Medium | 2 |
| E-MTAB-6134 | A2299_014.CEL | III | Yes | 12.2 | 1 | Medium | 1 |
| E-MTAB-6134 | A2299_015.CEL | III | Yes | 35.33 | 1 | Medium | 1 |
| E-MTAB-6134 | A2299_018.CEL | III | Yes | 94.13 | 0 | Low | 1 |
| E-MTAB-6134 | A2299_019.CEL | IIA | Yes | 130.2 | 1 | Low | 1 |
| E-MTAB-6134 | A2299_020.CEL | III | Yes | 12.1 | 1 | Medium | 2 |
| E-MTAB-6134 | A2299_021.CEL | III | Yes | 34.3 | 0 | Low | 1 |
| E-MTAB-6134 | A2299_022.CEL | III | Yes | 19.34 | 0 | Low | 1 |
| E-MTAB-6134 | A2299_023.CEL | IA | Yes | 26.91 | 1 | Low | 1 |
| E-MTAB-6134 | A2299_024.CEL | III | Yes | 13.82 | 1 | Medium | 1 |
| E-MTAB-6134 | A2299_027.CEL | IIA | Yes | 26.84 | 1 | Low | 1 |
| E-MTAB-6134 | A2299_031.CEL | III | No | 1.12 | 1 | Medium | 2 |
| E-MTAB-6134 | A2299_032.CEL | III | Yes | 15.03 | 1 | Low | 1 |
| E-MTAB-6134 | A2299_033.CEL | IIA | Yes | 1.35 | 1 | High | 2 |
| E-MTAB-6134 | A2299_034.CEL | IIA | Yes | 1.18 | 1 | Low | 1 |
| E-MTAB-6134 | A2299_035.CEL | IA | Yes | 21.09 | 1 | Low | 1 |
| E-MTAB-6134 | A2299_036.CEL | III | Yes | 9.8 | 1 | Low | 1 |
| E-MTAB-6134 | A2299_037.CEL | III | Yes | 11.15 | 1 | High | 3 |
| E-MTAB-6134 | A2299_038.CEL | IIB | Yes | 8.65 | 1 | Low | 1 |
| E-MTAB-6134 | A2299_039.CEL | III | Yes | 12.93 | 0 | Medium | 2 |
| E-MTAB-6134 | A2299_040.CEL | III | Yes | 21.94 | 1 | Low | 1 |
| E-MTAB-6134 | A2299_041.CEL | IIA | Yes | 30.39 | 0 | Low | 1 |
| E-MTAB-6134 | A2299_042.CEL | IIA | No | 39.74 | 0 | Medium | 2 |
| E-MTAB-6134 | A2299_043.CEL | III | No | 6.84 | 1 | Low | 1 |
| E-MTAB-6134 | A2299_044.CEL | III | Yes | 7.96 | 0 | Medium | 1 |
| E-MTAB-6134 | A2299_045.CEL | IIA | Yes | 59.33 | 0 | Low | 1 |
| E-MTAB-6134 | A2299_046.CEL | IIA | Yes | 54.73 | 0 | Low | 1 |
| E-MTAB-6134 | A2299_047.CEL | IIA | Yes | 10.16 | 1 | Medium | 2 |
| E-MTAB-6134 | A2299_048.CEL | III | No | 7.57 | 1 | Low | 1 |
| E-MTAB-6134 | A2299_050.CEL | III | Yes | 37.4 | 1 | Low | 1 |
| E-MTAB-6134 | A2299_051.CEL | IIA | Yes | 44.31 | 1 | High | 2 |
| E-MTAB-6134 | A2299_052.CEL | III | Yes | 12.03 | 1 | High | 3 |
| E-MTAB-6134 | A2299_053.CEL | III | No | 9.21 | 1 | Low | 1 |
| E-MTAB-6134 | A2299_054.CEL | III | No | 19.24 | 1 | Low | 1 |
| E-MTAB-6134 | A2299_055.CEL | IIB | Yes | 42.53 | 1 | Medium | 2 |
| E-MTAB-6134 | A2299_056.CEL | III | Yes | 16.48 | 1 | High | 1 |
| E-MTAB-6134 | A2299_057.CEL | III | Yes | 10.43 | 1 | Medium | 1 |
| E-MTAB-6134 | A2299_058.CEL | III | Yes | 14.64 | 1 | Medium | 1 |
| E-MTAB-6134 | A2299_059.CEL | III | Yes | 12.96 | 1 | Low | 1 |
| E-MTAB-6134 | A2299_060.CEL | III | Yes | 14.67 | 0 | Low | 1 |
| E-MTAB-6134 | A2299_061.CEL | III | Yes | 42.6 | 1 | Medium | 2 |
| E-MTAB-6134 | A2299_062.CEL | III | Yes | 10 | 1 | High | 2 |
| E-MTAB-6134 | A2299_063.CEL | IIA | Yes | 21.91 | 0 | High | 2 |
| E-MTAB-6134 | A2299_064.CEL | III | No | 12.11 | 1 | Medium | 1 |
| E-MTAB-6134 | A2299_065.CEL | III | Yes | 16.38 | 1 | High | 2 |
| E-MTAB-6134 | A2299_066.CEL | III | Yes | 16.94 | 1 | Low | 1 |
| E-MTAB-6134 | A2299_067.CEL | III | Yes | 20.2 | 1 | High | 3 |
| E-MTAB-6134 | A2299_068.CEL | IIB | No | 40.83 | 0 | Low | 1 |
| E-MTAB-6134 | A2299_069.CEL | III | Yes | 3.88 | 1 | High | 2 |
| E-MTAB-6134 | A2299_070.CEL | III | Yes | 17.99 | 1 | Low | 1 |
| E-MTAB-6134 | A2299_072.CEL | III | Yes | 9.47 | 1 | High | 2 |
| E-MTAB-6134 | A2299_073.CEL | III | Yes | 19.8 | 1 | Medium | 1 |
| E-MTAB-6134 | A2299_074.CEL | III | Yes | 19.54 | 1 | Low | 1 |
| E-MTAB-6134 | A2299_075.CEL | III | Yes | 13.82 | 1 | Medium | 3 |
| E-MTAB-6134 | A2299_077.CEL | III | Yes | 40.36 | 1 | Low | 1 |
| E-MTAB-6134 | A2299_078.CEL | III | Yes | 11.78 | 1 | Medium | 1 |
| E-MTAB-6134 | A2299_079.CEL | IIA | Yes | 12.86 | 0 | Medium | 2 |
| E-MTAB-6134 | A2299_081.CEL | III | Yes | 20.53 | 1 | Low | 1 |
| E-MTAB-6134 | A3_G07.CEL | IIA | Yes | 67.07 | 1 | Low | 1 |
| E-MTAB-6134 | A5_F07.CEL | III | Yes | 20.46 | 1 | Low | 1 |
| E-MTAB-6134 | A6_B05.CEL | III | Yes | 38.03 | 1 | Low | 2 |
| E-MTAB-6134 | A7_G05.CEL | III | Yes | 12.4 | 0 | Low | 1 |
| E-MTAB-6134 | A8_A05.CEL | III | Yes | 61.81 | 0 | Medium | 2 |
| E-MTAB-6134 | A9_H07.CEL | III | Yes | 23.95 | 1 | High | 2 |
| E-MTAB-6134 | B00127107.B4_H09.CEL | III | Yes | 12.89 | 1 | High | 3 |
| E-MTAB-6134 | B00133492.D_A10.CEL | IIB | Yes | 6.09 | 1 | High | 3 |
| E-MTAB-6134 | B00142230.C2_A10.CEL | IIA | Yes | 28.68 | 1 | Medium | 3 |
| E-MTAB-6134 | B00143363.H_H06.CEL | III | Yes | 32.99 | 1 | Low | 1 |
| E-MTAB-6134 | B00147787.B7_A07.CEL | IIA | Yes | 8.91 | 1 | Low | 2 |
| E-MTAB-6134 | B00148007.F7_G09.CEL | IIA | Yes | 13.29 | 1 | High | 3 |
| E-MTAB-6134 | B00155099.B4_A11.CEL | IA | No | 57.89 | 1 | Low | 1 |
| E-MTAB-6134 | B00158539.AR_H07.CEL | IA | Yes | 99.84 | 0 | High | 3 |
| E-MTAB-6134 | B00158661.B8_C12.CEL | IIB | No | 101.05 | 0 | Medium | 2 |
| E-MTAB-6134 | B00160317.B7_G12.CEL | III | Yes | 105.69 | 0 | Medium | 3 |
| E-MTAB-6134 | B00161568.D7_G11.CEL | III | No | 53.45 | 1 | Medium | 3 |
| E-MTAB-6134 | B00166629.C4II_E08.CEL | III | Yes | 18.49 | 1 | High | 3 |
| E-MTAB-6134 | B00169543.B7_D09.CEL | IIB | Yes | 15.82 | 1 | High | 2 |
| E-MTAB-6134 | B00170245.D5_D06.CEL | IIA | Yes | 70.07 | 1 | Medium | 3 |
| E-MTAB-6134 | B00175584.A8_A03.CEL | III | No | 11.88 | 1 | High | 3 |
| E-MTAB-6134 | B00176387.D_A01.CEL | IIB | Yes | 17.66 | 1 | High | 3 |
| E-MTAB-6134 | B00177224.A10_E03.CEL | III | Yes | 21.88 | 1 | Low | 1 |
| E-MTAB-6134 | B00178927.B8_C08.CEL | III | Yes | 5.07 | 1 | High | 3 |
| E-MTAB-6134 | B00180921.J_C09.CEL | III | Yes | 33.32 | 1 | Medium | 1 |
| E-MTAB-6134 | B00182098_E11.CEL | IIA | Yes | 8.06 | 1 | High | 2 |
| E-MTAB-6134 | B00182146.A7_D09.CEL | III | Yes | 20.66 | 1 | High | 3 |
| E-MTAB-6134 | B00182429_D07.CEL | III | Yes | 16.09 | 1 | Medium | 1 |
| E-MTAB-6134 | B00182638.A9_E09.CEL | III | Yes | 8.65 | 1 | Medium | 3 |
| E-MTAB-6134 | B00183300.E_G12.CEL | IA | Yes | 42.34 | 0 | Medium | 2 |
| E-MTAB-6134 | B00183314B10_E01.CEL | IIA | Yes | 10.56 | 1 | High | 3 |
| E-MTAB-6134 | B00185637.K_B09.CEL | III | Yes | 11.02 | 1 | Medium | 2 |
| E-MTAB-6134 | B00188495.B7_A07.CEL | III | Yes | 27.53 | 1 | High | 3 |
| E-MTAB-6134 | B00189185.B8_A06.CEL | III | Yes | 50.63 | 0 | Medium | 2 |
| E-MTAB-6134 | B00190698.B5_A09.CEL | III | Yes | 3.06 | 1 | High | 3 |
| E-MTAB-6134 | B00197952.E13_H09.CEL | IIB | Yes | 30.43 | 1 | Medium | 2 |
| E-MTAB-6134 | B00198520.R3_E07.CEL | IIA | Yes | 38.95 | 0 | Medium | 2 |
| E-MTAB-6134 | B00199393.L_C08.CEL | IIA | No | 25.56 | 0 | High | 2 |
| E-MTAB-6134 | B00202241B15_H09.CEL | IIB | Yes | 20.46 | 0 | High | 1 |
| E-MTAB-6134 | B00202796.B9_G07.CEL | IIA | Yes | 19.77 | 0 | Medium | 2 |
| E-MTAB-6134 | B00203003.61_H05.CEL | III | Yes | 22.76 | 1 | Low | 1 |
| E-MTAB-6134 | B00203410.B9_H02.CEL | IA | Yes | 18.62 | 0 | Medium | 2 |
| E-MTAB-6134 | B00203548.C5_A09.CEL | IIB | Yes | 26.78 | 0 | Medium | 2 |
| E-MTAB-6134 | B00204301.M_G05.CEL | III | Yes | 17.01 | 0 | High | 3 |
| E-MTAB-6134 | B00204637.C82_F01.CEL | IIA | Yes | 24.87 | 0 | Medium | 2 |
| E-MTAB-6134 | B00207167.B75_E12.CEL | IIA | Yes | 11.64 | 0 | High | 2 |
| E-MTAB-6134 | B00208014.B61_C01.CEL | III | Yes | 10.1 | 0 | Low | 1 |
| E-MTAB-6134 | B00208817.C15_B02.CEL | IIB | Yes | 15.82 | 1 | High | 3 |
| E-MTAB-6134 | B00209142.B7_F11.CEL | IIA | Yes | 8.06 | 0 | High | 3 |
| E-MTAB-6134 | B00211803.B6_C09.CEL | III | Yes | 11.74 | 0 | High | 3 |
| E-MTAB-6134 | B00211833.Rep8_F10.CEL | III | Yes | 14.74 | 0 | Low | 1 |
| E-MTAB-6134 | B00212120.C19_B06.CEL | IIA | Yes | 13.95 | 0 | Medium | 2 |
| E-MTAB-6134 | B00212558.C22_C02.CEL | III | Yes | 11.64 | 0 | Low | 1 |
| E-MTAB-6134 | B00212666.C6_D08.CEL | IIB | Yes | 12.34 | 0 | Medium | 2 |
| E-MTAB-6134 | B00212698.D_E06.CEL | IIB | Yes | 12.63 | 0 | High | 3 |
| E-MTAB-6134 | G001248_B01.CEL | III | Yes | 109.38 | 0 | High | 3 |
| E-MTAB-6134 | G001940_B09.CEL | III | Yes | 5.79 | 1 | High | 2 |
| E-MTAB-6134 | G002167_B11.CEL | III | Yes | 64.28 | 1 | Medium | 2 |
| E-MTAB-6134 | G003737_C11.CEL | III | Yes | 4.08 | 1 | Medium | 3 |
| E-MTAB-6134 | G005018_D10.CEL | III | Yes | 28.98 | 1 | Medium | 2 |
| E-MTAB-6134 | G005225_D06.CEL | III | Yes | 26.51 | 1 | High | 3 |
| E-MTAB-6134 | G011035_F01.CEL | III | Yes | 3.26 | 0 | High | 3 |
| E-MTAB-6134 | G011143_E03.CEL | III | Yes | 112.27 | 0 | Low | 2 |
| E-MTAB-6134 | G011185_C05.CEL | IIB | Yes | 9.61 | 1 | High | 3 |
| E-MTAB-6134 | G01386_F05.CEL | IIA | Yes | 37.01 | 1 | Medium | 2 |
| E-MTAB-6134 | G014418_G01.CEL | III | Yes | 7.3 | 1 | High | 3 |
| E-MTAB-6134 | G018135_G08.CEL | III | Yes | 6.35 | 1 | High | 3 |
| E-MTAB-6134 | G019322_H04.CEL | IIA | Yes | 15.13 | 1 | High | 3 |
| E-MTAB-6134 | G024468_F10.CEL | IIB | Yes | 9.01 | 1 | High | 3 |
| E-MTAB-6134 | G0246_E02.CEL | III | Yes | 85.16 | 0 | Medium | 3 |
| E-MTAB-6134 | G026044_E08.CEL | III | Yes | 82.47 | 0 | Low | 2 |
| E-MTAB-6134 | G028893_H05.CEL | III | Yes | 16.48 | 1 | High | 3 |
| E-MTAB-6134 | G0320_B09.CEL | IIB | Yes | 75.03 | 0 | Medium | 2 |
| E-MTAB-6134 | G032629_F04.CEL | III | Yes | 20.46 | 1 | High | 3 |
| E-MTAB-6134 | G032862_G03.CEL | III | Yes | 6.32 | 1 | High | 3 |
| E-MTAB-6134 | G033604_H03.CEL | III | No | 32.73 | 1 | High | 3 |
| E-MTAB-6134 | G035973_C06.CEL | III | Yes | 13.68 | 1 | Medium | 2 |
| E-MTAB-6134 | G036296_E05.CEL | III | Yes | 12.17 | 1 | High | 3 |
| E-MTAB-6134 | G036578_D01.CEL | III | Yes | 7.47 | 1 | Low | 2 |
| E-MTAB-6134 | G038351_F05.CEL | III | Yes | 17.01 | 1 | Low | 1 |
| E-MTAB-6134 | G038555_F12.CEL | IIA | Yes | 5 | 1 | High | 3 |
| E-MTAB-6134 | G0390184_G02.CEL | III | Yes | 23.75 | 1 | Low | 2 |
| E-MTAB-6134 | G039143_B03.CEL | III | Yes | 18.52 | 1 | High | 3 |
| E-MTAB-6134 | G045656_E06.CEL | III | Yes | 60.79 | 0 | Medium | 2 |
| E-MTAB-6134 | G046128_B05.CEL | IIA | Yes | 43.42 | 1 | Medium | 3 |
| E-MTAB-6134 | G046800_F02.CEL | III | No | 25.2 | 1 | Low | 1 |
| E-MTAB-6134 | G047678_C07.CEL | III | Yes | 50.95 | 1 | Low | 1 |
| E-MTAB-6134 | G047724_F03.CEL | IIA | Yes | 67.6 | 1 | High | 3 |
| E-MTAB-6134 | G047983_B08.CEL | IIB | Yes | 62.76 | 1 | High | 1 |
| E-MTAB-6134 | G049138_A12.CEL | III | Yes | 7.2 | 1 | High | 3 |
| E-MTAB-6134 | G0586_G11.CEL | III | Yes | 30.2 | 0 | Medium | 2 |
| E-MTAB-6134 | G974149_C06.CEL | IIB | Yes | 10.33 | 1 | High | 2 |
| E-MTAB-6134 | G995909_E04.CEL | IIA | Yes | 124.38 | 0 | Low | 2 |
| E-MTAB-6134 | G996696_C10.CEL | III | Yes | 8.36 | 1 | High | 3 |
| E-MTAB-6134 | H00C260530_D08.CEL | IIB | No | 44.18 | 1 | High | 3 |
| E-MTAB-6134 | H00C266400_G10.CEL | IIA | Yes | 117.53 | 0 | Medium | 2 |
| E-MTAB-6134 | H00C270878_D02.CEL | III | Yes | 13.36 | 0 | Low | 2 |
| E-MTAB-6134 | H01C288326_D05.CEL | III | Yes | 1.64 | 0 | Low | 1 |
| E-MTAB-6134 | H01C289056_H12.CEL | IIA | Yes | 93.49 | 0 | Low | 1 |
| E-MTAB-6134 | H01C294993_G04.CEL | III | Yes | 43.29 | 1 | High | 3 |
| E-MTAB-6134 | H02C295168_A01.CEL | III | Yes | 99.93 | 0 | Low | 2 |
| E-MTAB-6134 | H02C296133_D03.CEL | III | Yes | 3.98 | 1 | High | 2 |
| E-MTAB-6134 | H02C302421_B07.CEL | III | Yes | 66.32 | 0 | High | 2 |
| E-MTAB-6134 | H02C306049_H01.CEL | III | Yes | 42.47 | 0 | Low | 1 |
| E-MTAB-6134 | H02C308007_C01.CEL | III | No | 31.78 | 1 | High | 1 |
| E-MTAB-6134 | H02C309293_C03.CEL | IIB | Yes | 15.92 | 0 | High | 3 |
| E-MTAB-6134 | H02C311550_A06.CEL | III | No | 46.38 | 1 | Low | 2 |
| E-MTAB-6134 | H02C910402_D11.CEL | IIA | No | 18.49 | 0 | Low | 1 |
| E-MTAB-6134 | H02G6030_A09.CEL | III | Yes | 3.13 | 1 | High | 3 |
| E-MTAB-6134 | H03C313370_F05.CEL | III | Yes | 35.53 | 1 | Low | 1 |
| E-MTAB-6134 | H03C313891_E11.CEL | III | Yes | 2.86 | 0 | High | 3 |
| E-MTAB-6134 | H03C316329_C12.CEL | IIA | Yes | 21.81 | 1 | High | 3 |
| E-MTAB-6134 | H03C317988_D12.CEL | IIB | Yes | 77.37 | 1 | Low | 3 |
| E-MTAB-6134 | H03C324003_E05.CEL | III | No | 16.97 | 1 | Low | 1 |
| E-MTAB-6134 | H03C324710_H05.CEL | III | Yes | 5.92 | 1 | High | 3 |
| E-MTAB-6134 | H04C330331_B07.CEL | III | Yes | 19.14 | 1 | High | 2 |
| E-MTAB-6134 | H04C339280_F09.CEL | III | Yes | 51.38 | 0 | Low | 1 |
| E-MTAB-6134 | H05C11814_B12.CEL | IIA | Yes | 49.08 | 0 | Low | 3 |
| E-MTAB-6134 | H05C15376_D04.CEL | III | Yes | 9.21 | 1 | High | 2 |
| E-MTAB-6134 | H05C16724_F07.CEL | III | Yes | 11.25 | 1 | Medium | 2 |
| E-MTAB-6134 | H05C5681_D11.CEL | III | No | 10.59 | 0 | Low | 1 |
| E-MTAB-6134 | H05C6413_E12.CEL | IIA | Yes | 6.25 | 1 | High | 3 |
| E-MTAB-6134 | H05C9206_A05.CEL | IIA | Yes | 16.22 | 1 | Low | 1 |
| E-MTAB-6134 | H05C9655_F09.CEL | III | Yes | 30.46 | 1 | Medium | 2 |
| E-MTAB-6134 | H05G3182_A12.CEL | IIB | Yes | 54.11 | 0 | Medium | 3 |
| E-MTAB-6134 | H05G3531_C04.CEL | III | Yes | 33.32 | 1 | High | 1 |
| E-MTAB-6134 | H05G4354_C05.CEL | III | No | 58.98 | 0 | Low | 3 |
| E-MTAB-6134 | H05G5875_B02.CEL | III | Yes | 25.26 | 1 | Low | 2 |
| E-MTAB-6134 | H05G6567_H10.CEL | IIA | Yes | 11.78 | 1 | Medium | 3 |
| E-MTAB-6134 | H05G7150_G01.CEL | III | Yes | 19.21 | 1 | Medium | 3 |
| E-MTAB-6134 | H05G7616_F09.CEL | IIB | Yes | 12.6 | 1 | High | 3 |
| E-MTAB-6134 | H05G7986_H11.CEL | III | Yes | 43.06 | 1 | Medium | 1 |
| E-MTAB-6134 | H05G8441_F03.CEL | IIA | No | 35.07 | 1 | Low | 3 |
| E-MTAB-6134 | H05G8531_C10.CEL | IIB | Yes | 17.3 | 1 | High | 3 |
| E-MTAB-6134 | H05G9478_E07.CEL | IIA | Yes | 53.88 | 0 | Medium | 2 |
| E-MTAB-6134 | H06C16746_D07.CEL | IIA | No | 34.34 | 1 | High | 1 |
| E-MTAB-6134 | H06G1201_F11.CEL | III | No | 3.26 | 0 | Medium | 2 |
| E-MTAB-6134 | H06G3022_E05.CEL | III | Yes | 33.55 | 1 | Low | 3 |
| E-MTAB-6134 | H06G3089_B05.CEL | III | Yes | 15.39 | 0 | Medium | 3 |
| E-MTAB-6134 | H06G3384_D10.CEL | IIA | Yes | 49.7 | 0 | Medium | 3 |
| E-MTAB-6134 | H06G4855_G04.CEL | IA | Yes | 46.91 | 0 | High | 3 |
| E-MTAB-6134 | H06G4991_C07.CEL | III | Yes | 11.78 | 1 | Medium | 3 |
| E-MTAB-6134 | H06G6091_G07.CEL | III | Yes | 22.86 | 1 | Low | 3 |
| E-MTAB-6134 | H06G6462_C03.CEL | III | Yes | 21.91 | 1 | Medium | 3 |
| E-MTAB-6134 | H06G9235_H08.CEL | IIB | Yes | 32.04 | 1 | Medium | 2 |
| E-MTAB-6134 | H07C10608_C04.CEL | III | No | 20.13 | 1 | Low | 1 |
| E-MTAB-6134 | H07C17517_G09.CEL | III | Yes | 30.43 | 0 | High | 2 |
| E-MTAB-6134 | H07C2870_A07.CEL | III | Yes | 40.76 | 0 | Medium | 3 |
| E-MTAB-6134 | H07C4796_B05.CEL | III | Yes | 6.64 | 0 | Low | 1 |
| E-MTAB-6134 | H07C7336_H01.CEL | IA | Yes | 24.57 | 0 | Medium | 2 |
| E-MTAB-6134 | H07C8470_G05.CEL | III | Yes | 39.18 | 0 | Low | 1 |
| E-MTAB-6134 | H07C9483_G08.CEL | IIA | Yes | 33.62 | 1 | Medium | 3 |
| E-MTAB-6134 | H07C9595_F02.CEL | IIA | Yes | 24.11 | 1 | Low | 1 |
| E-MTAB-6134 | H07G3774_C09.CEL | III | Yes | 11.78 | 1 | High | 3 |
| E-MTAB-6134 | H07G4499_E10.CEL | III | Yes | 22.89 | 1 | Medium | 3 |
| E-MTAB-6134 | H07G4970_G03.CEL | IIA | Yes | 35.43 | 0 | Medium | 3 |
| E-MTAB-6134 | H07G5428_B07.CEL | III | Yes | 30.07 | 1 | Medium | 2 |
| E-MTAB-6134 | H07G6204_F04.CEL | III | Yes | 20.07 | 1 | Medium | 1 |
| E-MTAB-6134 | H07G6769_C02.CEL | III | No | 19.44 | 1 | Low | 1 |
| E-MTAB-6134 | H07G874_G07.CEL | III | Yes | 4.31 | 1 | High | 3 |
| E-MTAB-6134 | H08C1912_D04.CEL | IIA | Yes | 19.8 | 0 | High | 1 |
| E-MTAB-6134 | H08C337_B10.CEL | III | Yes | 21.88 | 0 | Medium | 2 |
| E-MTAB-6134 | H08C4672_A02.CEL | III | Yes | 14.08 | 1 | Medium | 3 |
| E-MTAB-6134 | H08C5473_E09.CEL | III | No | 24.28 | 1 | Low | 2 |
| E-MTAB-6134 | H08C6185_D05.CEL | IIA | Yes | 18.22 | 0 | Medium | 3 |
| E-MTAB-6134 | H08C6484_B04.CEL | III | Yes | 25.76 | 0 | Low | 2 |
| E-MTAB-6134 | H08C7056_A02.CEL | III | Yes | 27.66 | 0 | Low | 1 |
| E-MTAB-6134 | H08C7846_E04.CEL | IIA | Yes | 17.43 | 0 | Medium | 2 |
| E-MTAB-6134 | H08C852_F12.CEL | III | Yes | 5.1 | 1 | High | 2 |
| E-MTAB-6134 | H08C8737_D12.CEL | III | Yes | 20.53 | 1 | High | 3 |
| E-MTAB-6134 | H08C9275_F07.CEL | III | Yes | 23.29 | 0 | Medium | 2 |
| E-MTAB-6134 | H08C949_A05.CEL | III | No | 7.11 | 1 | Low | 2 |
| E-MTAB-6134 | H08G1028_E07.CEL | III | Yes | 10.66 | 1 | High | 2 |
| E-MTAB-6134 | H08G1478_G06.CEL | III | Yes | 27.8 | 0 | Medium | 2 |
| E-MTAB-6134 | H08G1891_G05.CEL | III | Yes | 27.3 | 0 | Medium | 3 |
| E-MTAB-6134 | H08G2028_A03.CEL | III | Yes | 6.18 | 1 | High | 3 |
| E-MTAB-6134 | H08G358_D03.CEL | IIA | Yes | 28.98 | 0 | High | 3 |
| E-MTAB-6134 | H08G3612_A08.CEL | III | Yes | 8.75 | 1 | High | 2 |
| E-MTAB-6134 | H08G3619_G10.CEL | III | Yes | 25.23 | 0 | High | 3 |
| E-MTAB-6134 | H08G496_H08.CEL | III | Yes | 28.82 | 0 | Low | 2 |
| E-MTAB-6134 | H08G5874_A04.CEL | III | Yes | 22.07 | 0 | Low | 2 |
| E-MTAB-6134 | H08G6090_B12.CEL | III | Yes | 21.81 | 0 | Medium | 3 |
| E-MTAB-6134 | H08G705_F08.CEL | IIB | Yes | 9.61 | 1 | High | 3 |
| E-MTAB-6134 | H08G7115_C11.CEL | III | Yes | 21.05 | 1 | Low | 2 |
| E-MTAB-6134 | H08G7309_H02.CEL | III | Yes | 20.16 | 0 | Low | 2 |
| E-MTAB-6134 | H08G8055_G09.CEL | IIA | Yes | 19.28 | 0 | Medium | 3 |
| E-MTAB-6134 | K02C306814_E10.CEL | IIA | Yes | 22.47 | 1 | Medium | 2 |

**Table. S5.**

PDAC hypoxia signature

| ABCB1 | CCND1 | FGF2 | MMP2 | PTGES | TLR2 |
| --- | --- | --- | --- | --- | --- |
| ACE | CCR7 | GAPDH | MUC1 | RECK | TUBB3 |
| ACE2 | CDH1 | HBEGF | MUC3A | SDHB | TWIST1 |
| ALDOA | CTGF | HIF1A | NR3C1 | SERPINE1 | VEGFB |
| ALOX5AP | CXCR4 | ITGB2 | NR4A1 | SLC2A1 | VHL |
| APEX1 | E2F3 | LDHA | NT5E | SOCS1 | VIM |
| ASS1 | EDN1 | LOX | PFKFB3 | STAT3 | ZNF217 |
| BAX | EGLN3 | MET | PGK1 | TFRC |  |
| BID | ENO1 | MICA | PIK3CA | TGFB3 |  |
| CA9 | ERCC2 | MIF | PLAU | TIMP2 |  |

**Table. S6.**

Cross-correlated features from Integrated proteome, metabolome, and lipidome data

| **Proteins** | **Metabolites** | **Lipids** |
| --- | --- | --- |
| TAF6 | Methylmalonic acid | PI 31:1 |
| LAS1L | L-Carnitine | PI 34:1 |
| RCOR1 | α-Ketoglutaric acid | Cer 42:0 |
| RPE | Succinic semialdehyde | DAG 33:0 |
| FKBP2 | Adenosine triphosphate ATP | DAG 32:0 |
| CNPY4 | 5-S-Methyl-5-thioadenosine | DAG 34:0 |
| RBBP7 | L-Lactic acid | PO 40:7 |
| GANAB | Ureidosuccinic acid | PG 38:7 |
| TAOK1 | Uracil | PC 30:2 |
| PLOD2 | α-Aspartyl-4-hydroxyproline | PG 36:2 |
| P4HA1 | Alanine |  |
| HYOU1 | Glycine |  |
| FKBP9 |  |  |
| HSP90B1 |  |  |
| PDIA3 |  |  |
| ACOT9 |  |  |
| P4HA2 |  |  |
| DNAJB11 |  |  |
| CALU |  |  |
| YTHDF3 |  |  |
| PTPN21 |  |  |
| NMRAL1 |  |  |

**Supplementary Video S1:** Live cell time-lapse images in untreated condition.

**Supplementary Video S2:** Live cell time-lapse images in treated condition upon LDHA-i.
