# Supplementary material for: Glycolytic heterogeneity drives metabolic-targeted therapy in pancreatic ductal adenocarcinoma
Source: Signal Transduct Target Ther. 2026 Jan 20;11:25. doi: 10.1038/s41392-025-02546-8 (PMC12816621; doi:10.1038/s41392-025-02546-8)

1) PL45
2) SW1990
3) PANC-1
4) MIAPaCa-2
5) HPAF-II

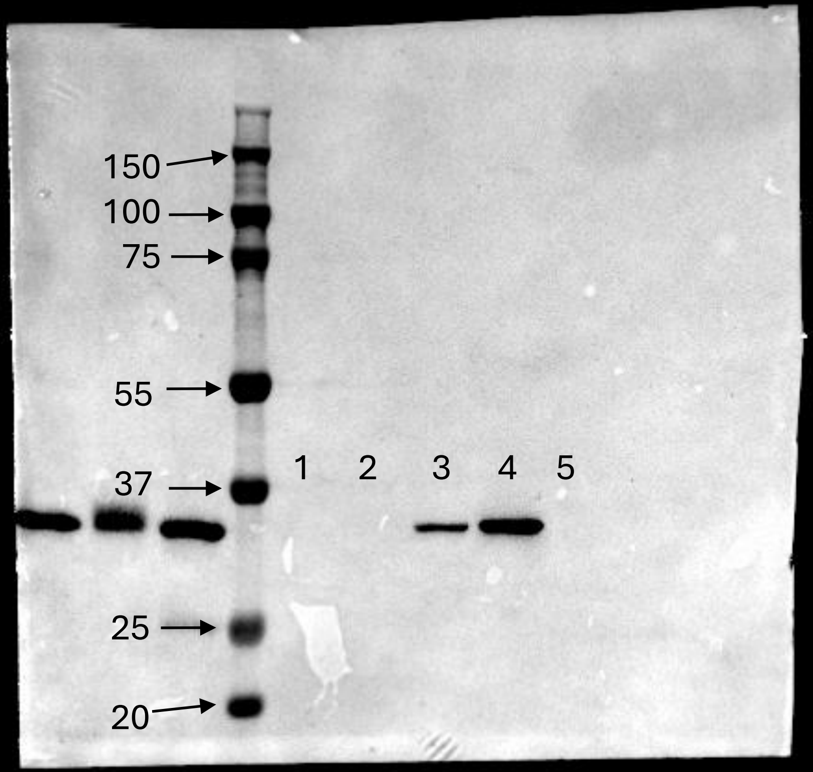

LDHA

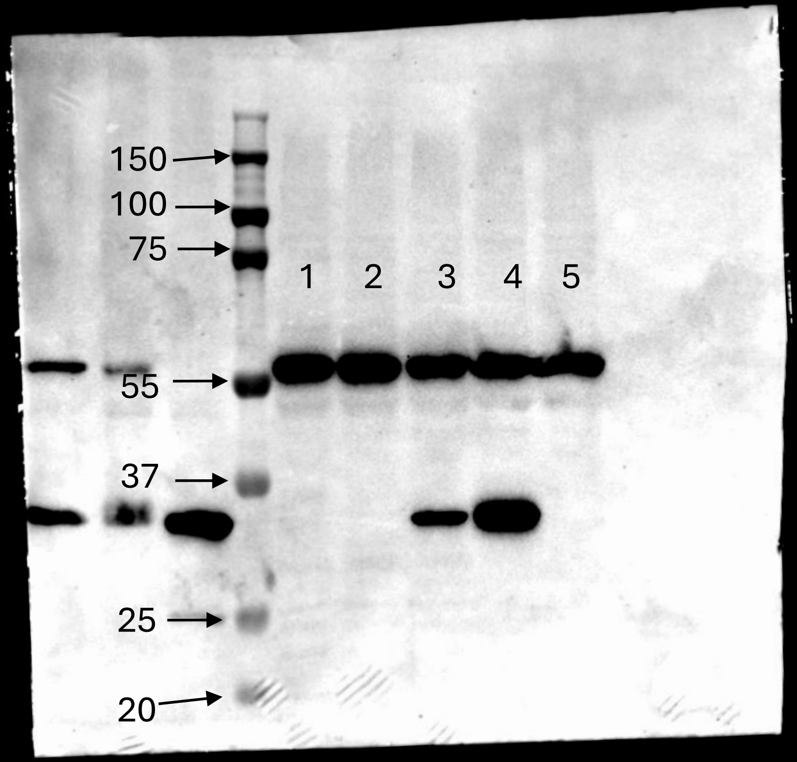

α-Tubulin

1) PL45 CTR
2) PL45 24H
3) PL45 48H
4) PL45 72H
5) MIAPaCa-2 CTR
6) MIAPaCa-2 24H
7) MIAPaCa-2 48H
8) MIAPaCa-2 72H

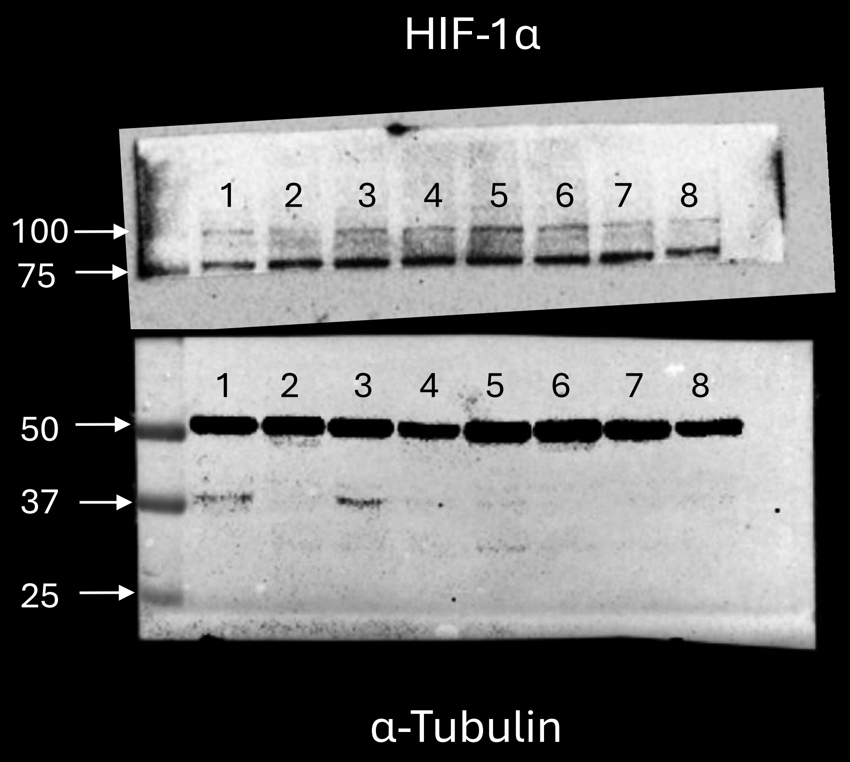

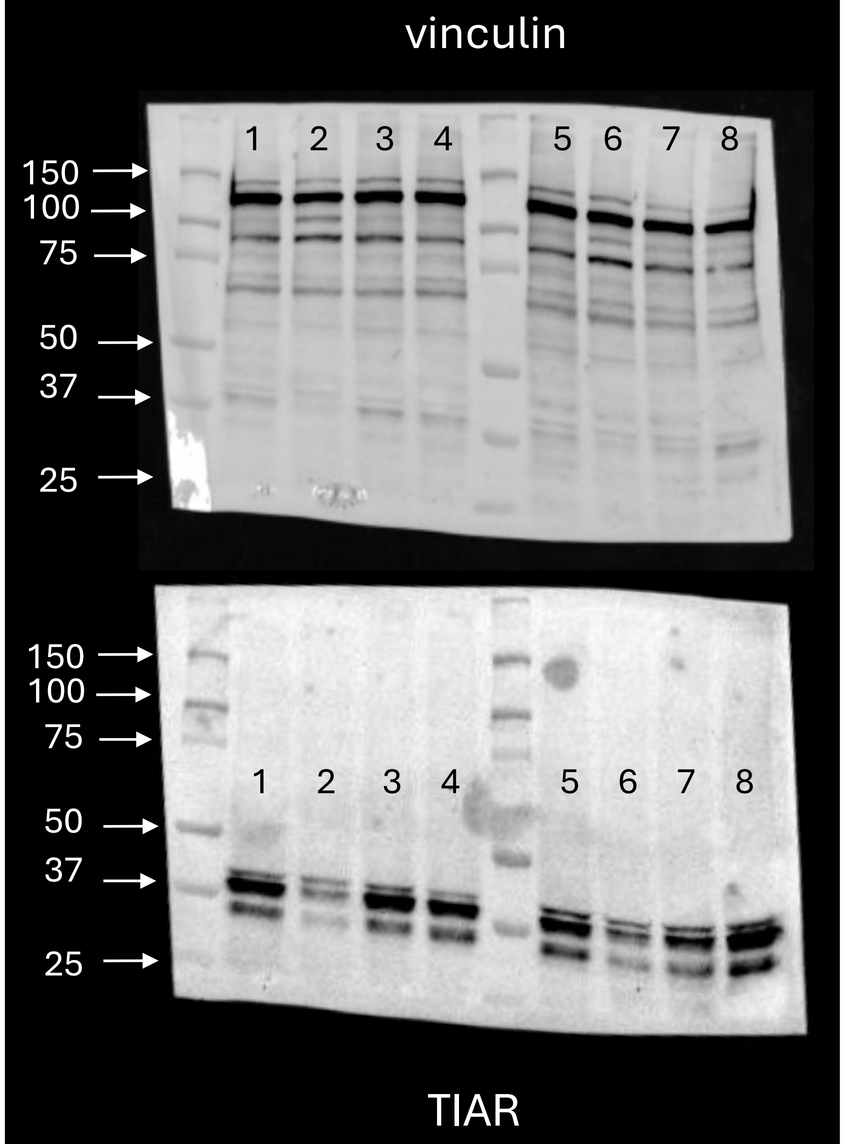

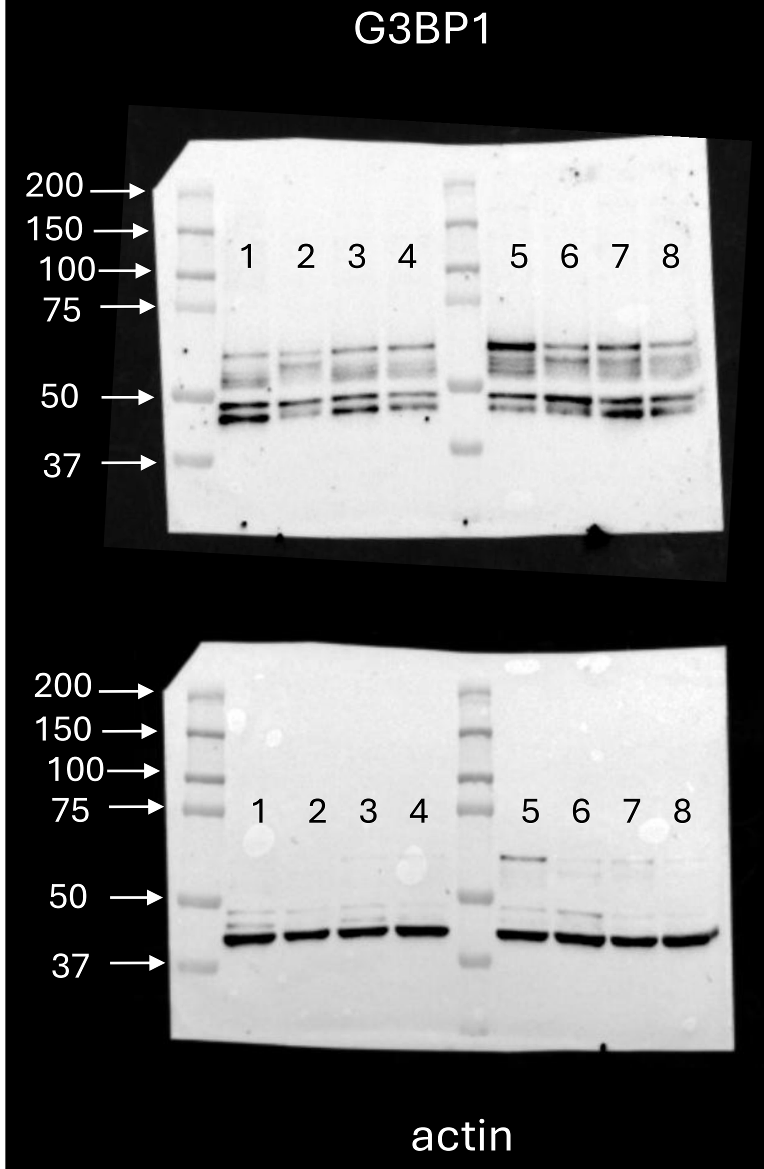

Supplement: Supplementary file 2 — Original files Western Blot [file 41392_2025_2546_MOESM2_ESM.pdf]
